# Dengue: recent past and future threats

**DOI:** 10.1098/rstb.2013.0562

**Published:** 2015-04-05

**Authors:** David J. Rogers

**Affiliations:** Department of Zoology, University of Oxford, South Parks Road, Oxford OX1 3PS, UK

**Keywords:** risk maps, dengue, *Aedes aegypti*, *Aedes albopictus*, global circulation models, corr-max rotation

## Abstract

This article explores four key questions about statistical models developed to describe the recent past and future of vector-borne diseases, with special emphasis on dengue:

(1) How many variables should be used to make predictions about the future of vector-borne diseases?
(2) Is the spatial resolution of a climate dataset an important determinant of model accuracy?
(3) Does inclusion of the future distributions of vectors affect predictions of the futures of the diseases they transmit?
(4) Which are the key predictor variables involved in determining the distributions of vector-borne diseases in the present and future?

Examples are given of dengue models using one, five or 10 meteorological variables and at spatial resolutions of from one-sixth to two degrees. Model accuracy is improved with a greater number of descriptor variables, but is surprisingly unaffected by the spatial resolution of the data. Dengue models with a reduced set of climate variables derived from the HadCM3 global circulation model predictions for the 1980s are improved when risk maps for dengue's two main vectors (*Aedes aegypti* and *Aedes albopictus*) are also included as predictor variables; disease and vector models are projected into the future using the global circulation model predictions for the 2020s, 2040s and 2080s. The Garthwaite–Koch corr-max transformation is presented as a novel way of showing the relative contribution of each of the input predictor variables to the map predictions.

## 1. Introduction

When, in 1930, Wolgang Pauli first hypothesized the existence of a new particle (later to be called the neutrino by Enrico Fermi) he wrote apologetically to his fellow physicists:

I have done something very bad today by proposing a particle that cannot be detected; it is something no theorist should ever do.

Pauli described his idea as ‘a desperate remedy’ to account in full for the observed loss of energy during radioactive beta decay. Pauli was concerned that if his new particle could not be detected his prediction could not be disproved [1].

When ecologists and epidemiologists try to predict the future of biological systems they are in a somewhat similar position to Pauli. They are making predictions which cannot be disproved until the future arrives. Predictions without the possibility of disproof cannot be regarded as science, even if the methods used to make them follow good scientific practices. It is difficult to know which the greater sin is: to propose something you think can never be disproved (Pauli), or to propose something you know cannot be disproved until the distant future arrives (risk maps for diseases using future climate predictions).

In this article, global risk maps are developed for the important vector-borne disease dengue, using both recent past meteorological variables and their predicted values under alternative future climate scenarios. This is done not only to show risk maps for this disease in the recent past and future but also, and more importantly, to examine some of the scientific practices used to make predictions about the future of all vector-borne diseases, in order to answer the following important questions:

— How many variables should be used to make predictions about the future of vector-borne diseases?
— Is the spatial resolution of a climate dataset an important determinant of model accuracy?
— Does inclusion of the future distributions of vectors affect predictions of the futures of the diseases they transmit?
— Which are the key predictor variables involved in determining the distributions of vector-borne diseases in the present and future?

Here, only climatic constraints on vector and disease distributions are considered, to highlight the complexities of the limited set of questions posed. Other non-climatic variables are often important for vector-borne diseases and some of these are discussed elsewhere. For example, other environmental and socio-economic changes are considered in this special issue by Parham *et al*. [2] and elsewhere by [3,4], public health policy by [5,6] and interactions between water, agriculture, ecosystems and health (represented by malaria) by Piontek *et al.* [7]. This article concentrates on statistical descriptions of disease distributions; examples of biological approaches to the same challenge are given here in articles [6,8–10]. Here, only a single suite of future climate scenarios are considered, not ensembles, in order to illustrate the four key questions above; variability between ensemble predictions and what we may learn from them are highlighted in article [11]. Finally, the models developed here assume that the future is quantitatively different from the past, but not qualitatively different. Evolution within and between disease systems will introduce such qualitative differences, as will the development of new vaccines, and some of these issues are dealt with here in article [12]. Most of these further complexities must eventually address questions of a similar sort to those above.

Dengue is an important vector-borne tropical disease that threatens one-third of humanity on the planet [13]. Because of its importance, a number of attempts have been made to produce global risk maps for it, based on what is known about the relationship between dengue, climate and other environmental factors [4,14–17]. While some of these maps are based on our rather limited biological knowledge of the key constraints operating on dengue and its vectors, most use one or other of a variety of statistically based approaches, including logistic regression [18], generalized additive models [19], random forest models [13] or nonlinear discriminant analysis [20], using as predictors climate or satellite remotely sensed environmental and other data. A recent comparison between different dengue risk maps, using the Fleiss *kappa* measure of similarity [21], showed only moderate agreement between the predictions for Asia, fair agreement for the Americas and only slight agreement for Africa; globally, the overall agreement was classified as only fair [20]. Differences between the risk maps can be attributed to the use of different dengue databases, different modelling approaches and different sorts of predictor variables. Such disparity between various predictions of dengue's distribution at the present time, however, provides a strong warning. If we cannot get the present right, what chances are there that we can correctly predict the future?

## 2. Material and methods

### (a) Disease and vector data

The databases for dengue and its two principal vectors, *Aedes* (*Stegomyia*) *aegypti* (L.) and *Aedes* (*Stegomyia*) *albopictus* (Skuse) (Diptera: Culicidae), were those used by Rogers *et al*. [20], derived from literature searches restricted to the time period 1960–2010. References were searched for the geographical location of the disease or vectors and place names were geolocated using a variety of online gazetteers, resulting in a total of 1400 presence records for dengue (dengue fever or dengue haemorrhagic fever), 200 records for *Ae. aegypti* and 500 records for *Ae. albopictus,* all at a geographical resolution of one-sixth degree, the finest resolution used in the models developed here. The original dengue and vector records were referred either to point locations, or to polygon centres when the observations came from areas rather than points, usually administrative level 2 (where administrative level 0 is the country). The presence points for the three species are shown in map form in the electronic supplementary material.

Most disease and vector databases contain few records of confirmed disease or vector absence. For the present exercise, sets of pseudo-absence points were generated for each database by selecting at random points no less than 0.5 degrees and no greater than 5 degrees away from any presence point. The lower threshold ensures exclusion of absence points which are likely to show similar climatic conditions to the nearby presence points (a topic explored by Chefaoui & Lobo [22]) and the higher threshold ensures that the sampled climatic conditions are not so different from any presence area as to be unhelpful in discriminating precisely between areas of presence and absence. Other ways of choosing pseudo-absence points are based on distance in environmental as well as geographical space [20], but these methods were not applied here, because all of the modelling was done at a relatively coarse spatial resolution. Four thousand random pseudo-absence points were generated for dengue, 3000 for *Ae. aegypti* and 4000 for *Ae. albopictus*. The full collection of presence and pseudo-absence points for each situation is referred to here as a ‘training set’.

### (b) Environmental data

In a previous paper, the author used satellite derived environmental data at a resolution of 1/15th of a degree to make global risk maps of dengue [20]. For present purposes, however, series of ground-based, long-term meteorological data were selected, or the output of global circulation models (GCMs) predicting the climate both of the recent past and in the future under various scenarios of climate change.

### (c) Meteorological data

For comparison with previous predictions of dengue's distribution using a meteorological dataset [18], the present analysis used one-sixth degree ‘climate norms’ (i.e. synoptic monthly averages) for climate of the recent past, 1961–1990 [23], a dataset which includes monthly values of precipitation, wet-day frequency, temperature, diurnal temperature range, relative humidity, sunshine duration and ground frost frequency. Hales *et al*. [18] had used an earlier version of the same dataset (with vapour pressure instead of relative humidity), covering the same 1961–1990 period, at a coarser spatial resolution of 0.5 degrees [24,25].

### (d) Global circulation model climate data

GCMs are built upon descriptions of energy fluxes in the global atmosphere interacting with land and sea masses according to well-defined physical principles. An accurate GCM should be able to predict the future of the Earth's climate, with or without anthropogenic forcings, and can therefore forewarn of the likely dangers of such forcings in the present century and beyond. The complexity of global systems, however, means that early GCMs were unable to capture many features of the Earth's climate, especially regional or hemispheric events with global consequences (such as El Niño, or the North Atlantic oscillation, NAO). More recent models are more accurate although they still cannot capture the full extent, precise timing or location of such events [26,27]. Whereas earlier versions of GCMs made predictions of future climate by adding the modelled differences between the future and present day to the observed present day climate (the latter from meteorological records), many of the more recent GCMs claim sufficient accuracy of prediction for their outputs to be used to describe directly both the present day and future climates under a variety of future scenarios. The third generation of the Hadley Centre model (HadCM3) is of this type [28]. The model is able to show what have been the effects on current climate of human-generated greenhouse gas emissions over the past century (i.e. the model is run with and without these emissions, and the results compared). The model is then allowed to run on into the present century, to make predictions of future climates assuming various emissions scenarios. Model outputs are available at a variety of temporal and spatial resolutions.

For present purposes, a range of the HadCM3 model outputs were selected (http://www.metoffice.gov.uk/research/modelling-systems/unified-model/climate-models/hadcm3). These have been used in the last three IPCC assessment reports (AR3 to the current AR5). While the HadCM3 model has been developed (to HadCM4) since the publication of the IPCC fourth assessment report (AR4), there are questions about the accuracies of some of the predictions of the latter model. HadCM3 still ranks highly compared with other GCM models [29].

Full details of the HadCM3 model are given in reference [28]. Data from the A1F1, A2, B1 and B2 scenarios were found on the IPCC Data Distribution Centre website (http://www.ipcc-data.org/, last accessed February 2014), for the periods centred on the recent past (the 1980s, hereafter referred to as the ‘present day’), the 2020s, the 2040s and the 2080s. Most (though not quite all) scenarios and periods had predictions for mean, maximum and minimum temperature, for relative humidity and for precipitation. All of these files were downloaded and turned into image files (the original files are in text format) at the native resolution of 3.75 by 2.5 degrees and then cubic spline-interpolated to a resolution of 0.5 degrees before further processing.

GCM predictions for present-day climate are very similar under the different emissions scenarios because they are all identically forced up to 1989 [28]. The predictions diverge more, the more into the future they are projected. Although the precise rank ordering of effects in the different scenarios changes a little over time, in general, scenario B1 (based on maximum reduction of emissions) shows the smallest changes (for example an increase in surface air temperature of 1.54°C by the 2040s and 2.38°C by the 2080s), whereas scenario A1F1 (with relatively unrestrained emissions) shows the largest changes (increases of 2.03°C and 4.26°C for the same periods). These figures are for global (land + sea) temperatures, and we are currently more interested in increases on land. By the 2040s, land air temperatures rise by 3.37°C in the B1 scenario and 6.20°C in the A1F1 scenario (table 4 in reference [28]). These changes are considerable and must have an impact on many biological processes. There are, in addition, commensurate changes in other variables, for example a slight decrease in rainfall on land (−0.004 mm per day) in scenario B1 and a slight increase (+0.014 mm per day) in the A1F1 scenario by the 2040s. Hence, a multivariate approach to predicting dengue futures must be adopted.

All dengue or vector species' models were run using only the meteorological or the A1F1 or B1 scenario GCM output data. Both altitude and other variables such as human population density are thought to be important in determining dengue's distribution at the present time [20]. These variables were not used in the present models, however, because altitude is clearly a proxy for a mixture of temperature, rainfall and other climatic variables, and the future relationship between altitude and all these variables is uncertain. While predictions have been made about the future abundance and distribution of humans, there is similar uncertainty in these figures which only adds to the uncertainty of predictions based only on climate futures. Thus, the present models examine the more limited impacts of future climates alone.

### (e) Environmental data processing

All original meteorological and GCM output data were synoptic monthly averages for January to December. These data were temporally Fourier processed [30] to extract from each time series a set of orthogonal (i.e. uncorrelated) data to be used as descriptor variables in the vector and disease models, as described in references [30,31]. Temporal Fourier variables included the mean value, the amplitudes and phases of the annual, bi-annual and tri-annual cycles of change, the Fourier minimum and maximum and the variance of the original data series, a total of 10 variables from each observed climate or GCM variable and therefore a grand total of 70 variables from the seven selected variables of the 1961–1990 meteorological dataset. This collection of variables forms a unique ‘finger-print’ of the climatic conditions in any particular site, each variable amenable to a simple, biologically relevant interpretation, unlike the products of other data reduction and ordination techniques such as principal components analysis.

### (f) Distribution modelling

Rogers *et al*. [20] provide a brief review of spatial modelling techniques (dealt with in more detail in reference [32]) and many of the available statistical models have been compared quantitatively by Elith *et al.* [33–36] in a series of valuable papers. Furthermore, some of the topics covered here for vector-borne diseases have already been investigated for species in general, such as the effect of spatial resolution on model accuracy [37,38], and the effects of selecting pseudo-absence sites in different places [22]. Other important topics tend to be very specific to the species distribution model applied; for example, the estimation of predictor variable importance [39].

The technique of nonlinear discriminant analysis is used for all the models presented here [30,31,40]. This approach sits conveniently between the many, purely statistical models of species' distributions and the very many fewer biologically based models. Statistical models have been criticized because it is claimed that they cannot take into account biologically important thresholds, breakpoints and nonlinear relationships between the environment and key demographic variables that are at the heart of many biological processes. While this criticism is valid for some statistical approaches, such as various tree-based modelling approaches (where each node on the tree identifies a key value of one or more environmental variable that splits a subset of the data into presence and absence categories; the same variable may appear in several different places and levels within the tree, and with different key values, making biological interpretation difficult), nonlinear discriminant analysis is based on the assumption of a multivariate normal response of a species to climatic variables, and can allow for thresholds, breakpoints and nonlinearities. Key here is the fact that nonlinear discriminant analysis allows the observations to be split into a number of clusters (for both presence and absence observations), thus accommodating a limited but realistic variety of responses of species to environmental variables.

Briefly, data were extracted for all the Fourier processed variables at each presence and pseudo-absence site in the three training sets. Before analysis, the data were clustered, using the *k-means* clustering algorithm in SPSS v22 (©IBM Corporation and others), into between one and eight clusters each for presence and absence. Obviously, the more clusters used, the more likely it is that each cluster will be multivariate normal, and therefore the more appropriate will discriminant analytical methods be. Too many clusters, however, will reduce the sample size of each cluster, and make biological interpretation of the results difficult. In the present models, either two absence and one presence clusters were used (all *Ae. aegypti* models), or three absence and two presence clusters (all *Ae. albopictus* models), or three absence and three presence clusters (dengue models). These cluster numbers were chosen after initially running models with a variety of cluster combinations and examining the accuracy of the outputs.

With one exception (model results shown in figure 5), all models were run using a bootstrap approach whereby a series of subsamples (usually 300 presence and 300 pseudo-absence points; or 200 of each for the coarsest resolution model in figure 2) were selected from each training set (points selected at random, with replacement) and a model developed for each subsample. One hundred such models were run for each situation, and the results combined at the end to produce a single output risk map.

Each model was run using a stepwise inclusion method for variable selection. Thus, on the first round, in each model, each available environmental variable was examined in turn to see how well it could discriminate between presence and absence points. At the end of the round, the best discriminating variable was selected and the second round began. This second round selected from among all remaining variables the single one that, together with the first variable, again gave the best discrimination of presence and absence points. This continued until 10 variables were selected for each model.

There are many criteria for deciding on the goodness of fit during each round of modelling (see, for example, table 1 and discussion in [31]). The present models either maximized *kappa*, the index of agreement between prediction and observation [41], or minimized the corrected Akaike information criterion (AICc), a statistic that is penalized on the basis of the number of predictors (environmental variables) within the model, and so allows choice of the optimum number of predictor variables rather than any higher number that tends to result in over-fitting [42]. Stepwise inclusion allowed the eventual calculation of the importance of each predictor in the bootstrap models. Thus, the first selected variable was given a rank of one, the second a rank of two and so on; variables not included in the selected set of 10 variables were given a rank of 11. At the end of each 100 bootstrap model run, the average ranks were calculated for each variable in turn to identify which variables overall were most important in the risk map predictions. It should be noted that stepwise inclusion as applied here does not encounter the common problem of stepwise methods used elsewhere in ecology [43] because it does not involve frequentist statistical tests to accept or reject variables.

**Table 1.** Accuracy statistics for the dengue models shown in figure 1 with (*a*) one, (*b*) five and (*c*) a maximum of 10 variables. *Kappa, kappa* index of agreement; %correct, overall correct predictions (%); %PPV, positive predictive value (=consumer's accuracy for presence sites) (%); %NPV, negative predictive value (=consumer's accuracy for pseudo-absence sites) (%); %Flse +ves, false-positive predictions (%); %Flse −ves, false-negative predictions (%); sens., sensitivity; spec., specificity; TSS, true skill statistic; AUC, area under curve (ROC); AICc, corrected Akaike information criterion; nvar, mean number of variables used in models (AICc-dependent); *n,* total number of models in each series. In brackets after each mean = 1 standard deviation.

no. vars	*kappa*	%correct	%PPV	%NPV	%Flse + ves	%Flse -ves	sens.	spec.	TSS	AUC	AICc	nvar	*n*
1	0.306 (0.049)	65.30 (2.44)	54.60 (7.78)	75.97 (4.98)	12.07 (2.82)	22.62 (4.39)	0.543 (0.089)	0.754 (0.056)	0.297 (0.105)	0.735 (0.018)	721.2 (17.4)	1 (0)	100
5	0.455 (0.042)	72.73 (2.10)	77.93 (4.28)	68.69 (3.43)	15.93 (1.84)	11.33 (2.13)	0.768 (0.043)	0.678 (0.037)	0.446 (0.057)	0.80 (0.017)	714.9 (36.2)	5 (0)	100
10	0.716 (0.029)	85.78 (1.44)	88.74 (2.18)	84.02 (2.50)	8.43 (1.38)	5.78 (1.15)	0.880 (0.023)	0.827 (0.028)	0.707 (0.036)	0.911 (0.014)	502.5 (51.0)	9.76 (0.64)	100

While variables were selected according to one or other of the above criteria, each output model also gave a fuller variety of accuracy measures, including percentage correct predictions, positive and negative predictive values (PPV and NPV, sometimes referred to as the consumer's accuracy for presence and absence, respectively), percentage false positives and negatives, sensitivity, specificity, the area under the curve (AUC), all defined and referenced in reference [31], and the true skill statistic (TSS [44]).

### (g) Discriminant analysis model details

In discriminant analysis, a key step of the calculations involves the Mahalanobis distance (*D*^2^_12_), a measure of separation in multivariate space, calculated as follows2.1
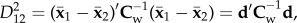
where the subscripts refer to groups 1 (e.g. for vector absence) and 2 (e.g. for vector presence), 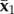 and 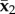 are mathematical vectors of the mean values of the variables defining each group (i.e. its centroid), 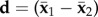 and 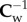 is the inverse of the within-groups covariance (dispersion) matrix [40] (the prime indicates the transpose of a row or column vector). When all variables are first standardized to their global mean values (mean = 0, s.d. = 1.0), which is assumed hereafter, 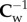 is the inverse of the correlation matrix. Thus, *D*^2^ is the Mahalanobis distance (MD) between the sample centroids adjusted for their common covariance (*D*^2^ remains the same whether or not variables are first standardized). In the simple case where the variables are not correlated (and therefore the correlation matrix consists simply of ones on its diagonal, and zeroes elsewhere), *D*^2^ reduces to the squared Euclidean distance between the centroids.

Equation (2.1) is written using the mathematical vectors of the centroids of the two distributions, and thus measures the separation between two groups of points. If one or other 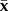 vector is replaced by the vector of values of a single point, the MD is then the distance between that single point and the centroid of the other cluster of points. This is the routine calculation carried out during the making of predictive risk maps. The MD of any new point (e.g. a pixel in a series of climatic image files) is calculated to each of the clusters defined by the training set. The point may then be assigned to the cluster to which it is closest (lowest MD). More precisely, the MD may be turned into the posterior probability of belonging to the different clusters in the analysis. This is achieved effectively by inserting the MD into the equation for the standard normal distribution of which the clusters are samples, using the following equations2.2
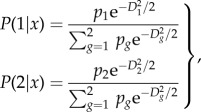
where *P*(1|*x*) is the posterior probability that observation **x** belongs to group 1 and *P*(2|*x*) the posterior probability that it belongs to group 2 [40] (the exponential terms in equation (2.2) are those of the multivariate normal distributions defining groups 1 and 2; all other terms of the multivariate distributions are the same in the numerator and denominator and therefore cancel out [45]). In equation (2.2), *p*_1_ and *p*_2_ are the ‘prior probabilities’, that is, the probabilities with which any observation might belong to either group given prior knowledge or experience of the situation. In the absence of any prior experience, it is usual to assume equal prior probabilities; thus, in the simple case of two-group discrimination, *p*_1_ = *p*_2_ = 0.5. This assumption of equal prior probabilities is made more appropriate by ensuring equal numbers of presence and absence observations in the models, for example during the selection of bootstrap samples. It has previously been shown that equal sample sizes also improve model accuracy [46].

Clearly, equation (2.2) involves a normalization step where a function of the individual cluster membership probability (the numerators in equation (2.2)) is divided by the total of all probabilities (denominators in equation (2.2)), so that they sum to 1.0. In other words, the assumption is being made that any observation must belong to one or other of the clusters defined in the model. This emphasizes the importance of carefully selecting the training set to be representative of all possible presence and absence sites, not just some of them. In general, it is advisable to produce along with the output image of predicted probabilities a second image of the MD to the nearest cluster in the training set, that is, the cluster to which each pixel is assigned. This image can then be examined to find areas where the MDs are very large and therefore where predictions are likely to be inaccurate. Alternatively, images may be produced of the MDs to the nearest (in environmental space) presence cluster; such images show the environmental suitability of each site for the vector or disease concerned and may be used to predict and monitor the spread of invasive vectors or diseases (an example is given in reference [20]).

When predictions are being made about places that are very different from any in the training set, or they are being made using climatic variables predicted under future climate scenarios, care should be taken with any predictions based on MDs that are greater than any observed in the training sets of data. In all the present models, therefore, records were kept of the maximum MD of any training set point from its cluster centroid; if sets of climatic conditions from other places or times into the future gave MD values greater than these maxima, the output pixel was classified as ‘no prediction possible’ (and coloured grey in the output imagery).

Equations (2.1) and (2.2) should be modified when the assumption of a common covariance for all clusters is obviously invalid. Not only may areas of presence and absence differ in their environmental characteristics, but different parts of a species' range may also show more subtle differences, requiring separate multivariate descriptions of their climatic conditions. Each cluster (either for presence or absence) must then be treated as a separate multivariate normal distribution, with its own covariance characteristics. Posterior probabilities are then calculated in an analogous way, by summing across all distributions. In the case of two groups only (one for presence and one for absence), equation (2.2) is then modified as follows2.3
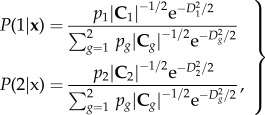
where |**C**_1_| and |**C**_2_| are the determinants of the covariance matrices for groups *g* = 1 and 2, respectively. The MDs in equation (2.3), calculated from equation (2.1), are evaluated using the separate within-group covariance matrices **C**_1_ and **C**_2_ [45]. When there is more than a single class of presence or absence data (e.g*.* multiple clusters), the summation in the denominators of equation (2.3) covers the entire set of *g* > 2 groups, and there are as many posterior probability equations as there are groups. With unequal covariance matrices, the discriminant axis (strictly speaking a plane) that separates the two groups in multivariate space is no longer linear, and equation (2.3) then effectively defines the maximum-likelihood solution to the problem [47].

### (h) Models using meteorological variables

In order to answer the first question in §1, three bootstrapped models were made for dengue using the meteorological dataset at its full resolution of one-sixth of a degree of longitude and latitude. The first model used a single climate variable, mean monthly relative humidity, for direct comparison with the results in [18]; the second model always used the same five variables; mean monthly values of relative humidity, rainfall, maximum, minimum and mean temperature, again for comparison with [18]. In the third model, all 70 Fourier variables were made available to each bootstrap model, which selected a maximum of 10. This third model should therefore show the best performance possible with this meteorological dataset, for comparison with the other two models with restricted datasets.

To answer the second question in §1, models were run with both the training set data and the meteorological data successively aggregated into pixel sizes of one-third, one half, one and two degrees' resolution. The training set data were first simply projected onto a blank image at each resolution; multiple hits of points on a single pixel were recorded only as presence or as absence in that pixel, and presence always took priority over absence; thus, a training set was created for each resolution, but derived from the full resolution training set of both presence and absence points. The meteorological data were simply averaged from the original resolution of these data (one-sixth of a degree) to the target pixel sizes, taking care to exclude any sea pixels from the averaging process.

### (i) Models using global circulation model predictions of climate futures

The third and fourth questions in §1 involved use of the GCM predictions for present and future climates. Models were first made for dengue on its own (i.e. without the vectors) and for each of the two vector species, all based on the GCM predictions of the present-day climate (i.e. the 1980s). These models, for both dengue without vectors and for the two vector species, are based on a maximum of nine variables (the mean, maximum and minimum values of rainfall, relative humidity and temperature). Once the vector models were completed, they were used in a further set of dengue models (dengue + vectors) which also included the same nine climatic variables (i.e. 11 variables in total). The dengue + vector models might therefore include the predicted vector distributions; they would only do so if the vector layers were useful as predictors discriminating dengue presence and absence areas which, often, they were.

All models were then re-run using the established relationships between dengue or its vectors and climate but with the future climate scenarios as the climate predictor layers. This gave a series of predictions of the future distribution of the two vector species on their own and of dengue, with or without its vectors, under the two selected scenarios of climate change for the 2020s, 2040s and 2080s. Thus, predictions were made for (dengue + climate change), or for (dengue + vectors + climate change). These two predictions for the future distribution of dengue will differ only if the vectors respond differently to climate change than does dengue. Any differences between predictions will determine how much in future statistical models for vector-borne diseases need to consider both the diseases and their vectors when making predictions of the effects of changing climates.

When making predictions in the GCM series of models, it was decided to restrict the number of predictor variables in each bootstrapped model to the optimum number as determined by the AICc values for that model. This was often very many fewer than the maximum number of variables available, because there are strong correlations (*r* > 0.9) between some of the input variables (for example between the mean and both the maximum and minimum of each climate variable), rendering them more or less redundant for prediction purposes.

### (j) Establishing the importance of predictor variables—the Garthwaite–Koch transformation

The final question in §1, about how to identify the most important predictor variables determining a species' presence or absence in an area, would be easy to answer if all the predictors were orthogonal to (i.e. uncorrelated with) each other, and the MD reduced to the Euclidean distance. It is less easy to answer when variables are correlated with each other. What is needed is a decomposition of the MD of equation (2.1) along the following lines:2.4
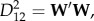
where **W** is a mathematical vector with as many terms as there are variables contributing to 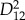. Equation (2.4) is suitable for present requirements if and only if (i) the components of **W** are uncorrelated with each other and (ii) each one of them can be uniquely associated with one, and only one, of the *x* variables contributing to 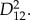 Garthwaite and Koch, developing from [48], have recently proposed a method called the corr-max transformation that achieves these objectives [49]. They show that **W** can be calculated from:2.5
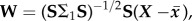
where **S** is a diagonal matrix of the inverses of the standard deviations of the *x* variables, Σ_1_ is the correlation matrix , 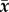 is a mathematical vector of the mean values of the *x* variables and ***X*** is a vector of values of the sample point giving the observed value of the MD to the centroid defined by 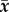. The individual elements of **W** show a close correspondence (high correlation) with the individual *x* variables (in fact, the correlation between *x_i_* and *w_j_* is the (*i,j*) element of 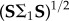, and the transformation maximizes the sum of the *x_i_*–*w_i_* correlations).

When there is a strong correlation (greater than 0.9) between some of the original *x* variables, the right-hand side of equation (2.5) should be pre-multiplied by an orthogonal matrix that rotates the correlated variables (and only them). This effectively replaces, for example, two correlated variables with two new variables, their sum and their difference, which will be less correlated. Interpretation of the resulting values in **W** remains the same except that the respective components now refer to the importance of the sum and difference of the correlated variables [49]. In the examples given here, care was taken to use predictor variables that were not strongly correlated with each other.

The values of the squared components of **W** (on what will be referred to as the Garthwaite–Koch scale) can be examined for their relative contribution to the overall sum (of all squared values). This is a measure of the relative contribution of the original *x* variables to the MD. In the present case, images were produced in which each pixel identified the single input variable contributing most to the MD of that pixel from the nearest (in environmental space) presence cluster centroid. The pixel's value was given the sign of the difference between the pixel's variable value and the relevant centroid's value (i.e. one element of the final bracketed term in equation (2.5)), so that the resulting images could be interrogated to answer the following question: ‘Which of the input variables is most responsible for the predicted presence or absence of the disease at this particular site, and is its value higher or lower than that of the closest centroid for presence?’ This therefore provides an answer to the fourth question posed in §1.

### (k) The coincidence of disease and climate data

Many of the records in the dengue database post-date the period for which the meteorological data apply (1961–1990). It is possible that dengue has spread beyond its 1961–1990 limits in response to recent climate changes, thus making it inappropriate to use the older climate records on the newer dengue data. Although many publications report an increasing threat of dengue globally [50,51], these threats are mostly within dengue's recent historical limits. The mean latitudes of the dengue and *Aedes* records in the present database were calculated for the five decades from 1960 to 2010. While there was a small poleward shift of the mean absolute latitude of the *Ae. albopictus* records between the periods 1961–1990 and 1991–2010 (from 22.14 ± 12.04 s.d. degrees to 24.58 ± 12.16 s.d.), there were small equator-ward shifts of the mean absolute latitude records of both *Ae. aegypti* (from 17.42 ± 8.47 s.d. degrees to 15.95 ± 8.06 s.d.) and all records of dengue (i.e. both dengue and severe dengue, DHF) (from 16.51 ± 7.41 s.d. degrees to 14.55 ± 7.49 s.d. degrees). The decadal changes in mean latitudes are shown in the electronic supplementary material. Thus, it seems acceptable to produce presence/absence risk maps for dengue and its vectors using a climate database from the 1961–1990 period, and GCM predictions for the 1980s.

### (l) The meaning of statistical models of disease distributions

The question often arises as to what exactly statistical models based on observed records of species are predicting. In contrast to biological or process-based models, which are generally predicting the species' ‘fundamental niche’ (i.e. its distribution constrained only by its physiological tolerance of temperature, humidity, etc.), statistical models are more nearly predicting the species' ‘realized niche’, the fundamental niche modified by all non-climatic variables, including competitors, predators, parasites and, in the case of vector-borne diseases, various human activities which either directly (e.g. insecticides) or indirectly (e.g. the provision of safe, piped water supplies; or changing agricultural practices [52]) affect the vectors' or disease's distribution. Regions to which a species is adapted but which it has not yet colonized are also not included in the realized niche. Thus, the realized niche determines what we see and measure of a species' distribution on the ground at the present time. Describing such realized niches by modelling is, of course, equivalent to trying to hit a moving target, because realized niches change over time, as development proceeds. In the case of vector-borne diseases, for example, realized niches have tended to contract towards tropical regions. Thus, dengue and numerous other vector-borne diseases historically had much wider distributions than they do today, including temperate as well as tropical regions [53]. The present models are for the observed global distribution (i.e. realized niche) of dengue and its vectors in the second half of the twentieth century. Projections of models into the future assume that similar levels of development will in the future be affecting the realized niches of these species in the same way that they do today. Thus, it is assumed that whatever affects dengue's realized niche at, for example, 20°C today will operate in regions experiencing 20°C in the future. This seems to be a more preferable starting point for such studies than any based on either fundamental niche predictions (which then need to incorporate all the factors limiting this to the realized niche) or on making other assumptions about how development in each climatic regime will change as those regimes shift geographically.

## 3. Results

The results section addresses in turn each of the questions posed in §1.

### (a) How many variables should be used to make predictions about the future of vector-borne diseases?

**Figure 1.**
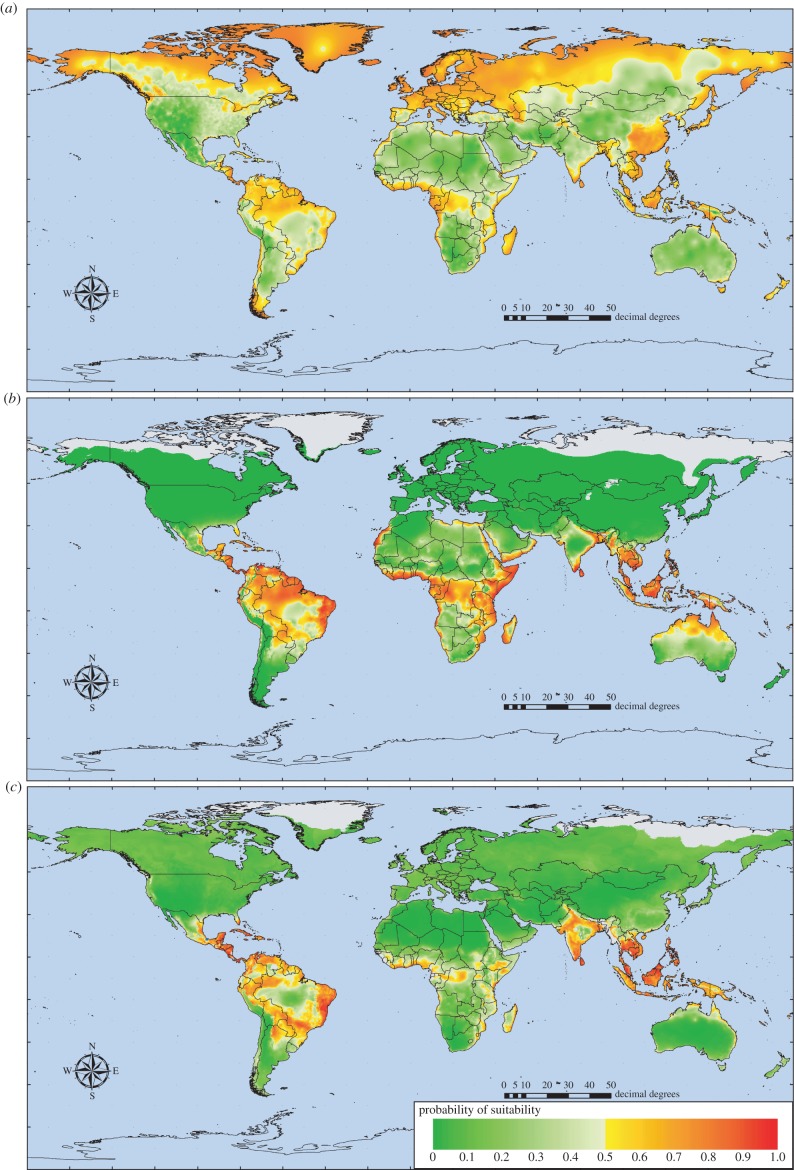
Risk maps for dengue using (*a*) one, (*b*) five or (*c*) a maximum of 10 variables (see text and table 1).

Figure 1 shows predictive risk maps for dengue from models using one, five (always the same) and a maximum of 10 (selected from a total of 70) predictor variables and table 1 shows the accuracy metrics for this series of models. Relative humidity was chosen as the single predictor (figure 1*a*) because this is related to vapour pressure, the single variable used by Hales *et al*. [18] in their models of dengue, in which they claimed an over-riding importance of vapour pressure. Figure 1*a* shows the average of 100 bootstrap model results, the only difference between models here being the bootstrap samples from the training set. In the tropics, dengue occurs in areas of higher rather than lower humidity (hence model predictions tend to be correct for these regions), but areas of high humidity also occur at high latitudes which are therefore also predicted, incorrectly, to be suitable for dengue.

The five variables selected for the next model (figure 1*b* and table 1) were mean monthly rainfall, mean monthly relative humidity, maximum, minimum and mean monthly temperature. Again, these were chosen on the basis of their similarity to variables used by [18] and they were all included in each bootstrap model in a stepwise fashion. In all 100 bootstrap models, relative humidity was never chosen first, but usually came second to rainfall which was always selected as the best discriminating variable. Spatial predictions of these models were much better, with no false positive predictions at high latitudes (figure 1*b* and table 1).

Finally, when the models could select from the full range of Fourier processed meteorological variables (figure 1*c* and table 1), the annual amplitude of rainfall was often selected as the best variable (mean rank across 100 models of 3.13, s.d. = 3.72, thus s.e. = 0.37), followed by the bi-annual amplitude of rainfall (mean rank of 6.7, s.d. = 3.51) and rainfall maximum value (mean rank of 7.89, s.d. = 4.58); the remaining variables referred to wet-day frequency, temperature, humidity, sunshine hours and rainfall again (minimum). Clearly, this list is dominated by rainfall and its variability or duration, and by moisture, indicating dengue's sensitivity to these variables. Spatial predictions in these models were more restricted than in the previous model (cf. figure 1*c* and *b*), following the training set (electronic supplementary material, figure S1d) more accurately (table 1).

As the figures suggest, model accuracy progressively improved with increasing numbers of predictors and according to all accuracy metrics (table 1). Thus, for example, *kappa* increased from 0.31 to 0.46 to 0.72 and AUC increased from 0.74 to 0.8 to 0.91 in the models with one, five or 10 predictors, respectively (standard deviations of all mean values are given in table 1).

### (b) Is the spatial resolution of a climate dataset an important determinant of model accuracy?

**Figure 2.**
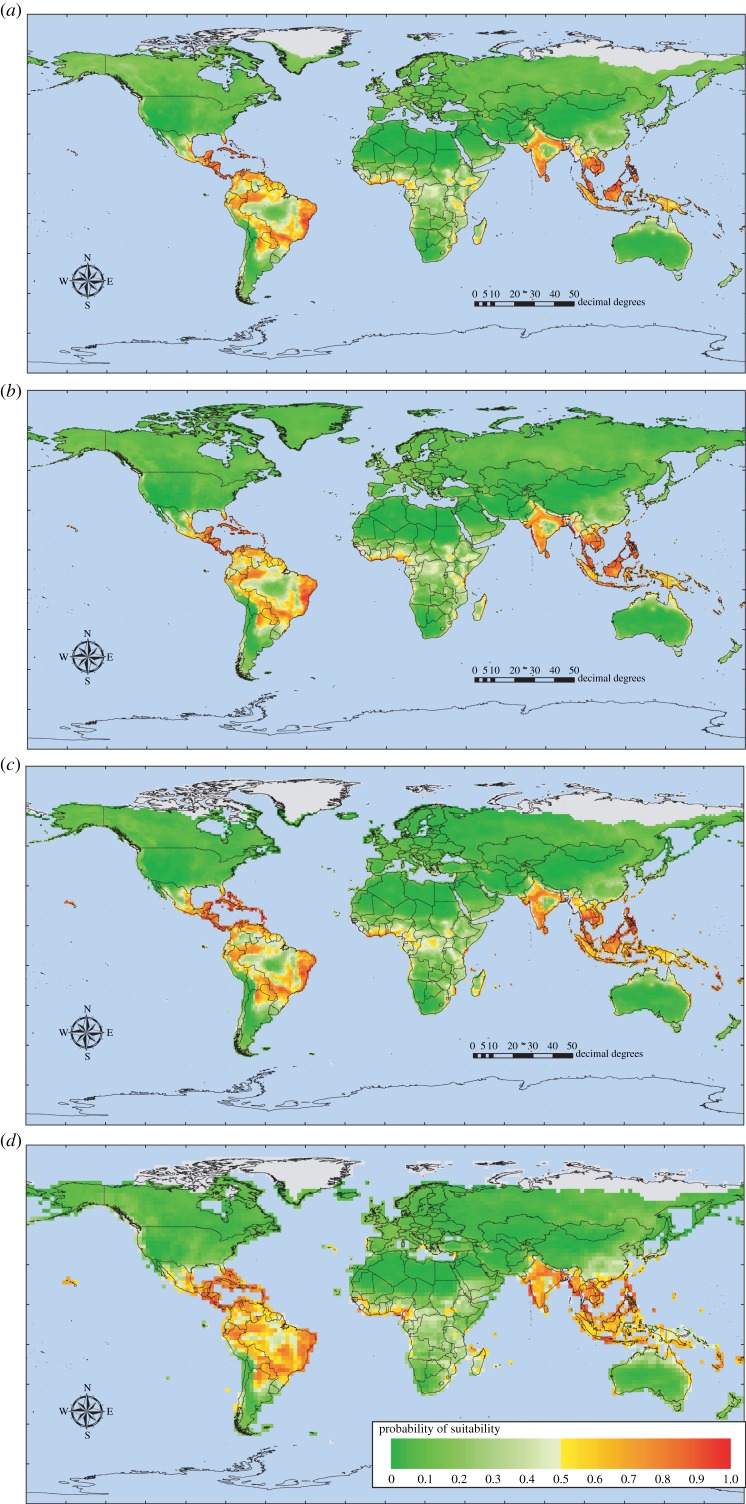
Risk maps for dengue using meteorological data averaged to (*a*) one-third degree, (*b*) one-half degree, (*c*) one degree or (*d*) two degrees. See figure 1*c* for risk map at the original resolution of one-sixth degree and table 2 for accuracy statistics of this series of models.

Figure 2 and table 2 show the risk mapping results when both training set and predictor variable spatial resolutions are progressively down-graded from one-sixth degree (figure 1*c*) to two degrees (figures 2*a*–*d*). Although there are some differences between the averaged final output maps these are not very great, and differences between the accuracy metrics are also very slight (table 2), regardless of whether the models are run at one-sixth or at two degrees of resolution, a 144-fold change in individual pixel area.

**Table 2.** Accuracy statistics for dengue models at different spatial resolutions. Resltn (deg), resolution at which the models were carried out, one-sixth to two degrees. All other symbols are as in table 1.

resltn (deg)	*kappa*	%correct	%PPV	%NPV	%Flse +ves	%Flse −ves	sens.	spec.	TSS	AUC	nvar	*n*
1/6	0.716 (0.029)	85.78 (1.44)	88.74 (2.18)	84.02 (2.50)	8.43 (1.38)	5.78 (1.15)	0.88 (0.023)	0.827 (0.028)	0.707 (0.036)	0.911 (0.014)	9.76 (0.64)	100
2/6	0.719 (0.034)	85.94 (1.72)	88.66 (2.66)	84.76 (2.34)	8.00 (1.40)	6.05 (1.38)	0.874 (0.028)	0.836 (0.029)	0.710 (0.040)	0.911 (0.014)	9.79 (0.62)	100
3/6	0.717 (0.035)	85.87 (1.74)	88.34 (2.43)	84.94 (2.30)	7.84 (1.38)	6.28 (1.48)	0.87 (0.030)	0.838 (0.028)	0.708 (0.041)	0.911 (0.014)	9.8 (0.55)	100
1	0.697 (0.043)	84.86 (2.14)	87.03 (2.21)	84.37 (2.75)	8.26 (1.74)	6.87 (1.25)	0.858 (0.025)	0.83 (0.034)	0.688 (0.042)	0.904 (0.024)	9.69 (0.71)	100
2	0.700 (0.17)	85.00 (8.70)	85.81 (17.93)	86.85 (3.71)	7.00 (2.12)	7.99 (10.04)	0.838 (0.200)	0.855 (0.043)	0.693 (0.205)	0.889 (0.103)	9.68 (0.63)	100

There was also consistency in the variables selected across this range of resolutions. Thus, for example, the annual amplitude of rainfall had the highest mean rank (of between 1.4, s.d. = 1.97, and 4.0, s.d. = 4.26) at all resolutions and bi-annual rainfall amplitude and rainfall maximum were selected in second and third places in the models at the three highest resolutions; mean temperature and mean rainfall were in these positions at one degree resolution and annual variance of rainfall and bi-annual amplitude of rainfall in the same positions in the two degree resolution models. In all cases, the second and third most important variables had considerably lower mean ranks than the first selected variable (6.7, s.d. = 3.51, to 7.9, s.d. = 4.04, for the second variable and 7.9, s.d. = 4.58, to 8.8, s.d. = 4.16, for the third).

### (c) Does inclusion of the future distributions of vectors affect predictions of the futures of the diseases they transmit?

**Figure 3.**
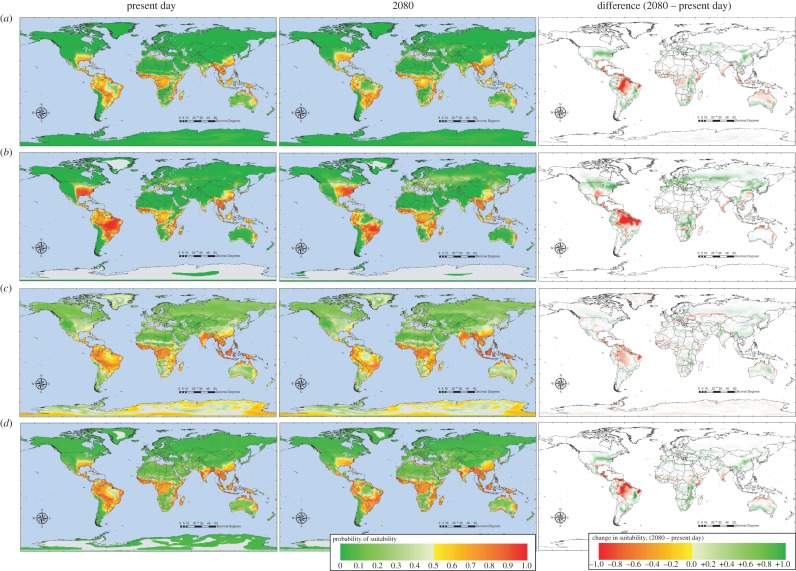
HadCM3 B1a low emissions scenario risk maps for (*a*) *Aedes aegypti*, (*b*) *Aedes albopictus*, (*c*) dengue without vectors and (*d*) dengue with vectors for the present day (left hand column) and for the year 2080 (middle column). The right hand column shows the difference between maps for the present day and 2080 = (2080–present day); in these images red indicates areas of decreased suitability and green areas of increased suitability between these two periods. Not all increases in areas of absence will result in the disease newly appearing in such areas, and not all decreases will result in the disease disappearing; the threshold probability of 0.5 must be crossed for these events to happen.

**Figure 4.**
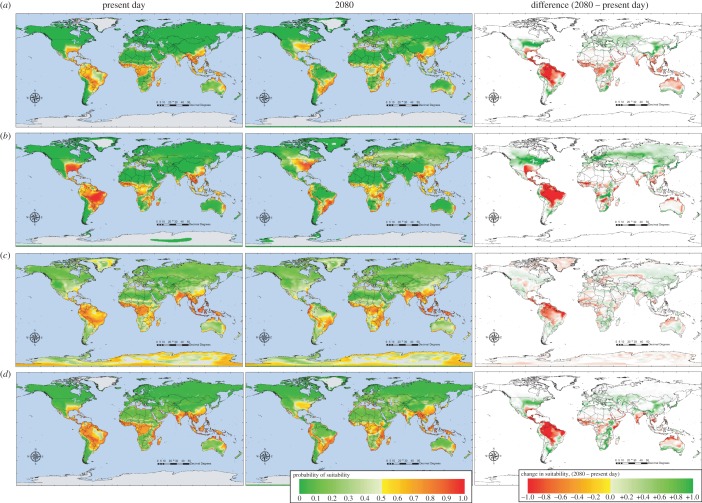
HadCM3 A1F high emissions scenario risk maps. All other details as for figure 3.

Figure 3 shows series of model predictions for *Ae. aegypti*, *Ae. albopictus* and for dengue with and without these two mosquito layers as predictor variables, using GCM data scenario B1a for the present day, and for the year 2080 (the electronic supplementary material gives images for the intermediate years 2020 and 2040), and for the difference between these two time points (2080−present day predictions). Figure 4 shows the equivalent predictions under the high emission A1F1 scenario and table 3 gives accuracy statistics for both sets of models.

**Table 3.** Accuracy statistics for dengue models using the HadCM3 climate data. B1a and A1F are alternative low and high emissions scenarios of the HadCM3 series of GCMs. Symbols are as in table 1. Results refer to the climate of the 1980s as modelled by the two scenarios, which are not very different at this point in time. NA, not applicable.

	*kappa*	%correct	%PPV	%NPV	%Flse +ves	%Flse −ves	sens.	spec.	TSS	AUC	AICc	nvar	*n*
B1a scenario													
*Aedes aegypti*	0.475 (0.052)	73.75 (2.60)	74.83 (3.37)	74.49 (3.32)	13.10 (1.82)	13.14 (1.81)	0.734 (0.034)	0.733 (0.037)	0.467 (0.050)	0.806 (0.028)	NA	4.85 (1.14)	100
*Aedes albopictus*	0.646 (0.051)	82.31 (2.58)	90.14 (2.96)	76.33 (5.97)	12.80 (3.19)	4.88 (1.51)	0.897 (0.031)	0.74 (0.063)	0.638 (0.070)	0.896 (0.030)	NA	5.65 (2.26)	100
dengue without vectors	0.397 (0.063)	69.83 (3.17)	84.70 (3.64)	56.77 (4.23)	22.09 (2.40)	8.07 (2.37)	0.834 (0.048)	0.555 (0.047)	0.389 (0.067)	0.757 (0.039)	724.5 (22.9)	3.5 (0.92)	100
dengue with vectors	0.427 (0.050)	71.32 (2.50)	82.32 (3.07)	61.94 (3.66)	19.61 (1.87)	9.06 (1.59)	0.814 (0.032)	0.605 (0.036)	0.419 (0.048)	0.771 (0.029)	703.0 (22.1)	3.27 (1.24)	100
A1F scenario													
*Aedes aegypti*	0.47 (0.05)	73.49 (2.50)	75.12 (4.11)	73.63 (2.65)	13.47 (1.50)	13.03 (2.08)	0.737 (0.041)	0.726 (0.031)	0.463 (0.051)	0.804 (0.026)	NA	4.64 (1.11)	100
*Aedes albopictus*	0.657 (0.053)	82.84 (2.65)	89.76 (2.44)	77.84 (5.82)	12.26 (3.44)	4.89 (1.34)	0.897 (0.028)	0.751 (0.070)	0.648 (0.075)	0.897 (0.033)	NA	5.8 (2.40)	100
dengue without vectors	0.389 (0.072)	69.43 (3.59)	84.38 (4.57)	56.88 (4.81)	22.28 (2.79)	8.28 (2.47)	0.829 (0.049)	0.551 (0.056)	0.380 (0.074)	0.755 (0.039)	724.8 (22.8)	3.49 (1.07)	100
dengue with vectors	0.443 (0.054)	72.15 (2.67)	83.96 (3.04)	62.40 (3.88)	19.46 (2.16)	8.37 (1.65)	0.827 (0.033)	0.608 (0.043)	0.435 (0.054)	0.78 (0.030)	696.3 (23.8)	3.37 (1.39)	100

Under present-day climate scenarios, mean temperature and relative humidity minimum variables were in the top two positions for the 100 bootstrap models for *Ae. aegypti* under both B1a (mean ranks for these two variables of 1.62, s.d. = 1.54, and 2.39, s.d. = 2.42, respectively) and A1F (mean ranks of 1.36, s.d. = 1.15, and 2.69, s.d. = 2.65, respectively) scenarios, and they were both selected in more than 90 models in each series. Similarly, relative humidity mean and temperature maximum were selected as the top two variables in more than 90 of the *Ae. albopictus* models (mean ranks of 2.11, s.d. = 2.92, and 2.24, s.d. = 1.19; and 1.78, s.d. = 2.59, and 2.4, s.d. = 1.78, in the B1a and A1F models, respectively). Average *kappa* and AUC values indicated a rather better fit for *Ae. albopictus* (*kappa* = 0.646, AUC = 0.896 in B1a and *kappa* = 0.657, AUC = 0.897 in A1F) than for *Ae. aegypti* (*kappa* = 0.475, AUC = 0.806 in B1a and *kappa* = 0.47, AUC = 0.804 in A1F; further details, with s.d. values, in table 3). Thus, both mosquito vector species' distributions depend on temperature and relative humidity. Maximum precipitation was the most important rainfall variable for *Ae. aegypti* in the B1a scenario, but had a low mean rank of 7.07, s.d. = 3.19, and mean precipitation the highest rainfall variable in the A1F scenario (again with a low mean rank of 7.67, s.d. = 3.48). For *Ae. albopictus*, maximum precipitation was also the most important rainfall variable under both scenarios, again with a low mean rank (6.14, s.d. 3.21, in B1a and 6.09, s.d. = 2.97, in A1F). These low average ranks for rainfall for both mosquito species' models indicate that this is not a critical variable for these two species.

For the dengue models without vectors, precipitation maximum and relative humidity minimum were selected in more than 80 of the B1a scenario models (mean ranks of 1.9, s.d. = 2.88, and 4.15, s.d. = 3.41, respectively; mean *kappa* = 0.397, AUC = 0.757) and again in more than 65 of the A1F scenario models (mean ranks of 2.1, s.d. = 3.14, and 5.19, s.d. = 4.04, respectively; mean *kappa* = 0.389, AUC = 0.755). These accuracy statistics are considerably lower than those from the meteorological records based models (table 2), but it should be remembered that a very reduced variable dataset was available from the GCM-based models, from which often no more than three to six variables were selected to provide an optimum fit for the bootstrapped samples (table 3, nvar column). A better comparison is therefore with the five-variable model in table 1. In these dengue models (i.e. without vectors), minimum temperature was the most important temperature variable in both scenarios, with a low mean rank of 8.43, s.d. = 3.87, and 8.58, s.d. = 3.79, in the B1a and A1F scenarios, respectively.

Addition of the two mosquito vector layers resulted in *Ae. aegypti* being selected both in the B1a models, along with precipitation maximum and relative humidity minimum in more than 70 models (mean ranks of 1.1, s.d. = 1.00, 4.41, s.d. = 3.40, and 4.51, s.d. = 3.98, respectively; mean *kappa* = 0.427, AUC = 0.771) and in the A1F models along with relative humidity minimum and precipitation maximum, again in more than 70 models (mean ranks of 1.22, s.d. = 1.42, 4.74, s.d. = 4.13, and 5.16, s.d. = 3.82, respectively; mean *kappa* = 0.443, AUC = 0.780). The mean ranks of the *Ae. albopictus* layer in the dengue models were low, 9.23, s.d. = 3.13, in the B1a scenario and 8.69, s.d. = 3.52, in the A1F scenario, indicating a much less important role for this vector in predicting dengue globally. Minimum temperature (the highest ranking thermal variable) had an even lower mean rank in these models, of 10.3, s.d. = 2.15, in the B1a scenario and 9.76, s.d. = 2.75, in the A1F scenario Thus, dengue's distribution is best described by its main vector's distribution along with rainfall and relative humidity; temperature is much less important.

Given that the dengue models without vectors depend mostly on precipitation and humidity but in the presence of vectors depend rather heavily on *Ae. aegypti*, we might expect a difference in the predictions of dengue's future distribution under both emissions scenarios when the mosquito predictor layers are available to the models compared with when they are not. This is shown in figures 3 and 4. The following conclusions are drawn mostly from the high emission A1F scenario models (figure 4), but the same, less pronounced trends can also be seen in the lower emissions scenario models (figure 3). Dengue models without vectors show a contraction of dengue's distribution in some areas in the future (e.g. the Amazon basin in South America) and an expansion in others (e.g. along the southeast coast of Africa, and into mainland China). Dengue models with vectors show many of the same trends but these are more pronounced in most areas (e.g. the contraction in the Amazon and expansion in China) and, in addition, new areas are deemed to become suitable (e.g. the eastern USA and the east coast of central South America). There is also a marked change towards increasing suitability in a band running from Europe eastwards, probably driven by the increasing suitability in these same areas for the two vector species (the green areas in figure 3, right-hand panels). In many of these areas, however, suitability does not increase enough to give a prediction of presence, either for the vectors or the disease.

### (d) Which are the key climate variables involved in determining changes in the distributions of vector-borne diseases in the future?

**Figure 5.**
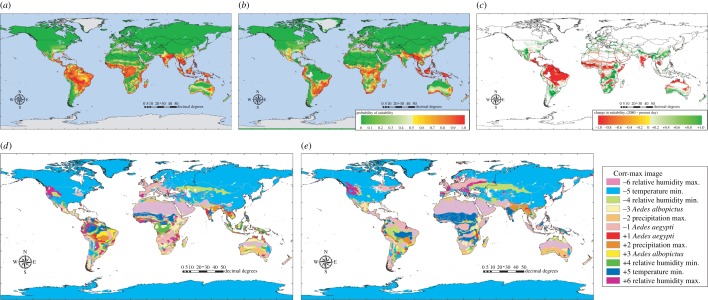
HadCM3 A1F emissions scenario dengue risk maps using a reduced set of predictor variables including the two mosquito vector maps for (*a*) the present day and (*b*) the year 2080. (*c*) A difference image between (*a*) and (*b*)=(2080–present day). (*d,e*) Corr-max images derived from the models shown in (*a*) and (*b*) where each colour represents the variable (legend above (*d*)) making the greatest contribution to the MD to the nearest presence cluster (see text for details).

To illustrate the Garthwaite–Koch method proposed in Material and methods (§2) to identify key variables in the sorts of predictions presented in §3c, a version of the A1F scenario dengue model was run again, this time as a single model using all the presence points and an equal number of pseudo-absence points. Initial inspection resulted in the removal of some highly correlated environmental variables, leaving the following variables in the model: both of the mosquito vector species, precipitation maximum, temperature minimum and relative humidity maximum and minimum. This model was run under present-day conditions and then under the predicted future conditions of the 2080s. The results are shown in figure 5. The first thing to note in figure 5 is that the predictions of dengue's distribution from this single model are very similar to the results of the equivalent bootstrapped series (figure 4), both at the present time and in the 2080s (figures 5*a,b*). Figure 5*c* shows the difference image between figures 5*a* and *b* (for comparison with figure 4*d*) and figure 5*d,e* shows the maps arising from the corr-max procedure (Material and methods, §2) applied to the models of figure 5*a,b*. The colour coding is on a rainbow scale; red, orange, yellow, green, blue and violet corresponding to the six key variables (the order is given in the legend in figure 5), with darker shades of each colour indicating positive values. Thus, for example, the colours red or pink refer to the first variable, *Ae. aegypti.* Pink patches in figure 5*d,e* indicate areas where the absence of *Ae. aegypti* was the most limiting variable in making the risk map predictions, for example in central/southern Africa and in much of western Europe. The blue or pale blue areas in these figures indicate where minimum temperature is limiting. In high northern latitudes, in both North America and Asia, conditions are below the minimum temperature requirements for dengue (indicated by the pale blue colouring), but in the southern Sahara minimum temperature conditions are well above those that are suitable for dengue (indicated by the dark blue colouring).

Perhaps the most striking difference in figure 5 is shown in the Amazon basin of South America, where dengue is predicted present under current conditions but absent by the 2080s. Figure 5*d* shows that quite a number of the input variables contribute most to the MD values at the present time, but all of these are small enough such that the low total MD results in a prediction of dengue presence. By the 2080s, however, when dengue is predicted to be absent from this area, a smaller number of variables are involved; pink, *Ae. aegypti*; pale green, relative humidity minimum; and dark blue, temperature minimum. The first two of these have negative signs: *Ae. aegypti* is absent from these areas by the 2080s and the minimum relative humidity predicted for that period is much lower than that required by dengue. But the third variable has a positive sign, indicating that the 2080s predicted temperature minimum is much higher than dengue requires; in these areas, therefore, by the 2080s, it becomes too dry and too hot for dengue (and its major vector). The electronic supplementary material shows additional versions of figure 5*d,e* split according to predicted presence or absence.

## 4. Discussion and conclusion

The four key questions posed at the start of this article have thrown some light both on the future of dengue globally and, more importantly, on some of the processes and decisions involved in making such predictions for the future.

In a review of the possible impacts of climate change, the United States National Research Council included the following statement about modelling the future of diseases of various types:

In general, the simpler a model is, the better we understand its implications. The assumption that more complex models will more closely approximate reality is not justified in cases where there is a paucity of data on relevant ‘measurables’ [54, p. 64].

This statement is clearly a plea for parsimony in model building although it has also been taken to justify the use of only a single predictor variable in a widely quoted model of dengue futures [18]. Figure 1 shows that it is unlikely that a single variable can capture the complexities of the distribution of any disease, let alone a vector-borne one, and that adding more variables tends to increase the accuracy of the models. The question then becomes ‘How many is too many?’ In statistical models, new variables should only be added if they increase the fit of the models, best measured using information theoretic criteria [42]. In biological or process-based models, simply choosing the ‘obvious’ variables such as temperature and rainfall runs the danger of omitting less obvious variables which may be equally important in determining species' distributions; for such models the obvious question might be ‘How few is too few?’.

Parsimony, which is intrinsically good for the reasons outlined in the NRC quote, is in fact forced upon the present work using statistical models because of the rather limited number of climate variables available from GCMs. The variables available, however, may not be the right ones for the disease in question. In addition, as the present analysis has shown, some of the available GCM variables are very highly correlated with each other, reducing the number of useful variables still further. Statistical modellers predicting the future of diseases might therefore re-phrase St Augustine's plea to the Almighty (about remaining chaste) in the following terms: ‘Make us parsimonious, but not yet’.

Another problem with GCMs is their intrinsically poor spatial resolution. Initial GCM models had resolutions of more than two degrees of both latitude and longitude. Model resolution has improved dramatically in recent years (currently to about half of one degree for global models) but this is still too coarse to predict fine details of any species' distribution. What was surprising in this study was that predictive model accuracy remained high from the original spatial resolution of the meteorological dataset of one-sixth of a degree right up to the sort of spatial resolution of GCMs (two degrees, figure 2), something already noted more generally in species' distribution modelling when the resolution of the observations on species distributions are of a coarser grain size than the finest resolution of the predictor variables [37]. The present exercise may, however, give a false impression about the (?in)significance of spatial resolution in all situations, because the original meteorological data used here were of relatively fine resolution, and the averaging process involved in making the larger pixels would have incorporated some of this detail in the overall average. In contrast, GCM model predictions are made for points on a coarse global grid. The half-way line between grid points determines the size of the pixels to which that single grid value is applied; conditions across the pixel are therefore considered uniform. Any scaling of the results to a finer spatial resolution (for example as here, using cubic spline interpolation) is making some fairly strong assumptions about the nature of the variation between grid points, which is assumed to be smooth and regular. This is unlikely to apply universally, or even at all. Thus, one should not expect to retain the same degree of model accuracy across a range of down-scaled GCM data as is maintained across a range of up-scaled meteorological data.

GCM models, although improving all the time, still cannot mimic observed climates with complete accuracy. Therefore, application of GCMs to models involving climate-sensitive insect vectors should not be expected to give the same sorts of accuracy as application of equivalent observed meteorological data. A comparison of more or less equivalent models of dengue produced using either observed meteorological data (e.g. the five variable model of table 1) or GCM predictions for the present day (e.g. either scenario ‘Dengue without vectors’ models in table 5) shows a greater accuracy of the former (*kappa* = 0.46, AUC = 0.8 versus *kappa* = 0.40, AUC = 0.76 for the B1a scenario), but the differences are not huge. What is clearly required, therefore, is the output of a greater variety of meteorological variables from GCM models, so that more can be used in disease distribution models of the future.

The role of vectors in vector-borne diseases is obviously crucial. In the *R*_0_ biological model for a standard vector-borne disease such as malaria, six of the seven key parameters and variables are related to vectors, their abundance, mortality or susceptibility to disease [31,55]. Thus, it should not be surprising that vector distributions, when available, are often selected even in statistical models for vector-borne diseases. There is clearly an effect of vectors in the models described here (contrast, for example, figures 3 and 4, rows (*c*) and (*d*)) and this effect is likely to have been underestimated because of the limited variety of climate predictors available for both vectors and disease. Among this limited set, temperature seems to be more important in determining vector distributions than dengue distribution on its own, and rainfall is much more important for the disease on its own (i.e. in statistical models without the vectors). This difference in importance may help to explain why dengue models with the mosquito vector layers gave different predictions from those without (the usefulness of vector layers in the former is confirmed by the fact that they are often selected as the most important predictor variable). Nevertheless, a wider range and type of predictor variable might highlight even more the different climatic requirements of dengue and its vectors, and therefore the difference between dengue models with and without the mosquito distributions as predictor variables. It could, of course, be claimed that both sorts of model are intrinsically statistical and therefore that the dengue models without the mosquito vectors will find some proxy among the available climatic variables to replace the vector layers as useful predictors. If this were the case, however, we might expect the dengue models without vectors to select, as a proxy for the vectors themselves, the same sorts of climate variables that are important in determining the vectors' distribution. The models here did not seem to be doing this, though whether this is because the vectors have a much more widespread distribution than does the disease (mosquito distributions extend into colder areas, where temperature conditions are more likely to be limiting, and therefore temperature variables are more likely to be required to model them) or because of intrinsic differences between dengue and its vectors within the areas where both occur, remains to be demonstrated.

As pointed out in §1, many other factors (landscape, environmental, socio-ecological) affect the current distribution of dengue globally and are likely to operate in the future. Statistical models using only climate variables to define dengue's realized niche may find among the climate dataset proxies for those non-climatic constraints (i.e. climate variables correlated with important landscape or other variables) which may be used in their place, but this is clearly not ideal. The challenge now is to discover how the selected climate variables operate on the dengue system, and to discover new, non-climate variables that increase model accuracy still further.

Identifying the key model variables involved in predicting the changing spatial pattern of vector-borne diseases over time is now made possible by the Garthwaite–Koch technique, used here for the first time in predictive risk mapping, of corr-max transformation (figure 5), and calculating weightings of the input variables on the Garthwaite–Koch scale. There are a number of subtleties in this technique that make it promising for future use. For example, it is easy to calculate variance inflation factors that identify which correlated variables need to be rotated in order to reduce the correlation between them [49]. When correlated variables are rotated, the results for all the other variables remain exactly the same. Maps such as those shown in figure 5 are useful to demonstrate that a variety of predictor variables may be limiting the disease, even across areas where it is uniformly present or absent. Figure 5 identifies only the most important variable determining the size of the MD (the corr-max technique itself is able to rank and quantify all variables with respect to their importance); the second and subsequent variables may be almost as important, or of much lesser importance, and so figure 5 should be taken only as a guide to aid understanding of why diseases may expand or contract over time. Within a geographic information system, it is possible to use a multilayered version of figure 5*d,e*, so that the Garthwaite–Koch weightings of all the variables can be examined simultaneously.

Predictions are often made for the future of vectors or diseases on the basis of a variety of statistical, mechanistic or process-based models, and the results are often believed before they are properly understood. As pointed out in §1, predictions that cannot be disproved are unscientific and take us almost into the realm of alchemy. Isaac Newton, one of the first great scientists, was also called the last alchemist. For men of his time, the idea that white light could be turned into rainbow colours on passing through a prism was just about as likely as it was that base metal could be turned into gold, or that a panacea existed for all diseases. We now distinguish among this multitude of possibilities by using the scientific method to disprove things that cannot possibly be true and to fail to disprove other things that might possibly be true [1]. The possibility of disproof is therefore critical in distinguishing fact from fiction, or science from non-science. In the field of predictions about the future, disproof at the present time is clearly impossible. We must therefore be especially careful with such predictions, and with any recommendations for action that might arise from them.

Instead of believing model predictions for the future before understanding them we should seek the more defensible goal of understanding them in order to believe. Understanding why models for the future behave in the ways that they do will allow us to check this understanding against not only our current knowledge of vector-borne disease epidemiology, but also our current experience of the manifold ways in which key elements in transmission pathways can change, sometimes rapidly, to alter the nature or extent of the diseases at the present time. Diseases can adapt to new vectors in different places, or can change rapidly through the spread of mutations, making them more infectible to the vectors they already have. How both vectors and diseases might adapt their thermal tolerances as climates change is relatively unknown. As has been pointed out elsewhere, vector-borne diseases involve a number of factors, some of which will increase transmission as temperatures rise (e.g. decreasing intrinsic incubation periods in the vectors) and some of which will decrease it (e.g. increasing vector mortality rates) [55]. The balance that is struck between these will vary from one disease to another, from one place to another and also from one time to another.

We do not have to apply the equivalent of Pauli's ‘desperate remedy’ to the problem of predicting the future of vector-borne diseases (i.e. to suggest the existence of something that cannot be proven) but we must be much more circumspect in the claims we make for any prediction of disease futures.

## Supplementary Material

Supplementary material
