# Supplementary material for: Dengue: recent past and future threats
Source: Philos Trans R Soc Lond B Biol Sci. 2015 Apr 5;370(1665):20130562. doi: 10.1098/rstb.2013.0562 (PMC4342966; doi:10.1098/rstb.2013.0562)
Supplement: Supplementary material [file rstb20130562supp1.pdf]

David J.Rogers: 'Dengue: recent past and future threats' doi: 10.1098/rstb.2013.0562

ELECTRONIC SUPPLEMENTARY MATERIAL

This material is in A3 landscape format and provides larger versions of the text figures, plus supplementary figures referred to in the text

Figure 1a. Risk map for dengue using one predictor variable (Relative Humidity). Resolution 1/6th degree.

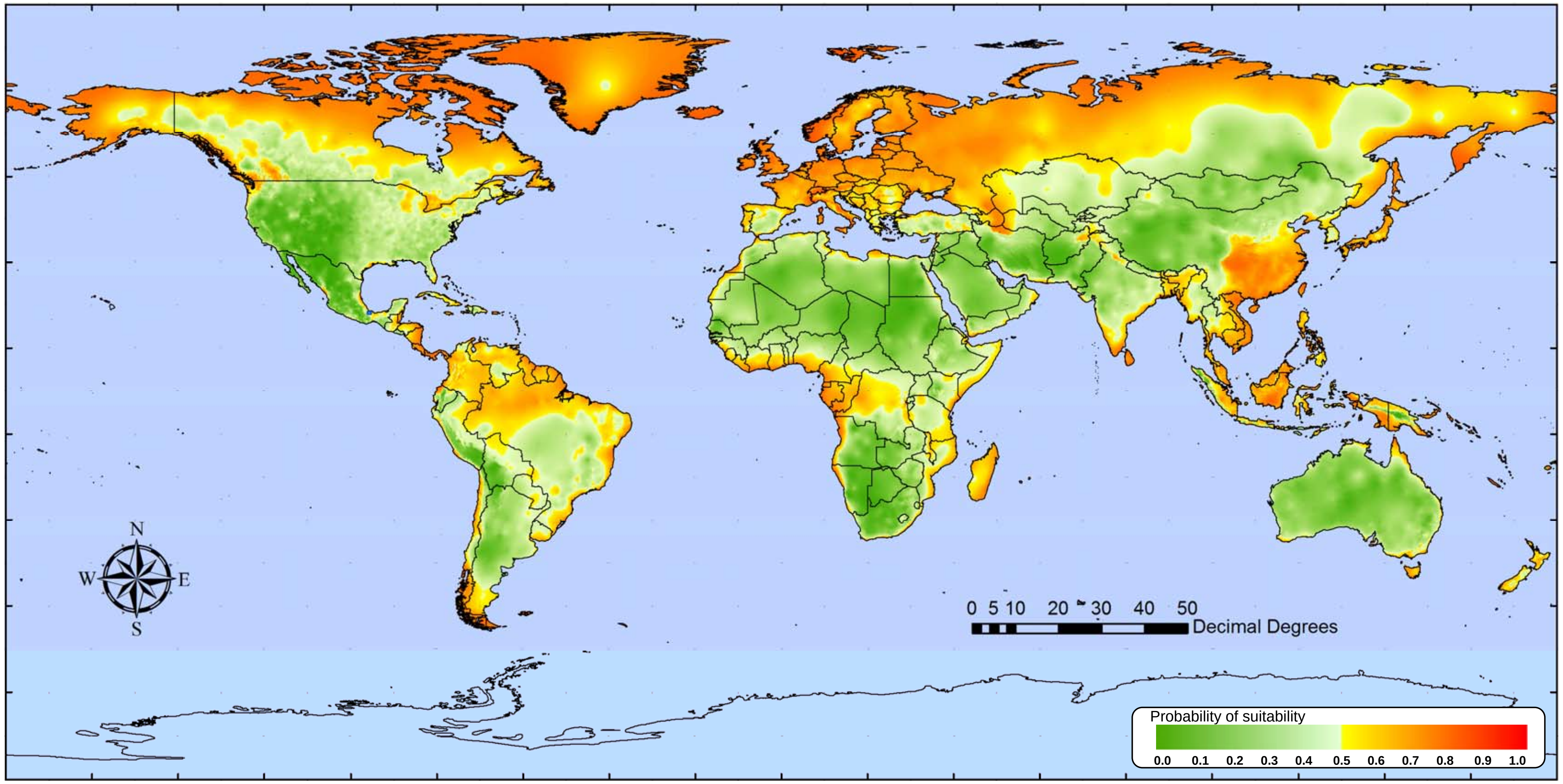

Figure 1b. Risk map for dengue using five predictor variables. Resolution 1/6th degree.

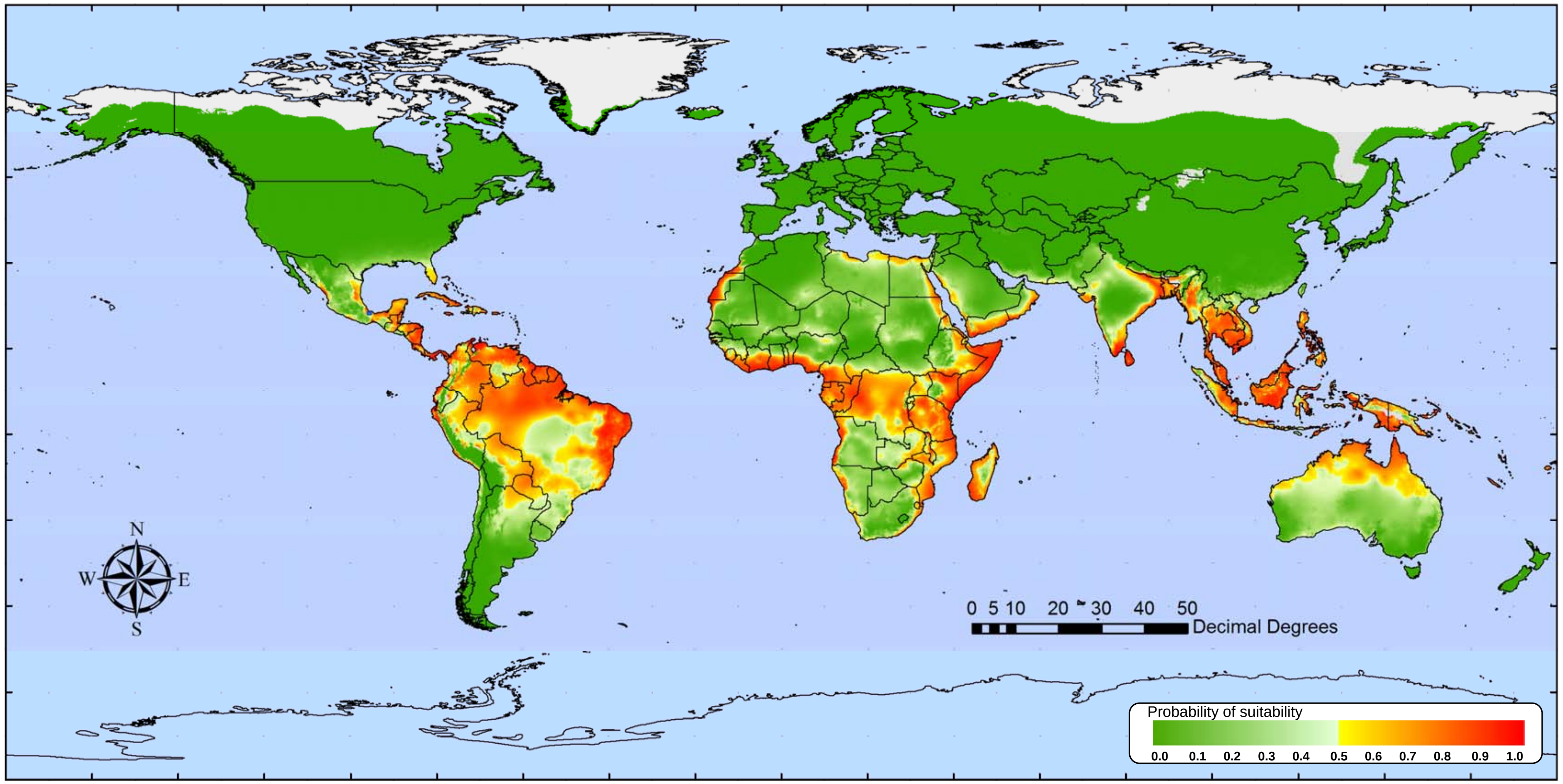

Figure 1c. Risk map for dengue using 10 predictor variables (selected from 70 Fourier processed meteorological variables). Resolution 1/6th degree.

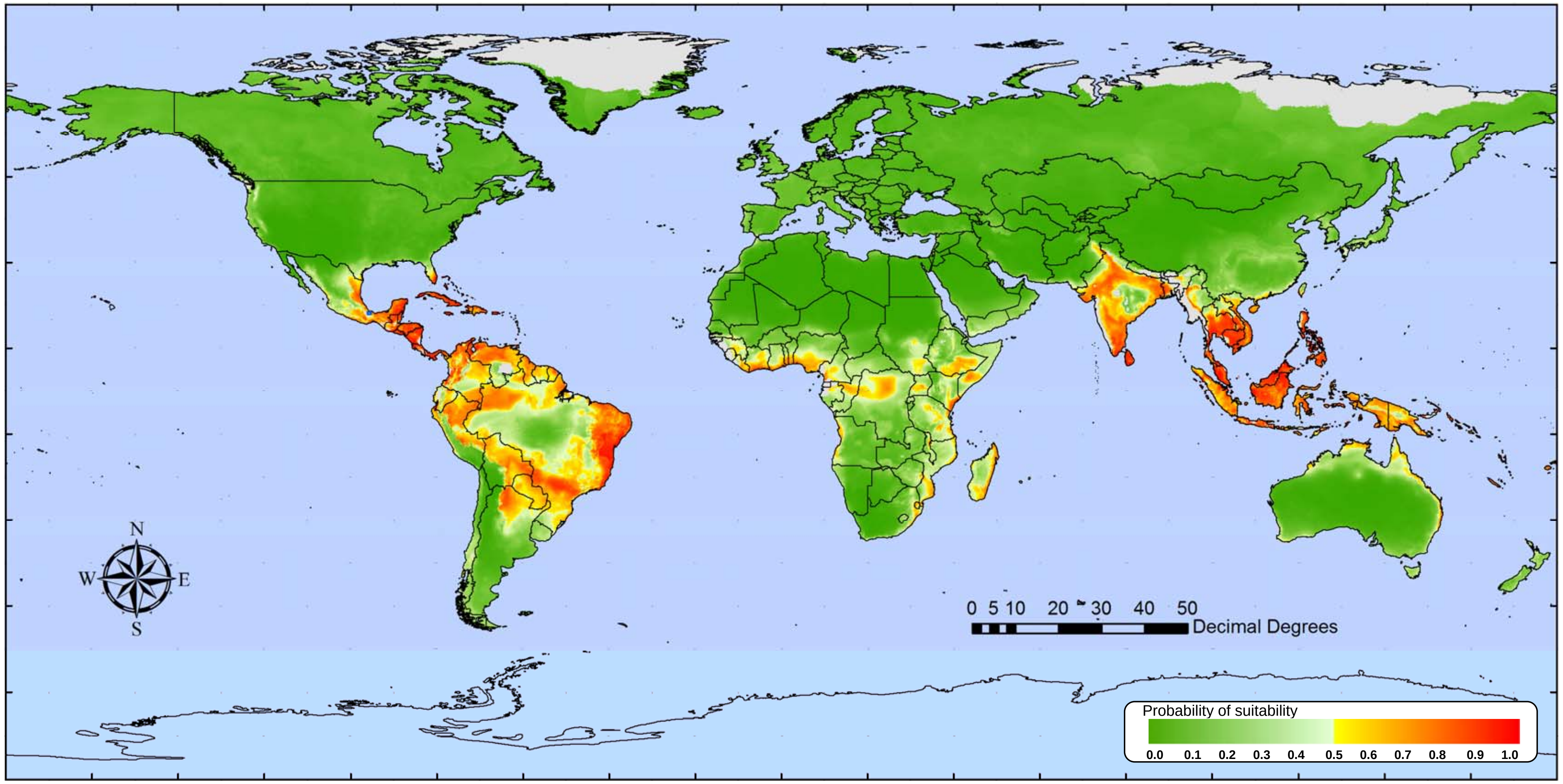

Figure 1d with dengue points. As figure 1c but with the dengue presence points from the training set (black dots)

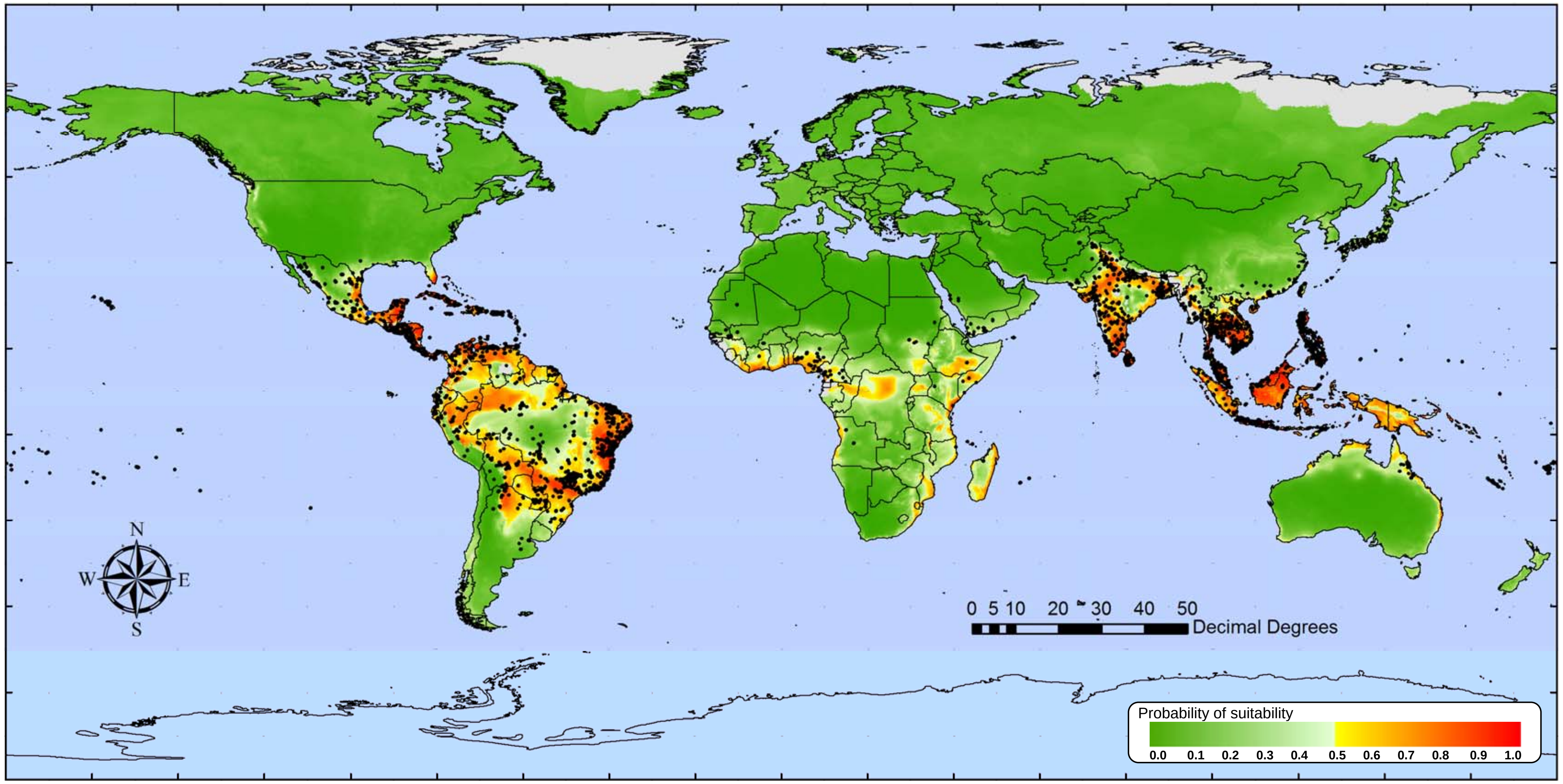

Figure 1e. Dengue presence points in the training set.

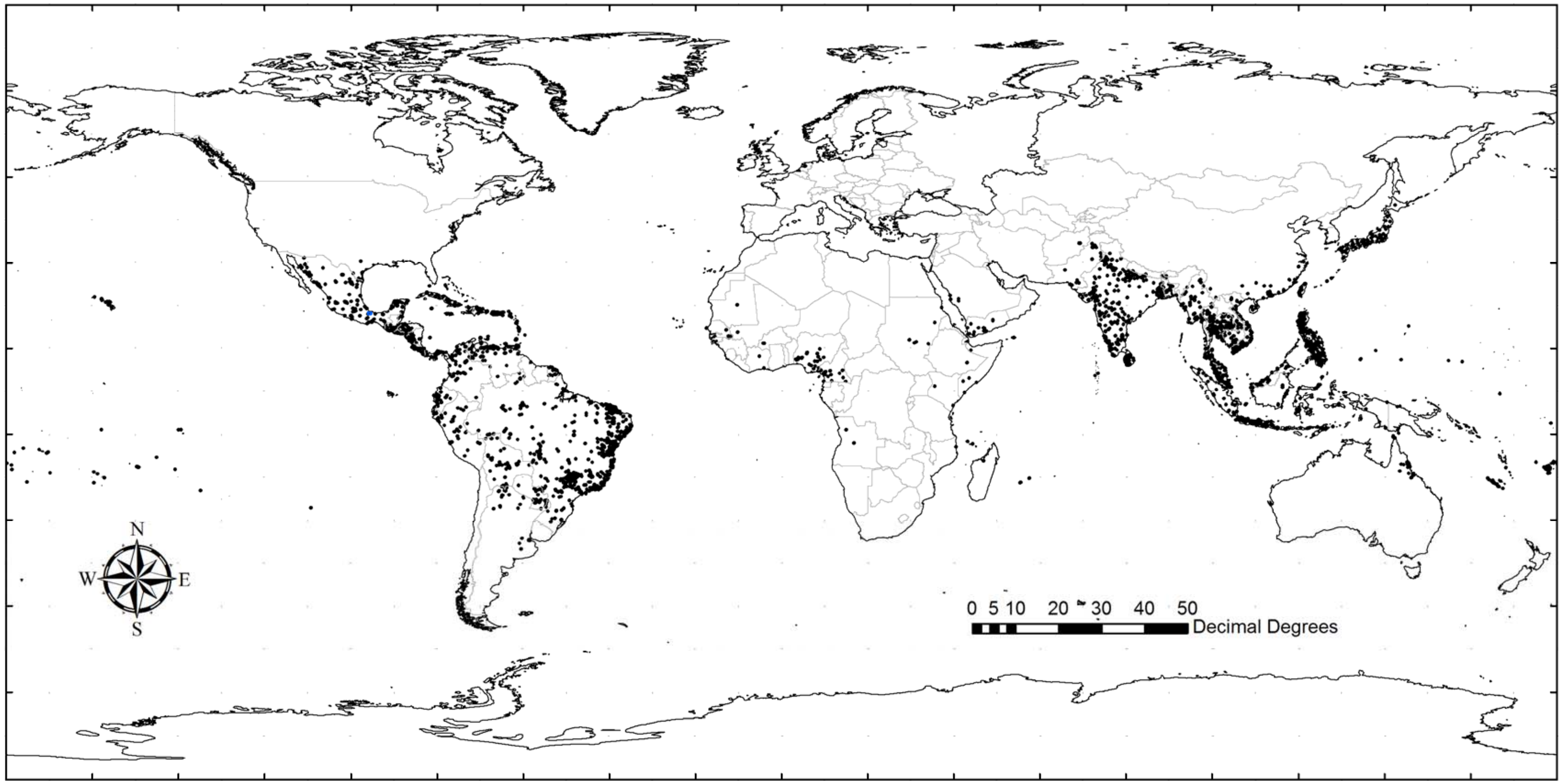

Figure 1f. *Aedes aegypti* presence points in the training set.

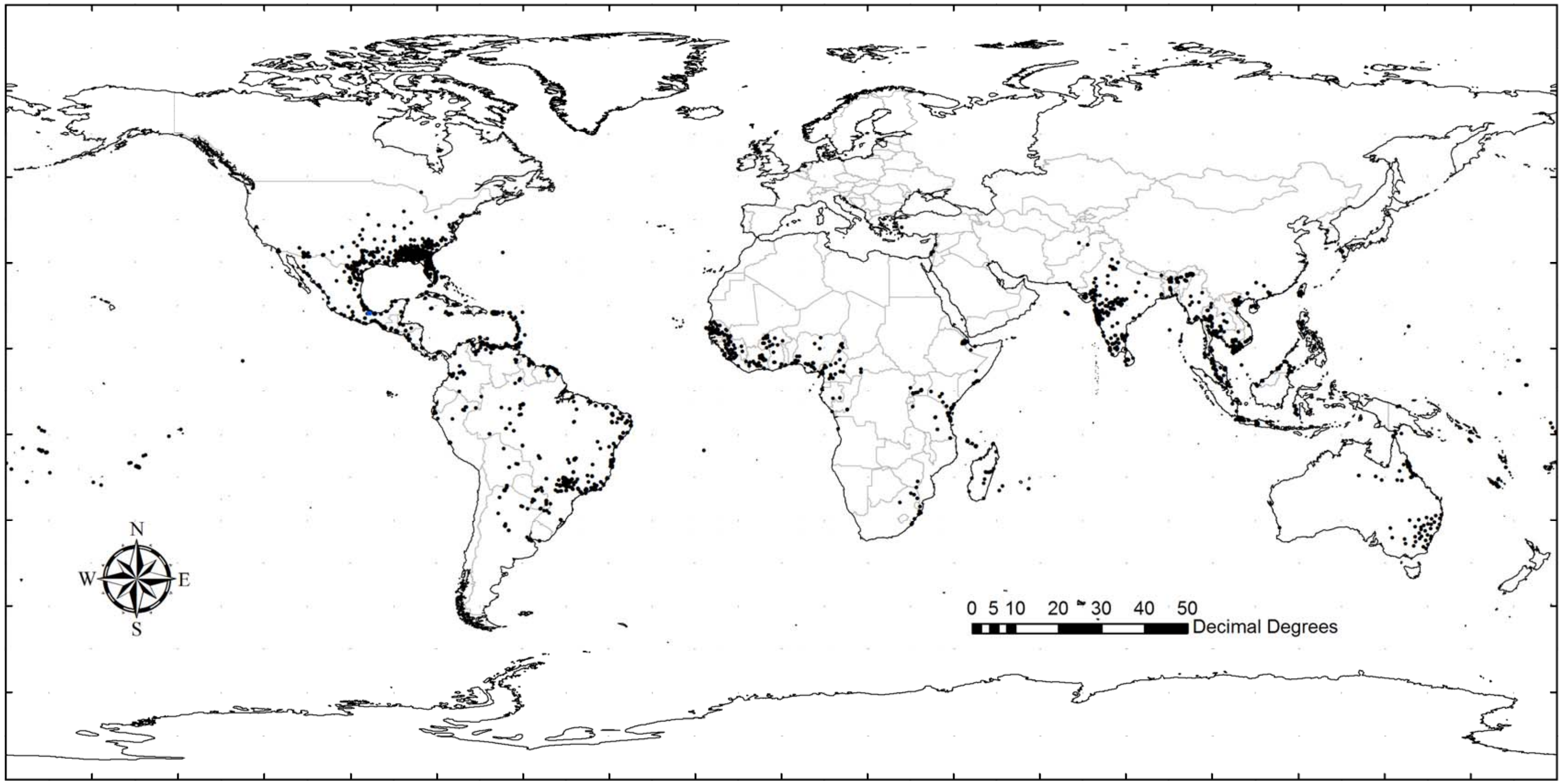

Figure 1g. *Aedes albopictus* presence points in the training set.

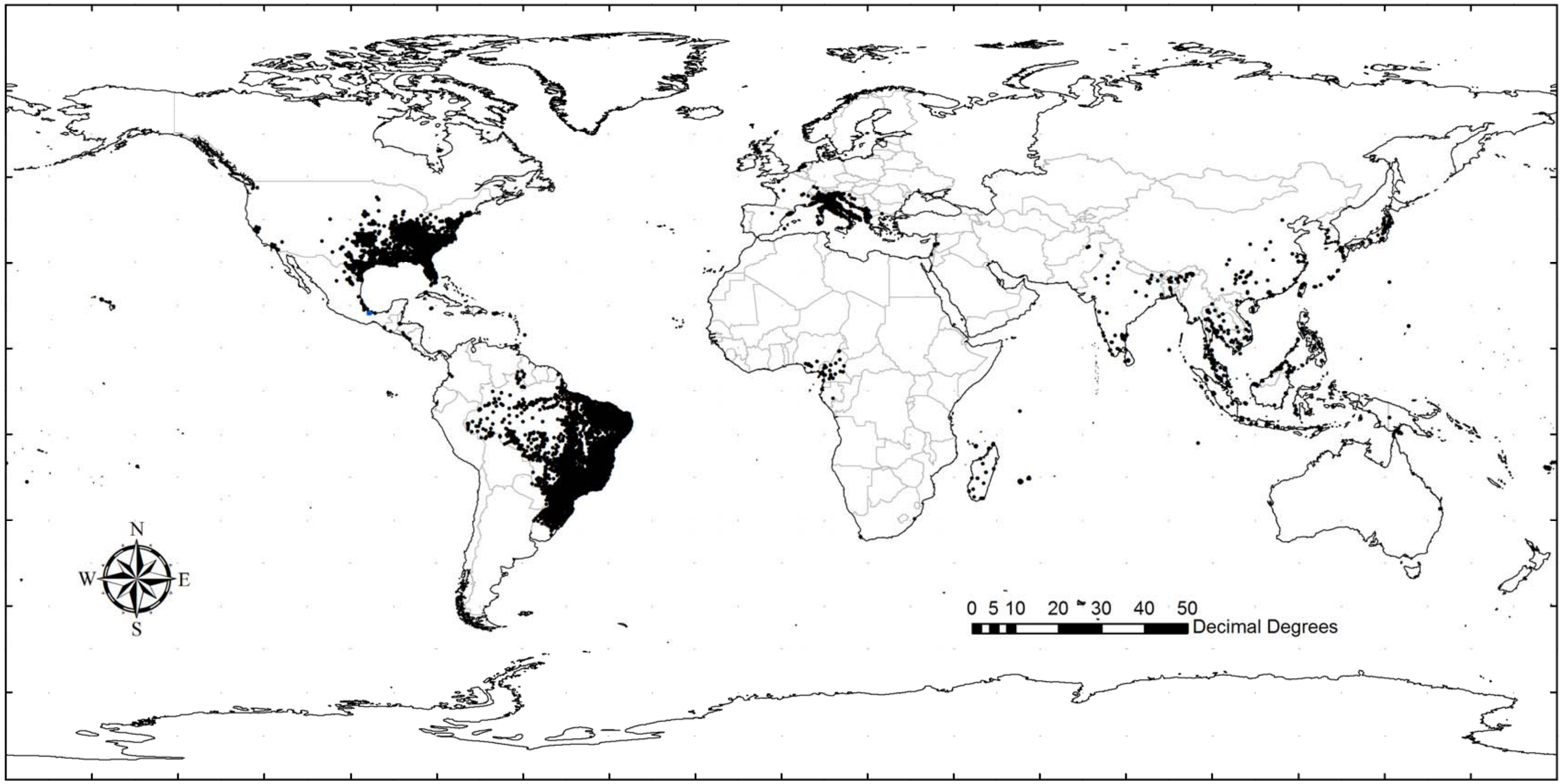

Figure 1h. Mean latitudes of all the dengue records in the database by decades from 1960-1969 to 2000-2010. Red, Northern hemisphere; blue, southern hemisphere; grey, mean absolute latitude (both hemispheres). Yellow point = mean absolute latitude of all records until 1990. Green point = mean absolute latitude for all records after 1991. Error bars are standard deviations (above each point) or standard errors (below each point): all on left y-axis. Number of records by decade for the Northern (red stippled line) and Southern (blue stippled line) hemispheres: right y-axis.

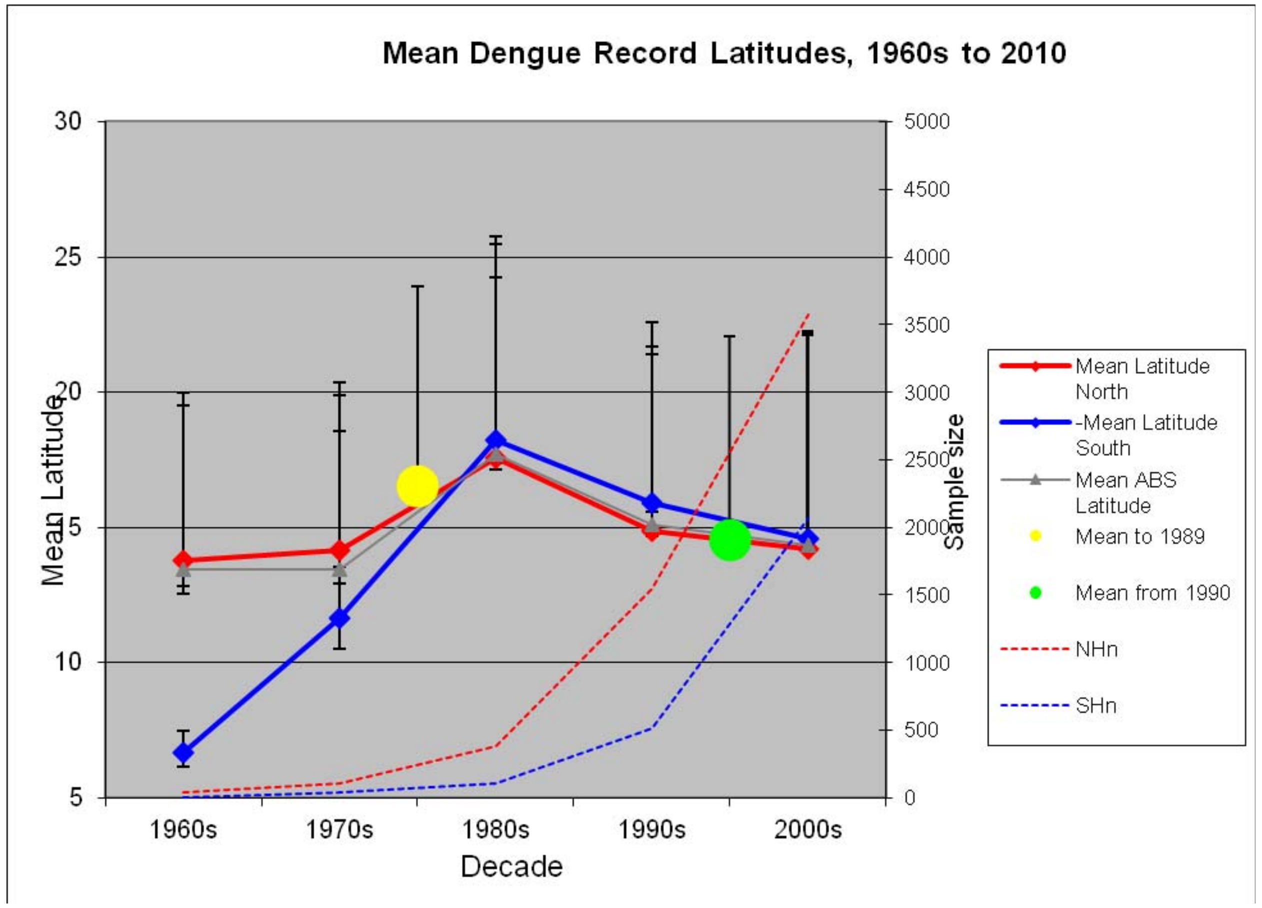

Figure 1i. As Fig. 1g, but for the *Aedes aegypti* records in the database.

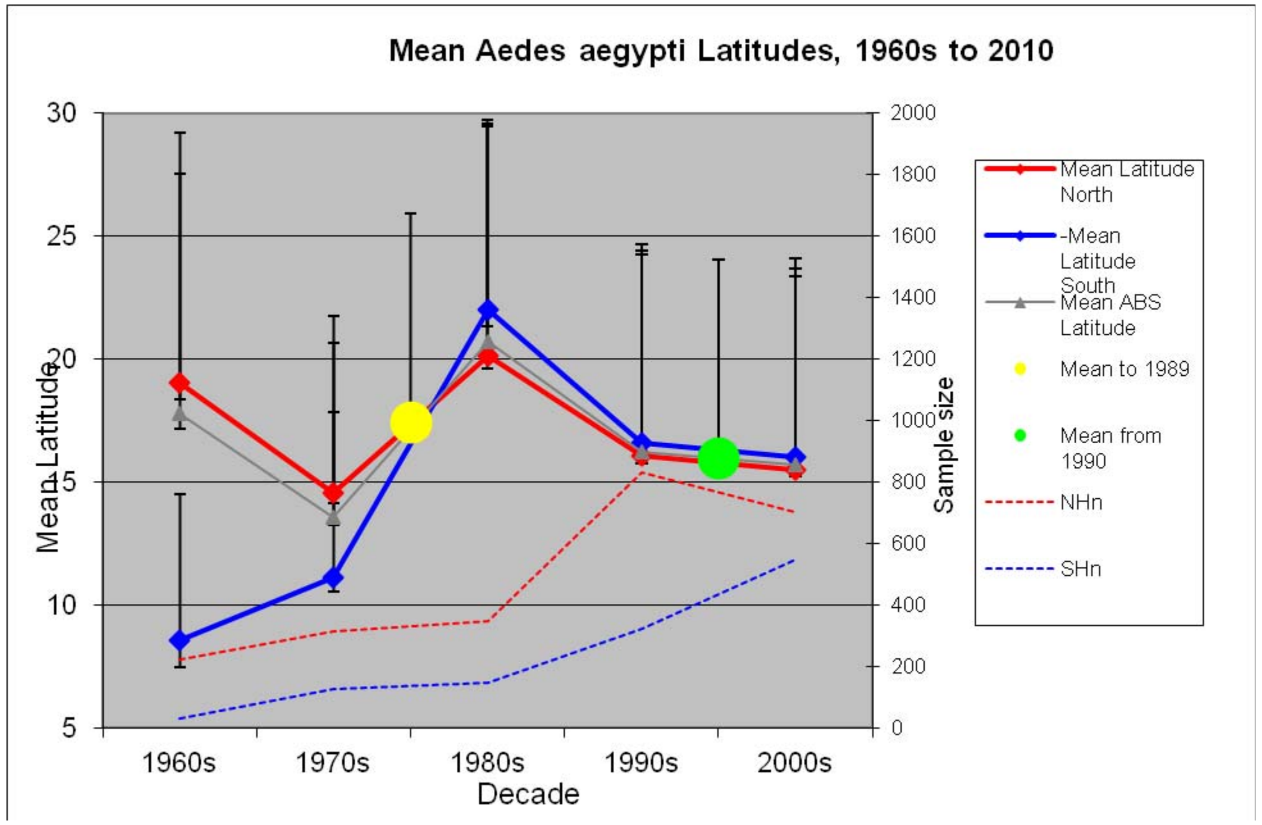

Figure 1j. As Fig. 1g, but for the *Aedes albopictus* records in the database.

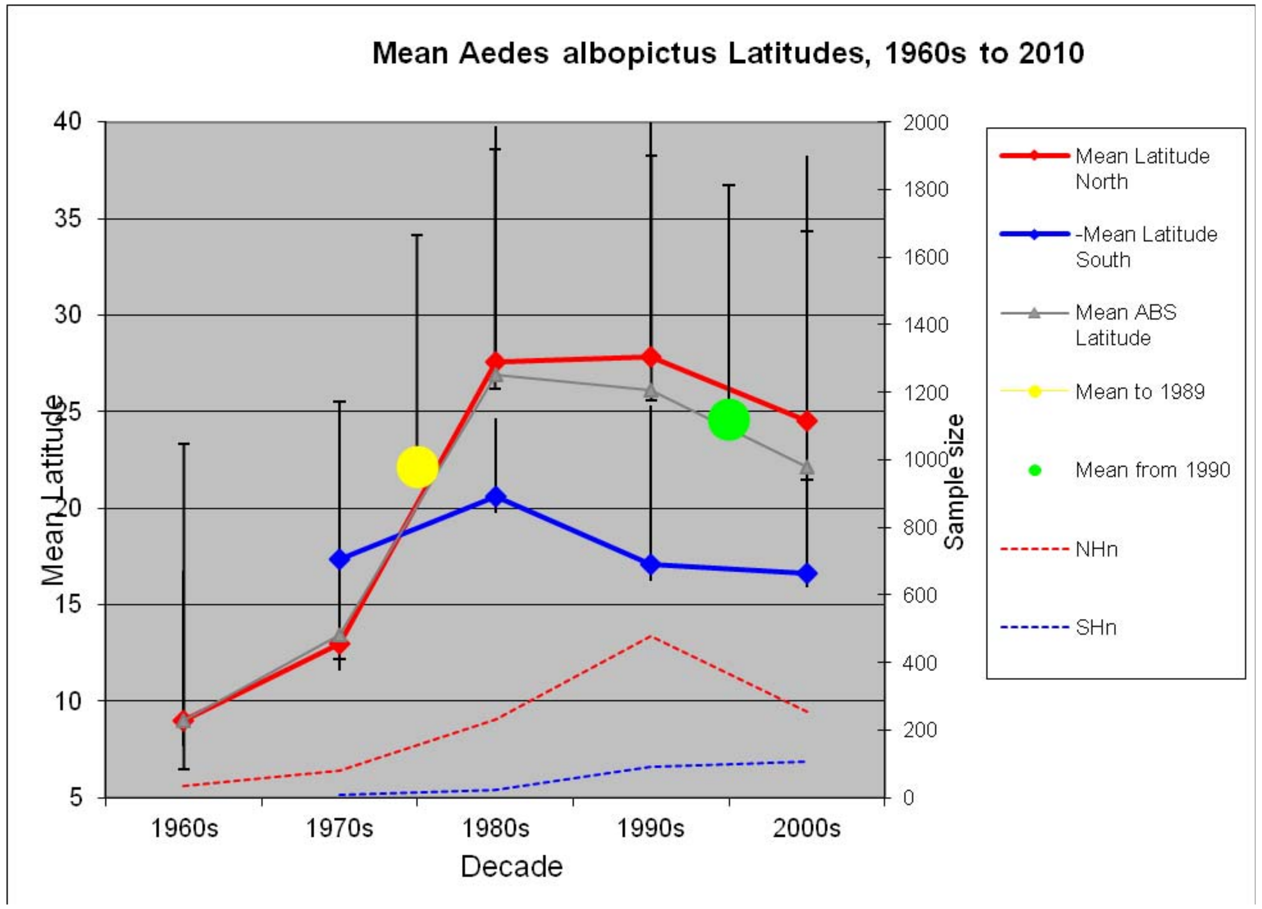

Figure 2a. Risk map for dengue using 10 predictor variables (selected from 70 Fourier processed meteorological variables). Resolution 1/3rd degree.

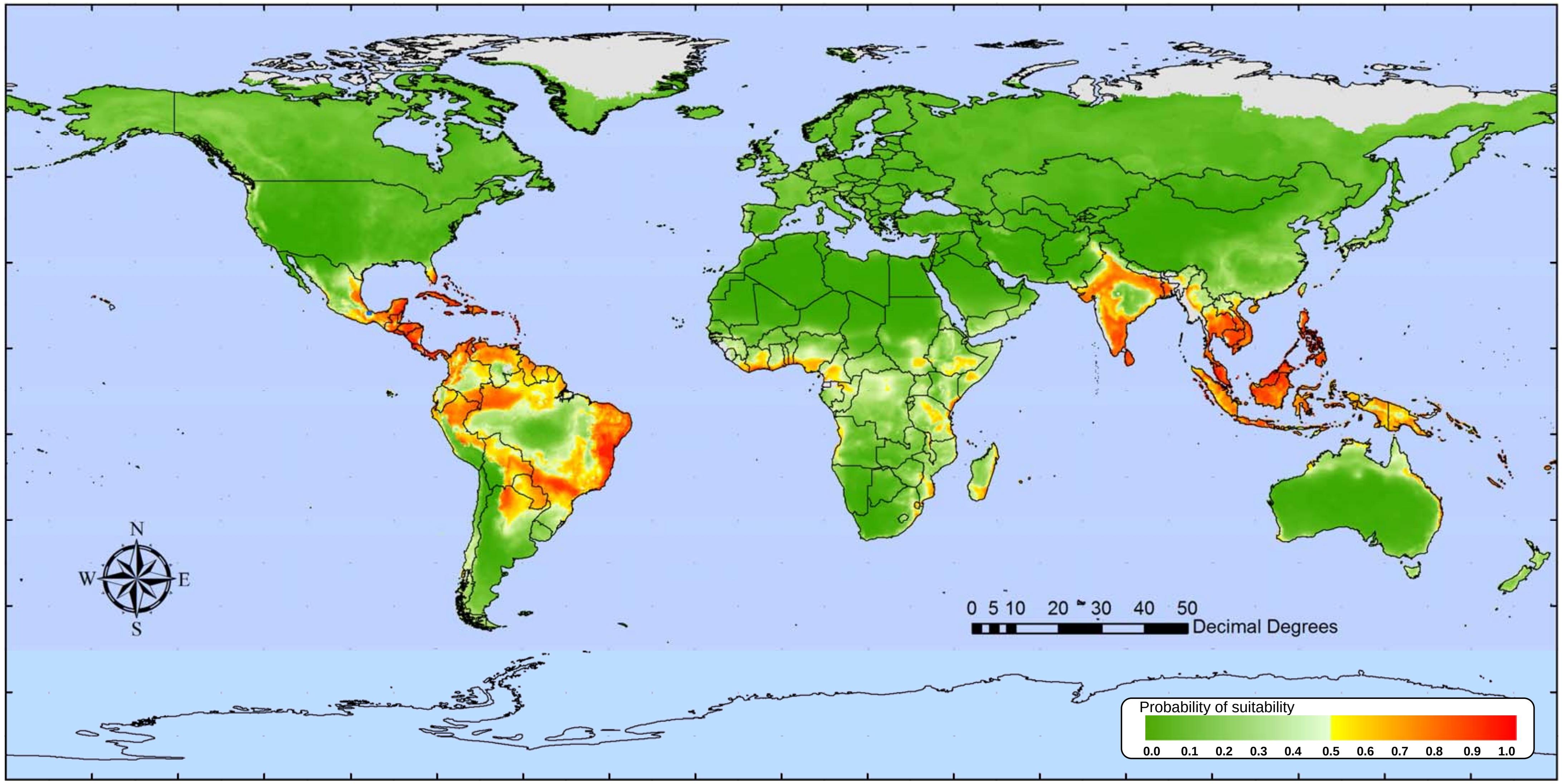

Figure 2b. Risk map for dengue using 10 predictor variables (selected from 70 Fourier processed meteorological variables). Resolution 1/2 degree.

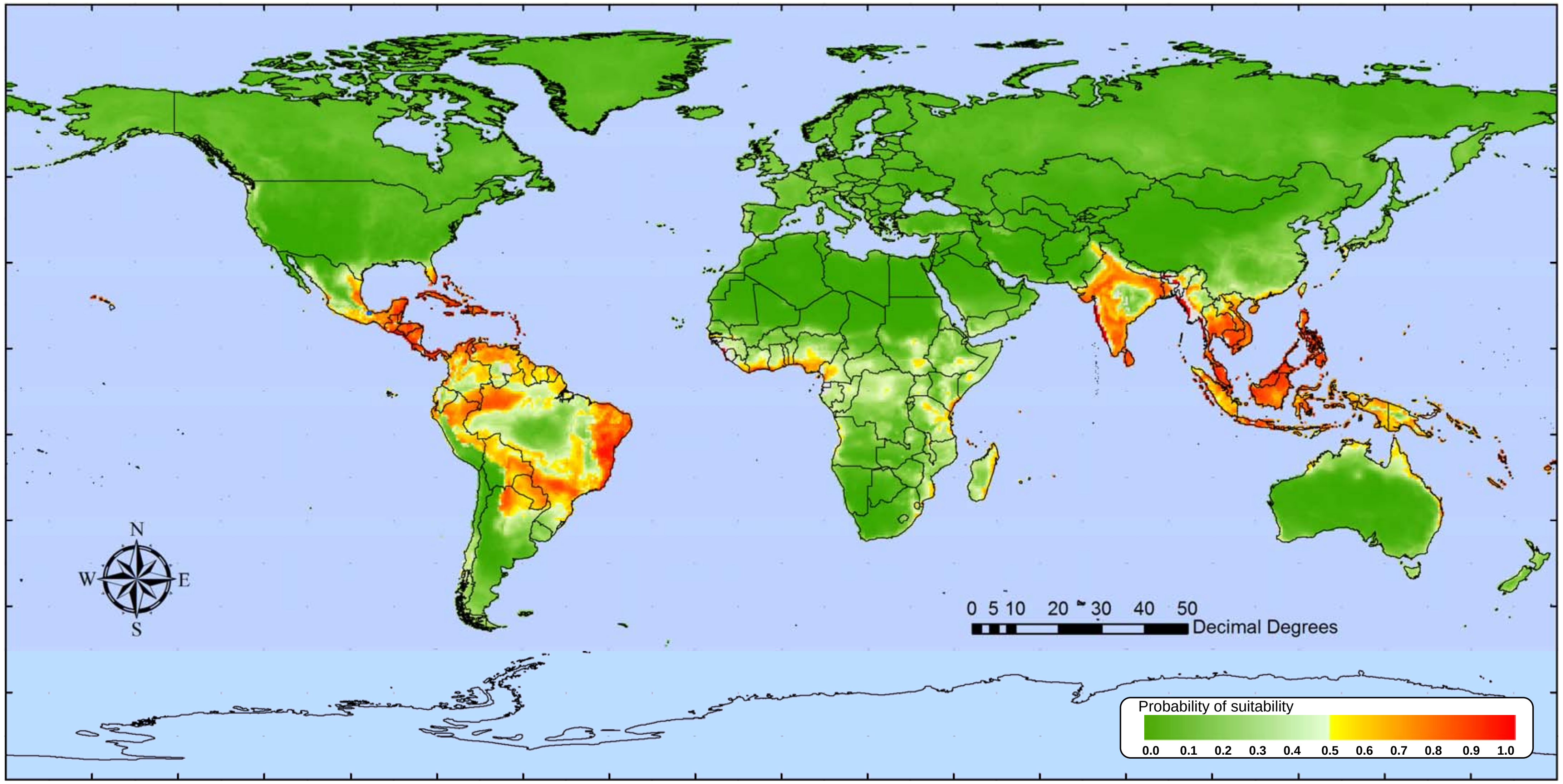

Figure 2c. Risk map for dengue using 10 predictor variables (selected from 70 Fourier processed meteorological variables). Resolution 1 degree.

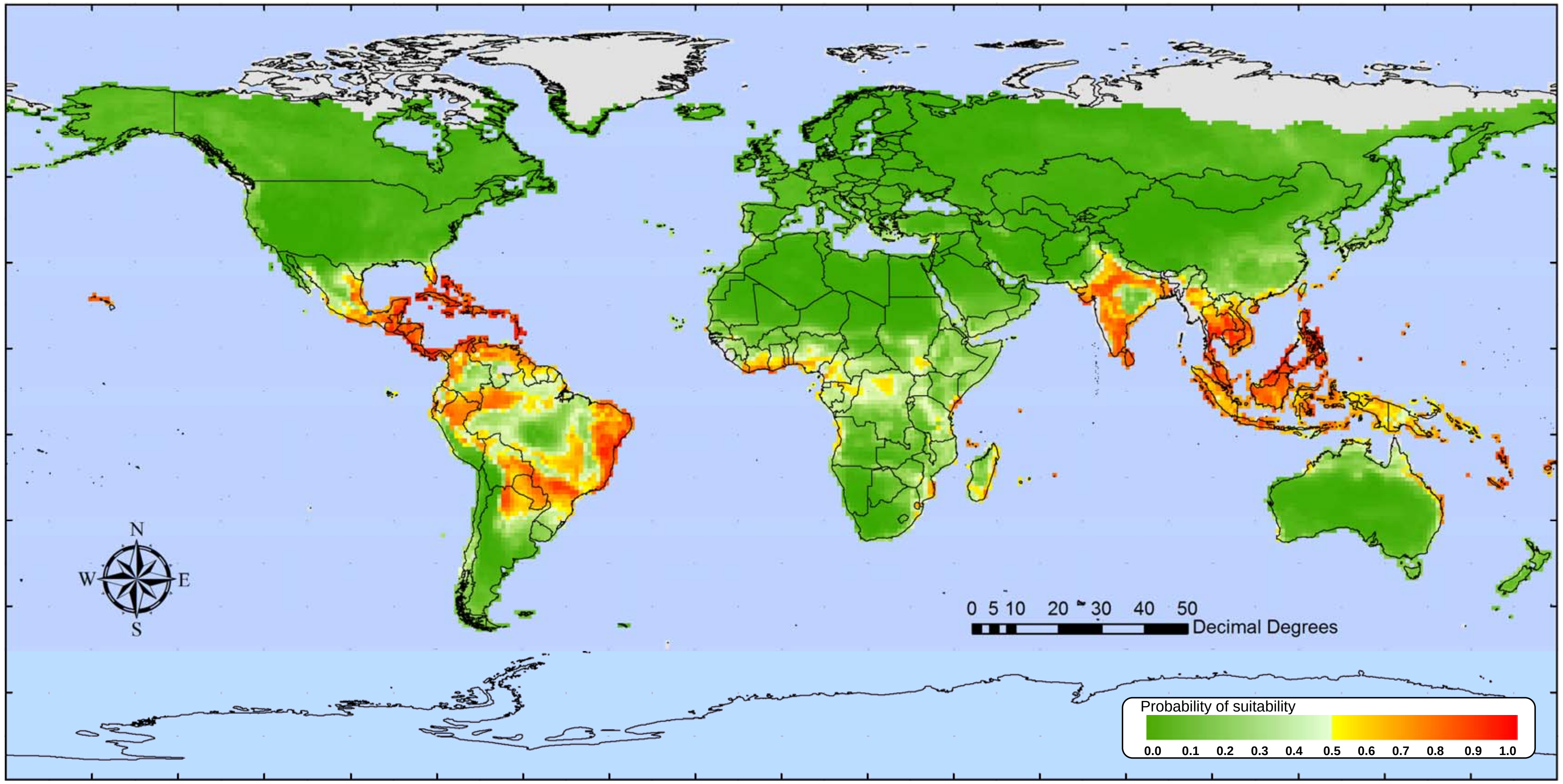

Figure 2d. Risk map for dengue using 10 predictor variables (selected from 70 Fourier processed meteorological variables). Resolution 2 degrees.

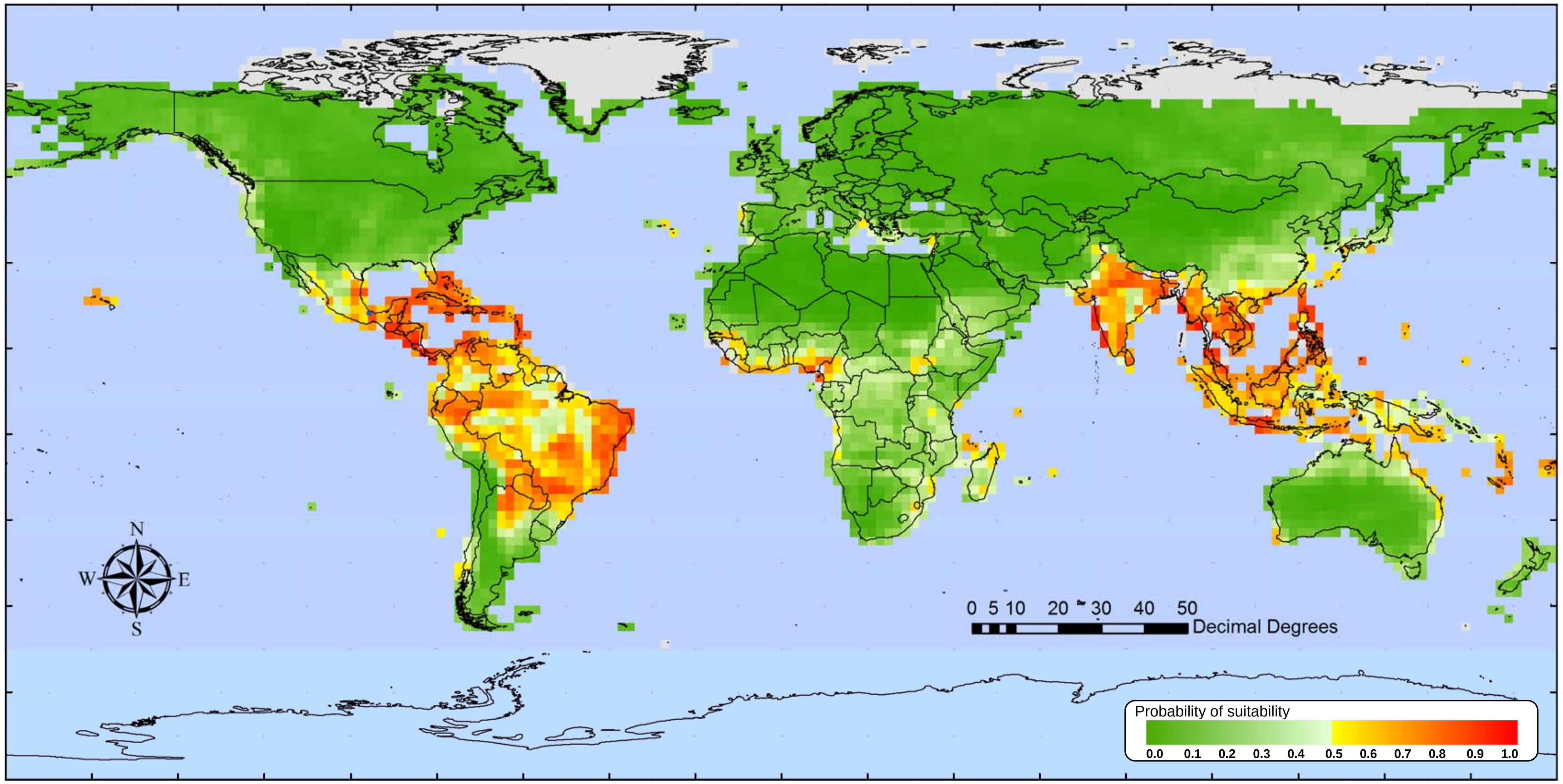

Figure 3. Risk maps for *Aedes aegypti* (row a)), *Aedes albopictus* (row b)), Dengue without vectors (row c)) and Dengue with vectors (row d)) at the present time, and for the years 2020, 2040 and 2080 under HadCM3 scenario B1a. The last panel in each row is a difference image (2080 – Present day): DECREASING suitability is in red in these images and INCREASING suitability in green.

Figure 3, Row a. *Aedes aegypti,* scenario B1a, Present day.

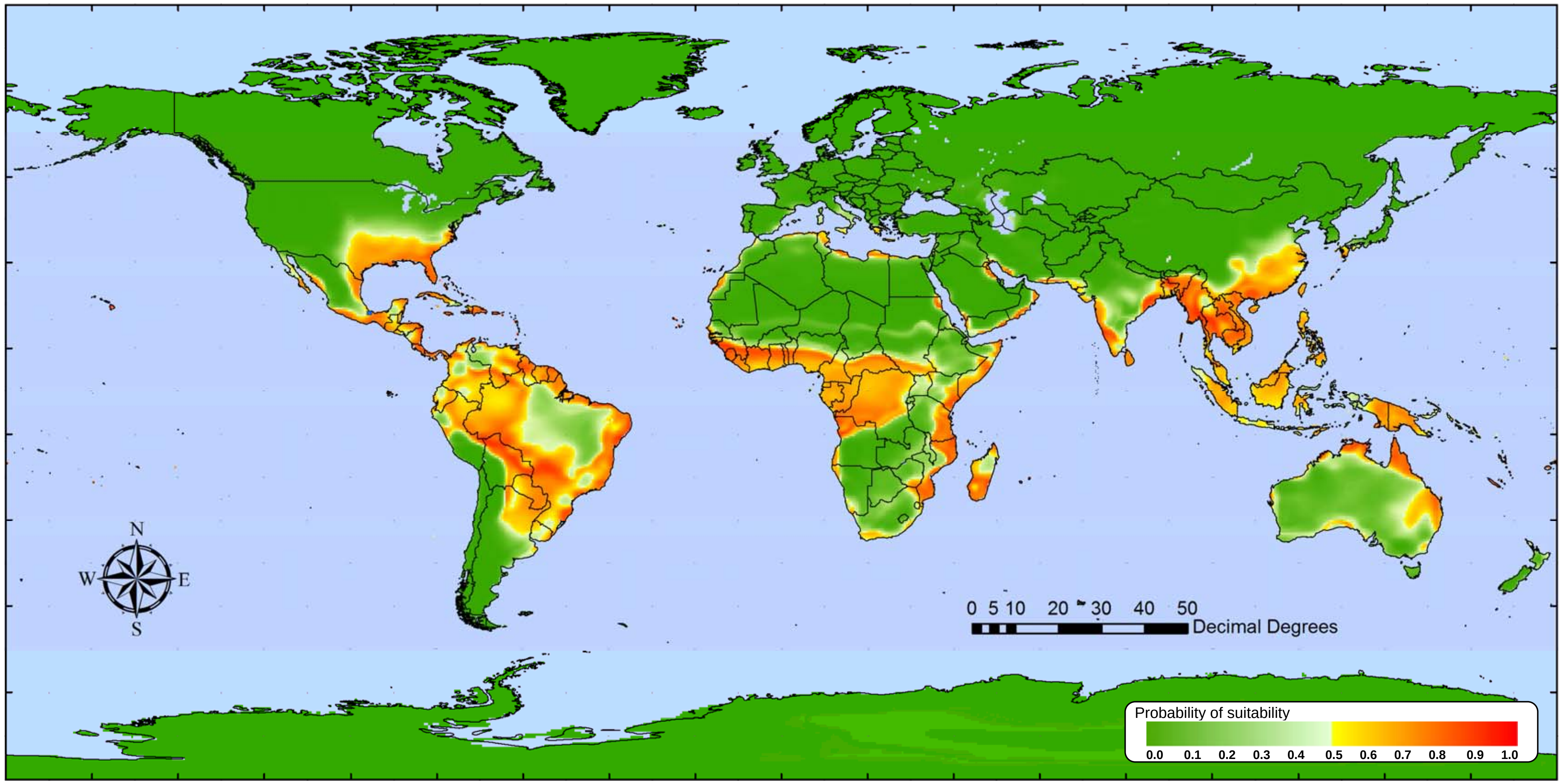

Figure 3, Row a. *Aedes aegypti,* scenario B1a, Year 2020.

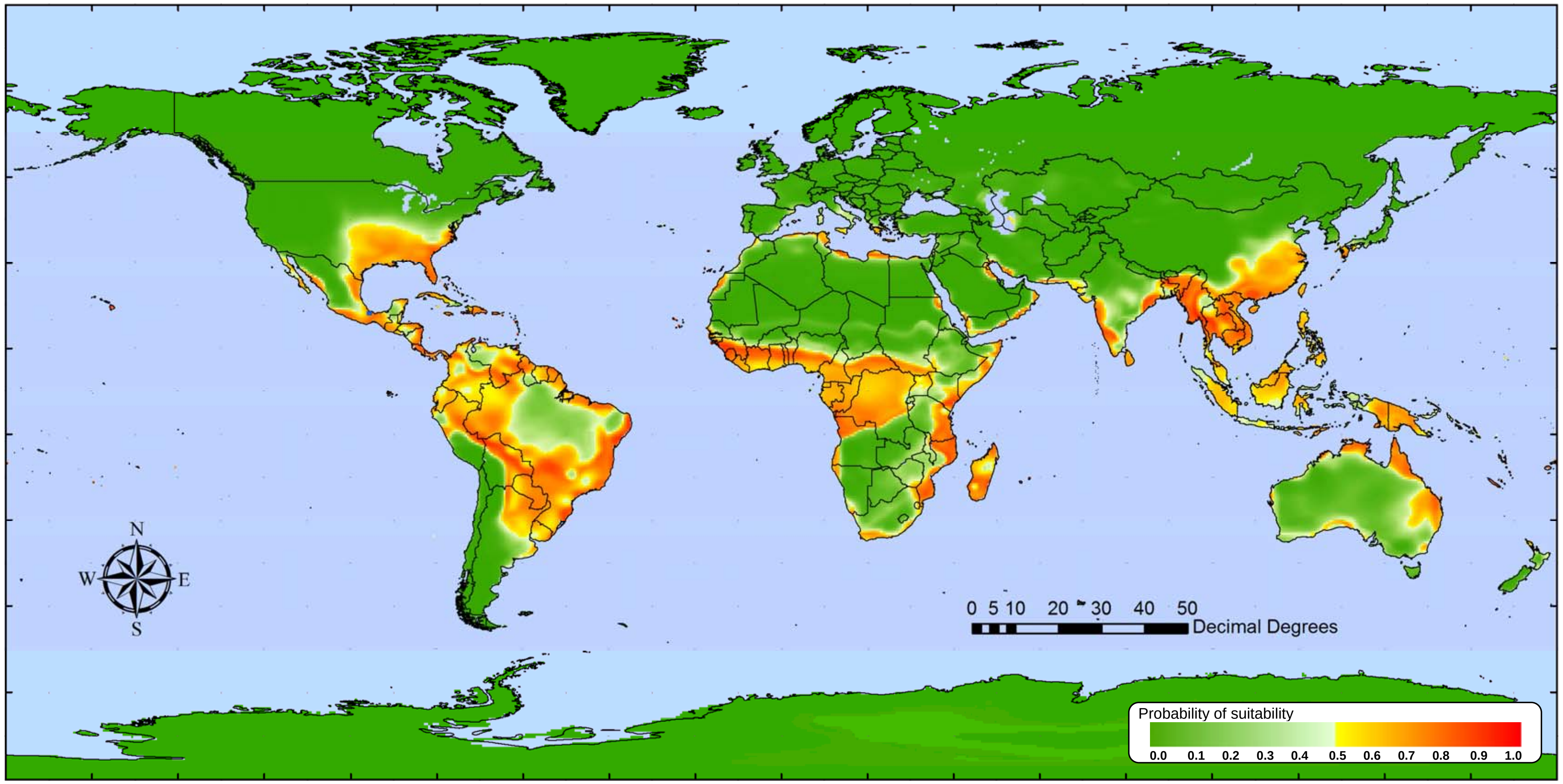

Figure 3, Row a. *Aedes aegypti,* scenario B1a, Year 2040.

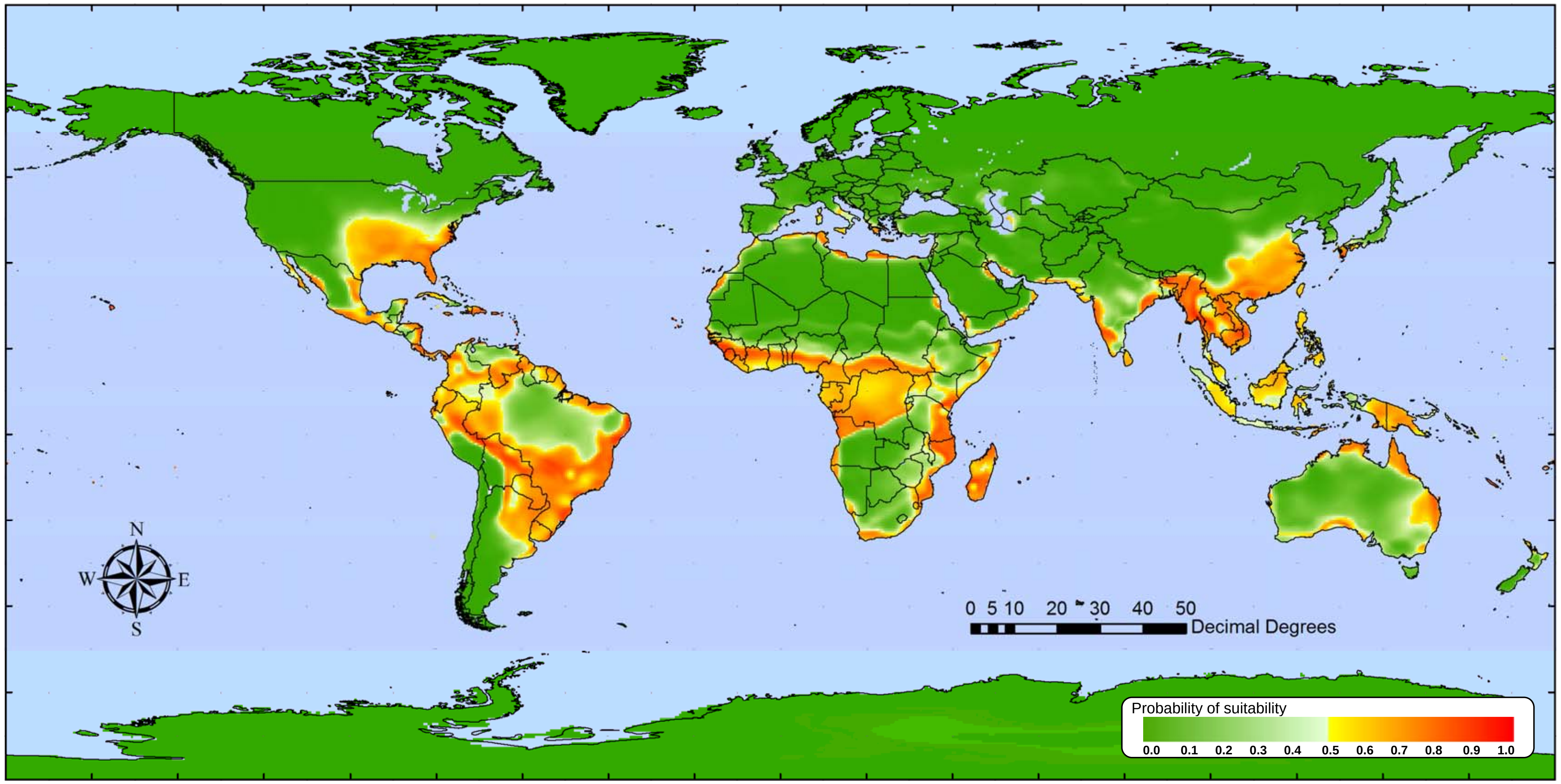

Figure 3, Row a. *Aedes aegypti,* scenario B1a, Year 2080.

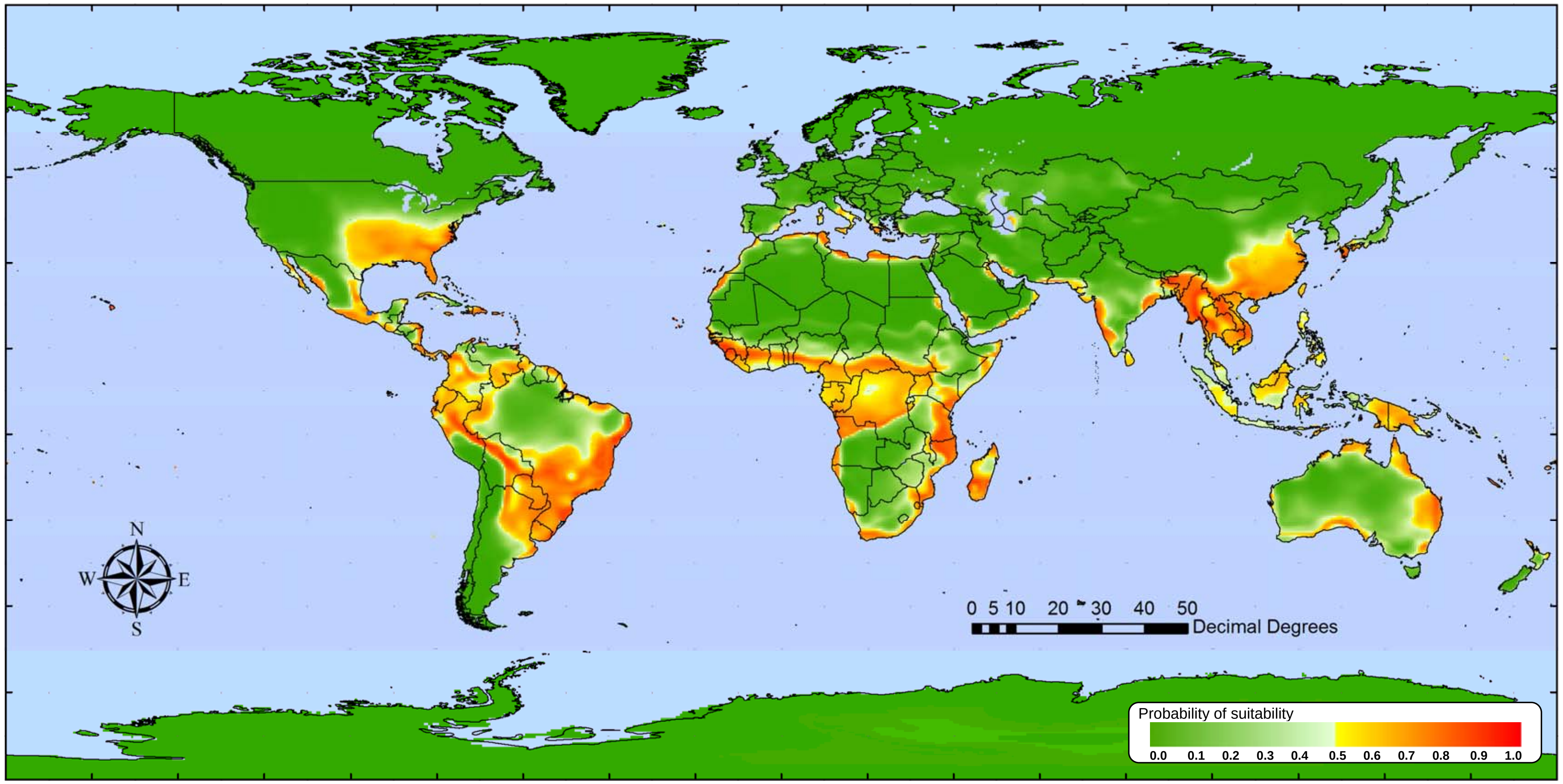

Figure 3, Row a. *Aedes aegypti,* scenario B1a, difference image (2080 – Present day). DECREASING suitability is in red in this image and INCREASING suitability in green.

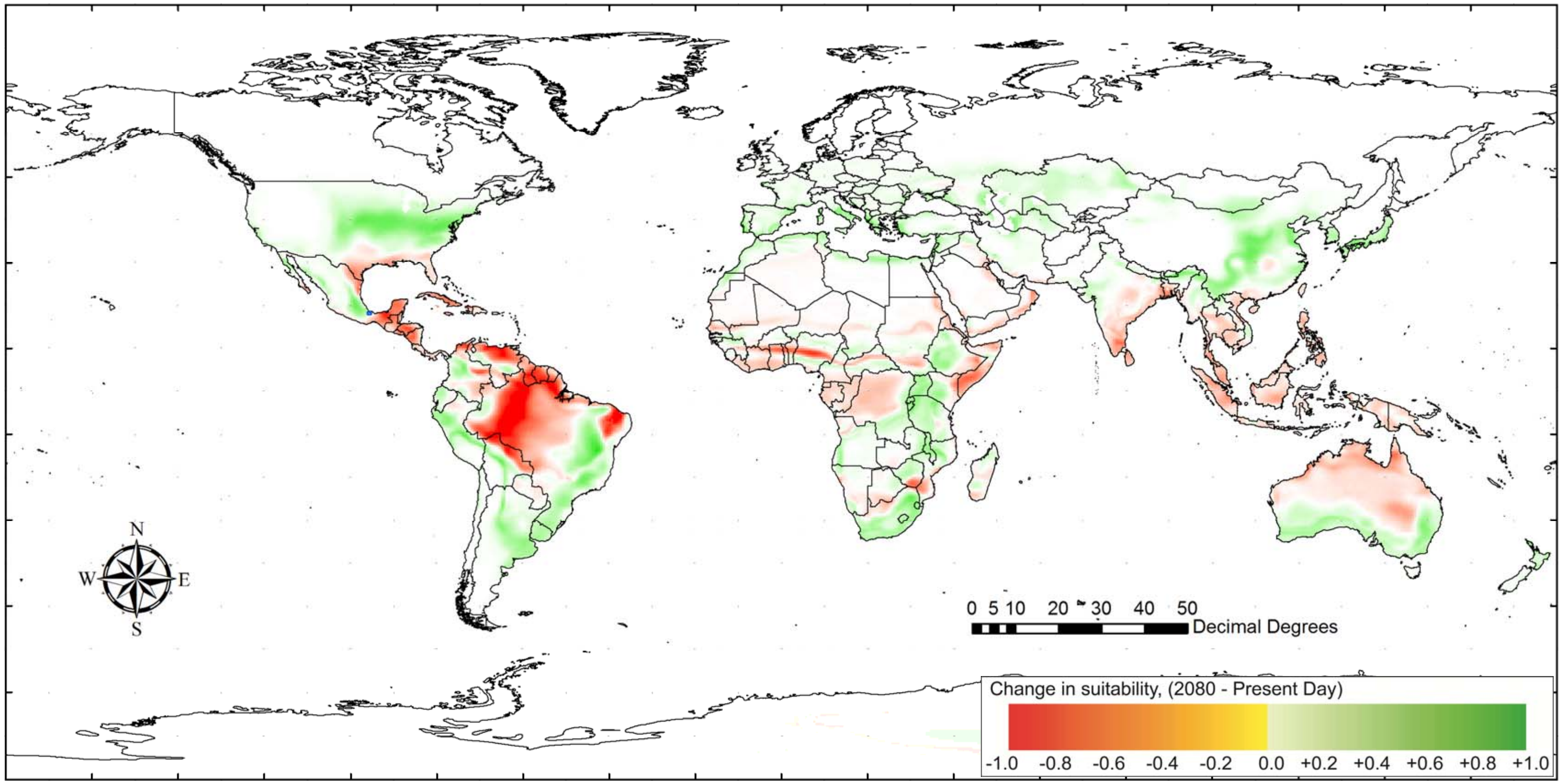

Figure 3, Row b. *Aedes albopictus* , scenario B1a, Present day.

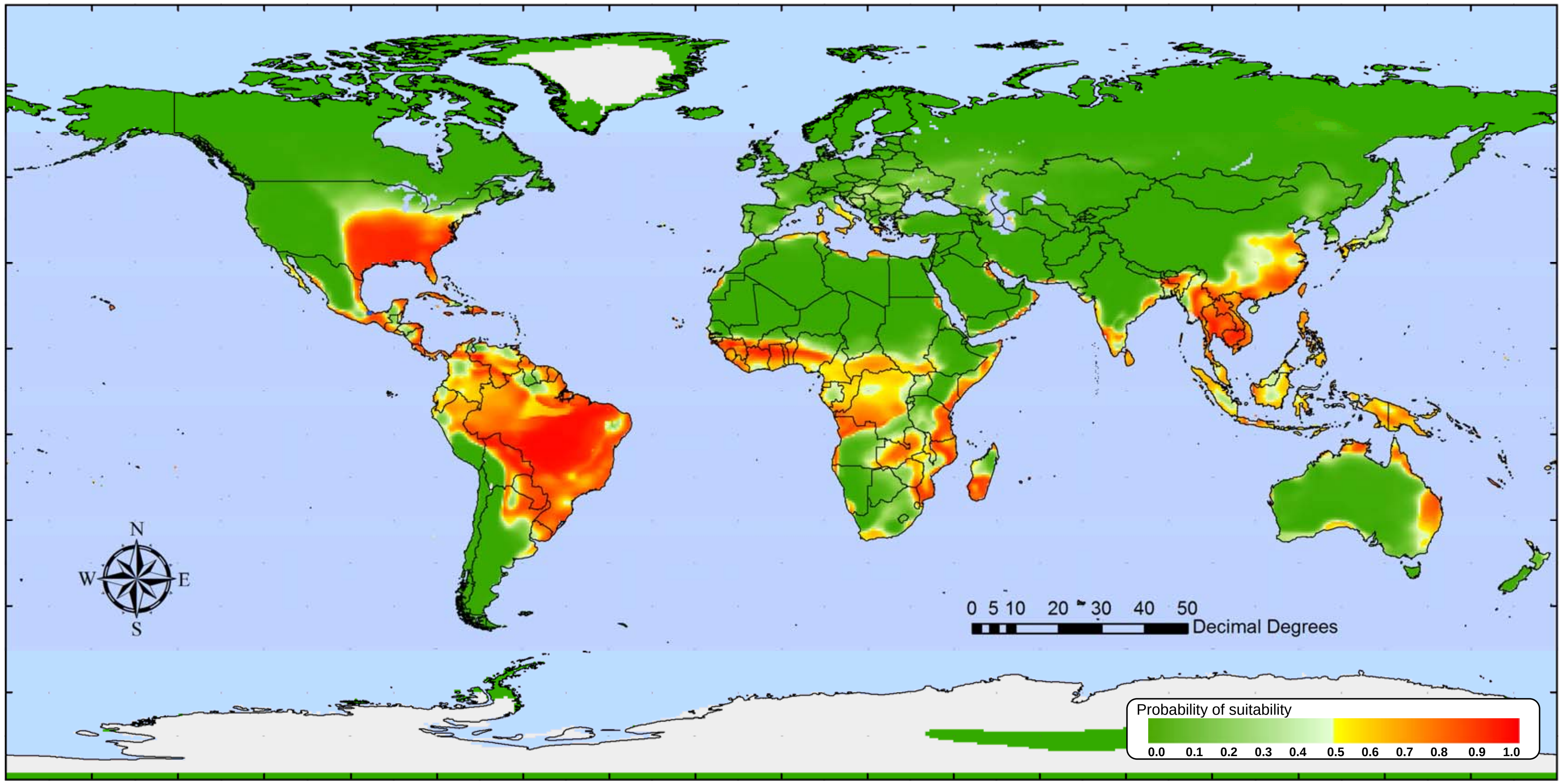

Figure 3, Row b. *Aedes albopictus ,* scenario B1a, Year 2020.

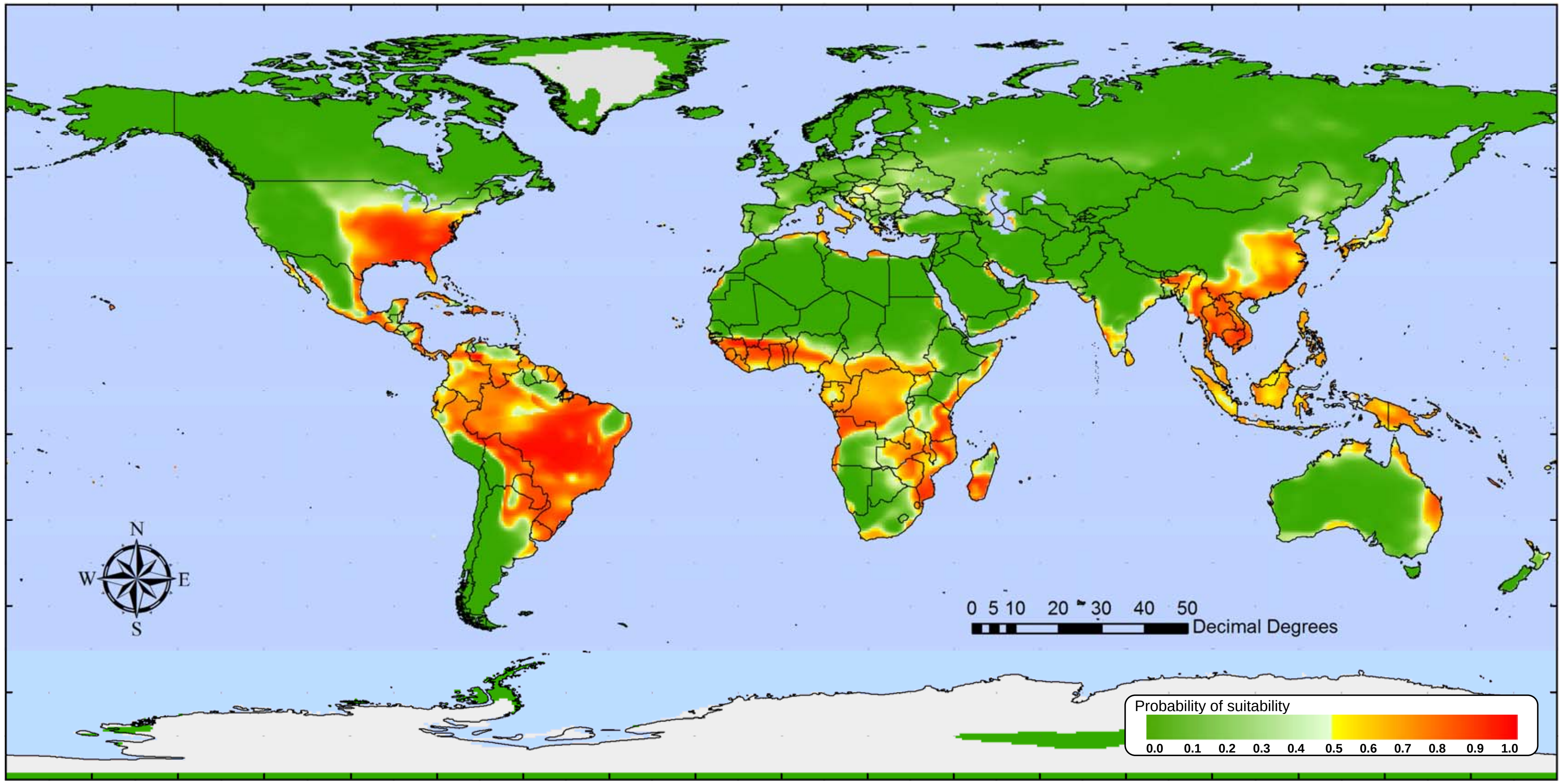

Figure 3, Row b. *Aedes albopictus* , scenario B1a, Year 2040.

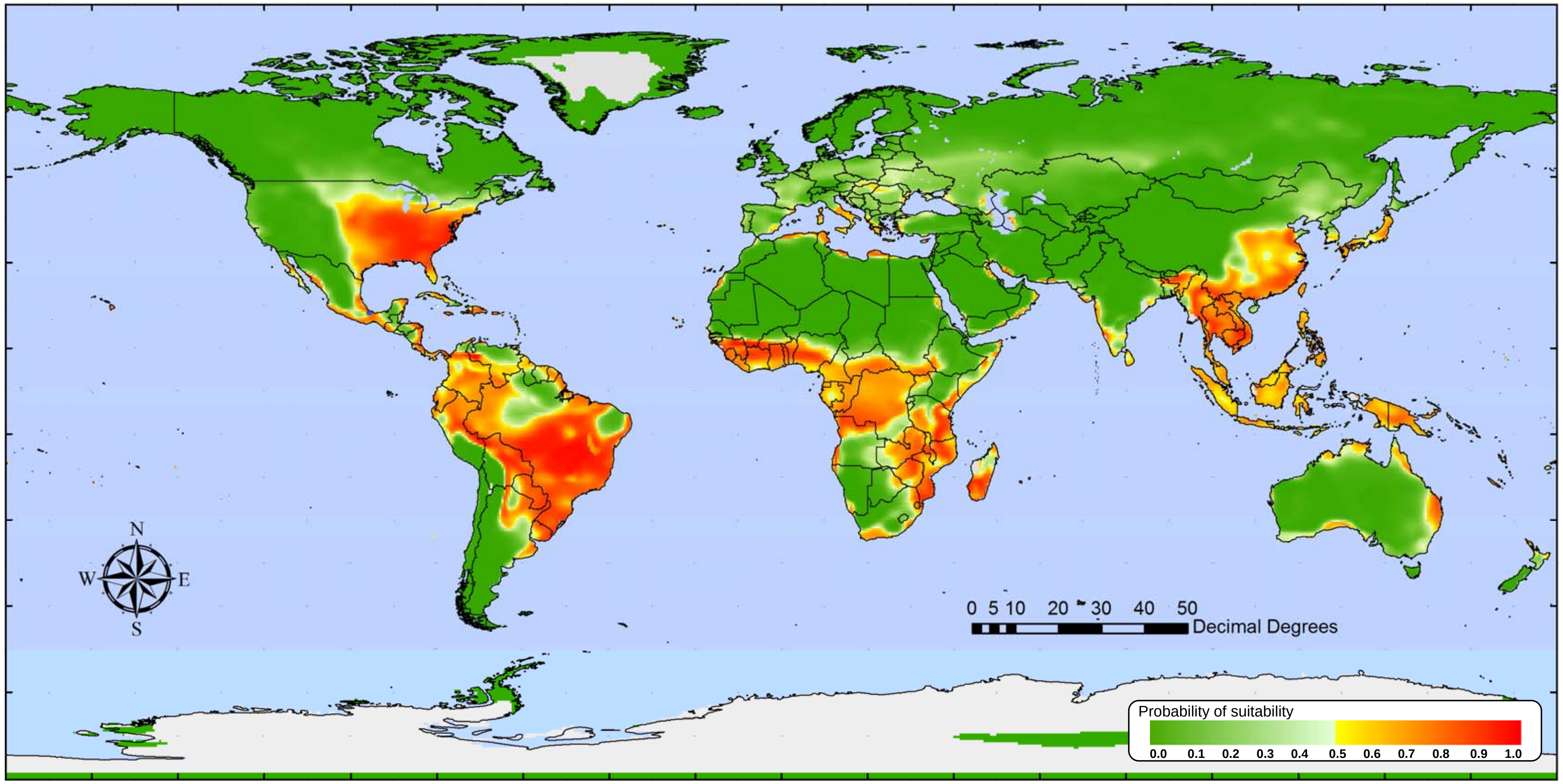

Figure 3, Row b. *Aedes albopictus,* scenario B1a, Year 2080.

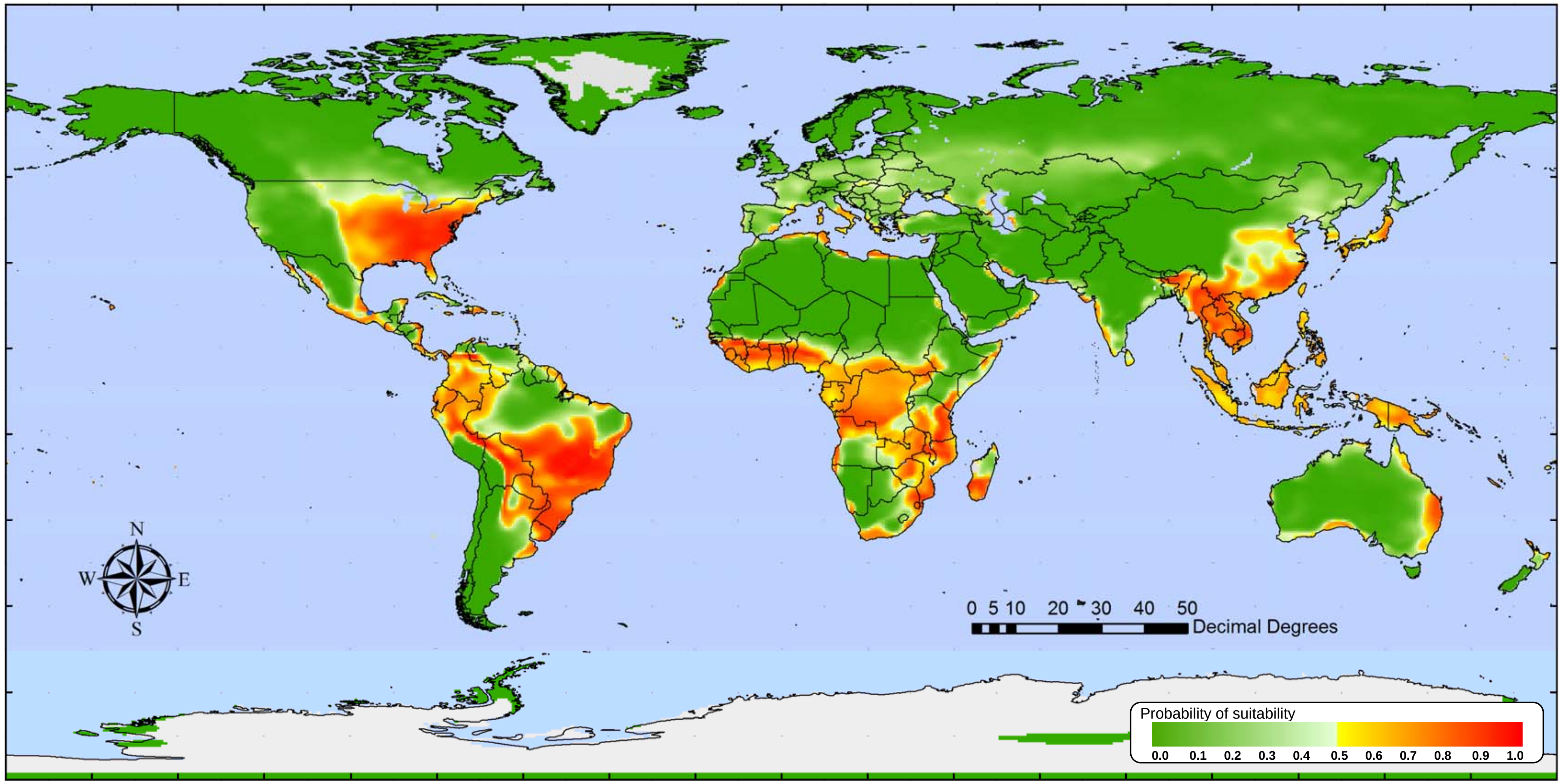

Figure 3, Row b. *Aedes albopictus ,* scenario B1a, difference image (2080 - Present day). DECREASING suitability is in red in this image and INCREASING suitability in green.

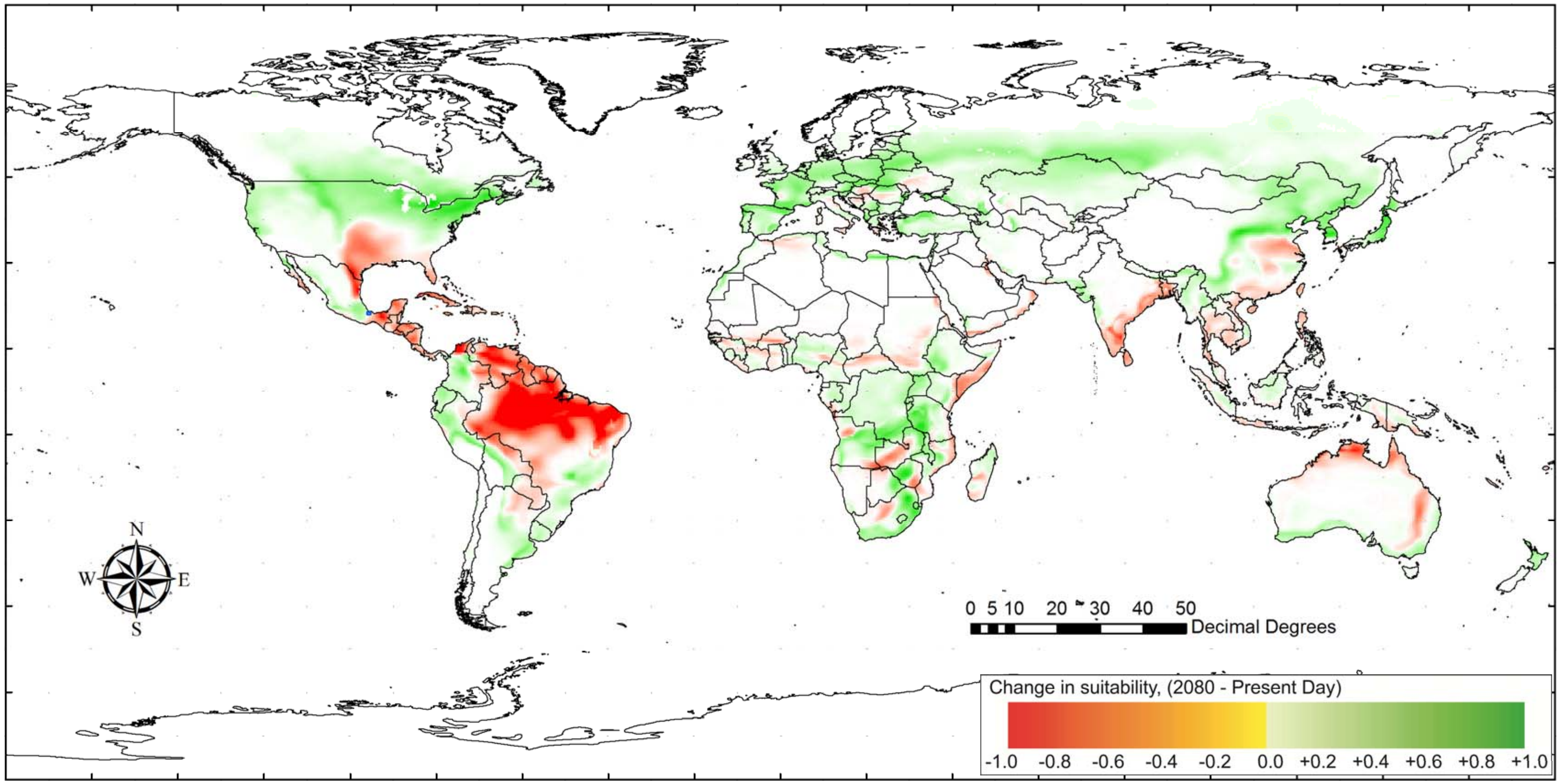

Figure 3, Row c. Dengue without vectors, scenario B1a, Present day.

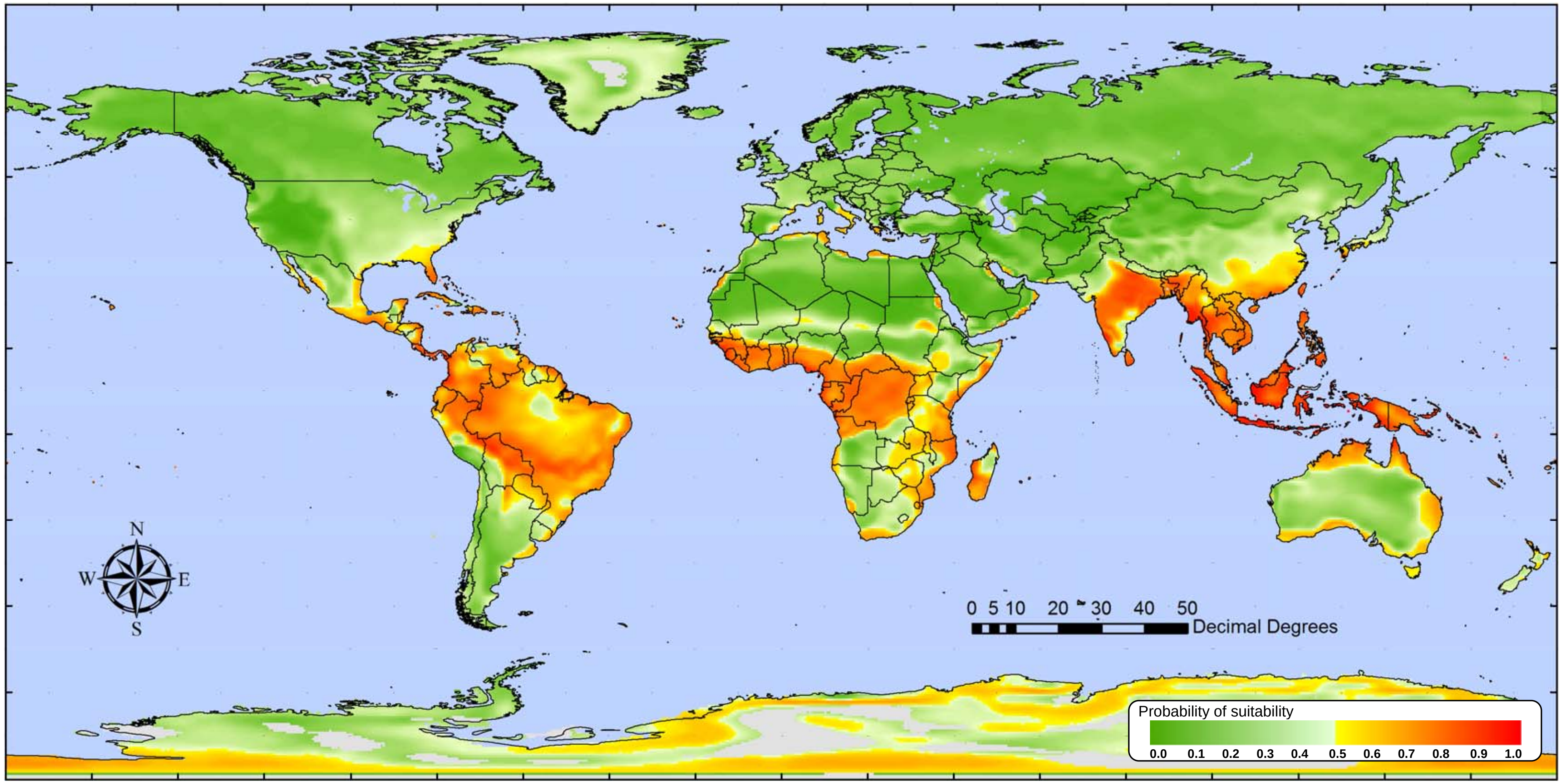

Figure 3, Row c. Dengue without vectors, scenario B1a, Year 2020.

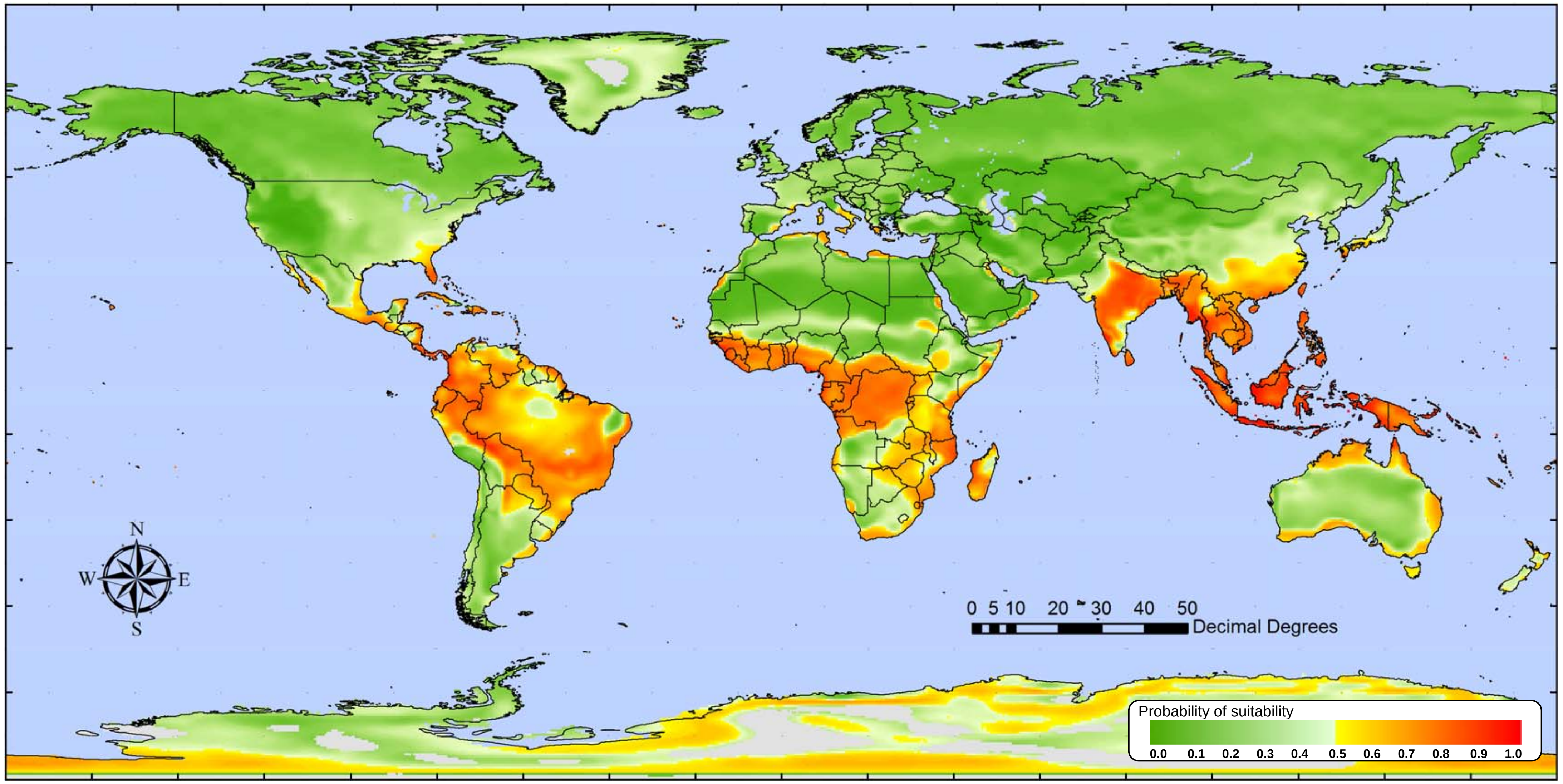

Figure 3, Row c. Dengue without vectors, scenario B1a, Year 2040.

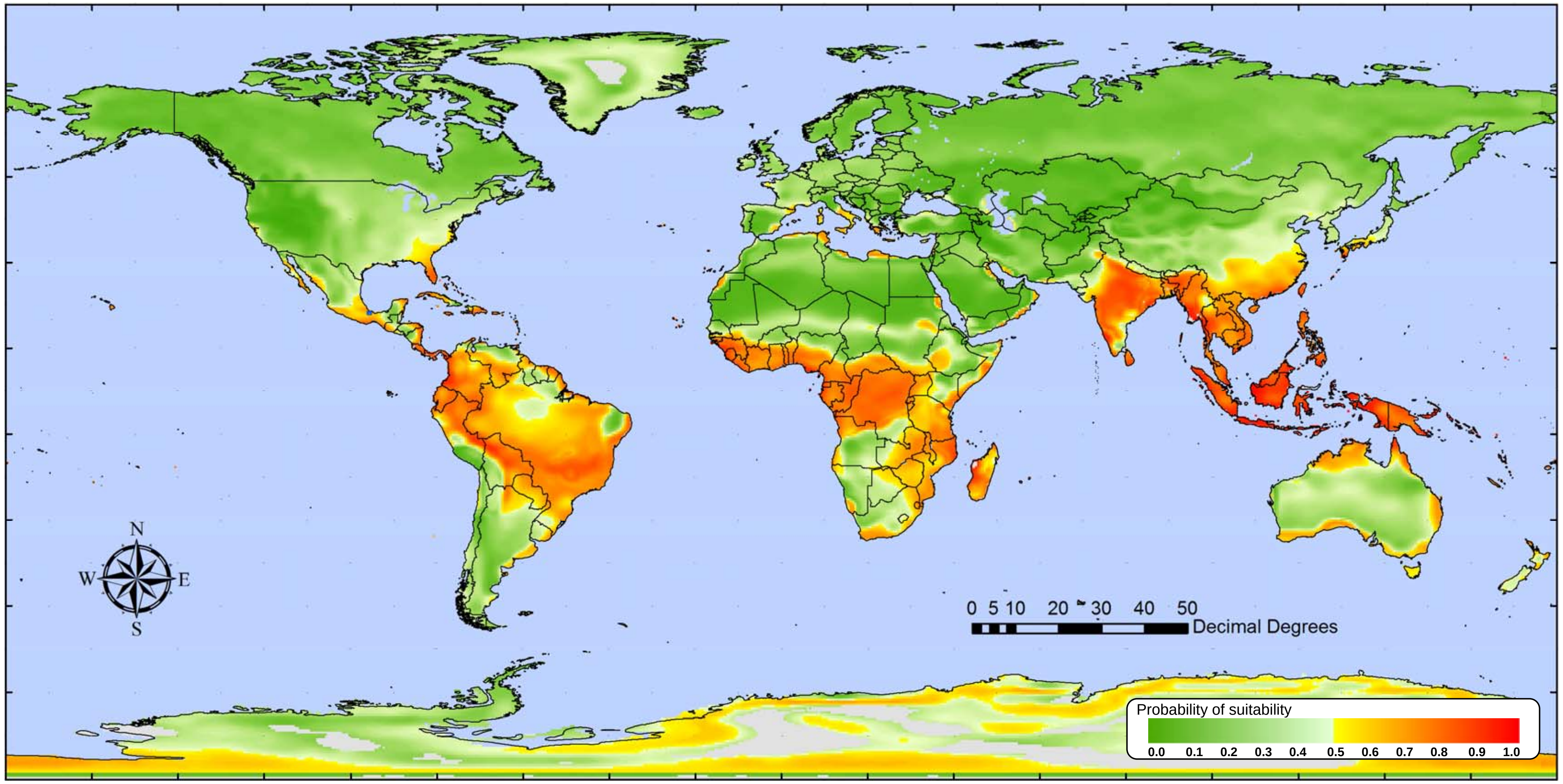

Figure 3, Row c. Dengue without vectors, scenario B1a, Year 2080.

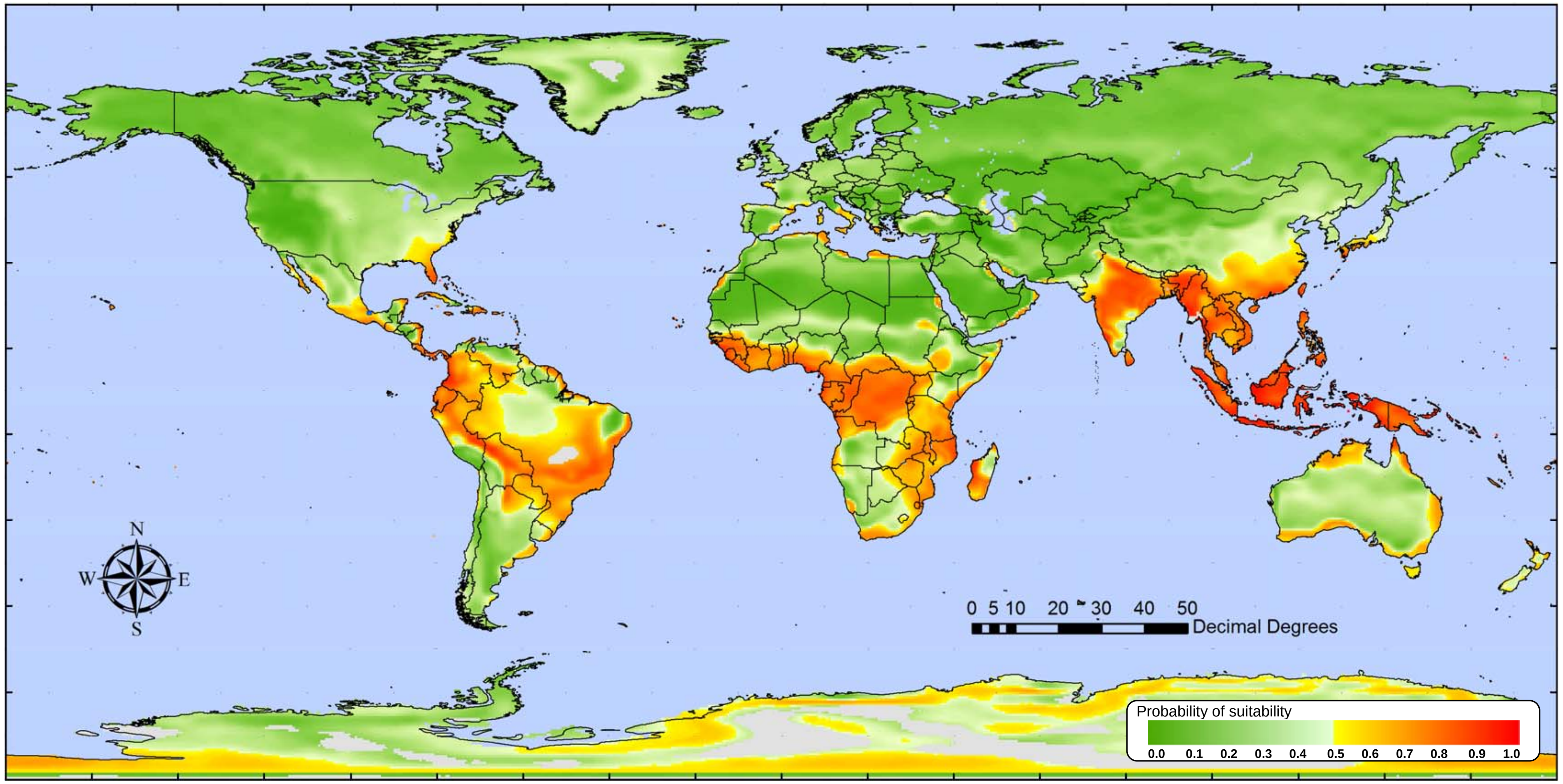

Figure 3, Row c. Dengue without vectors, scenario B1a, difference image (2080 - Present day). DECREASING suitability is in red in this image and INCREASING suitability in green.

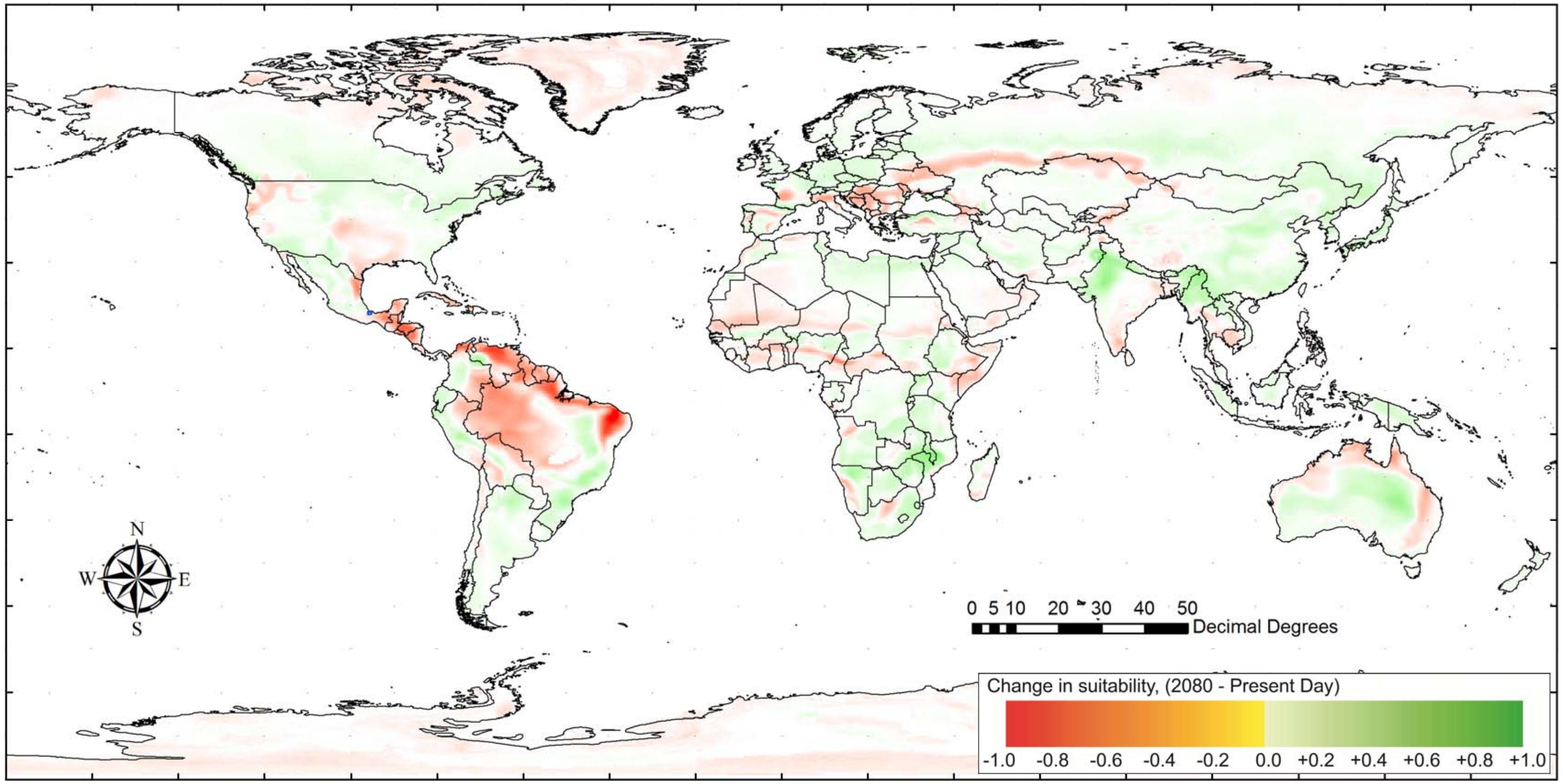

Figure 3, Row d. Dengue with vectors, scenario B1a, Present day.

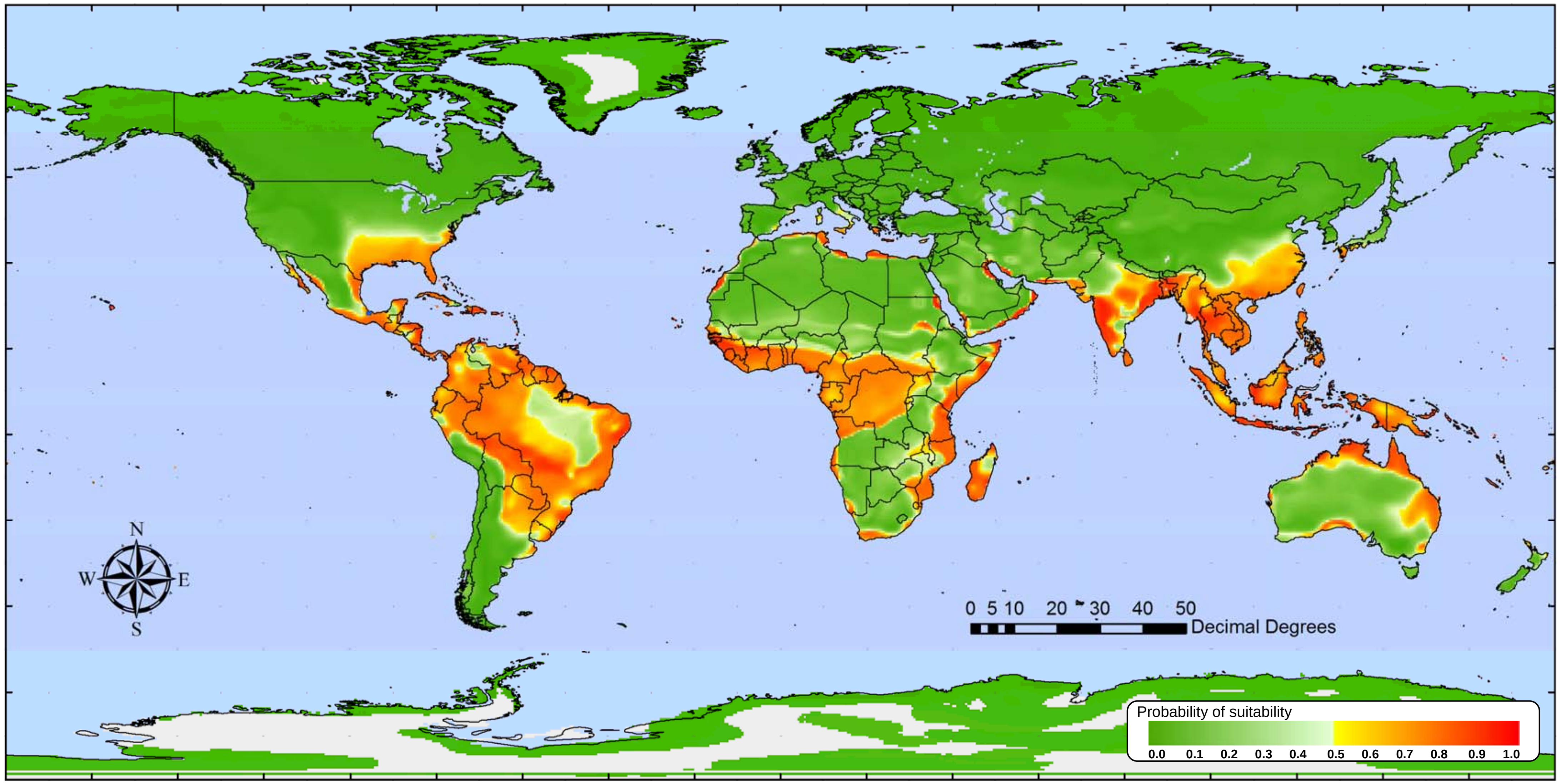

Figure 3, Row d. Dengue with vectors, scenario B1a, Year 2020.

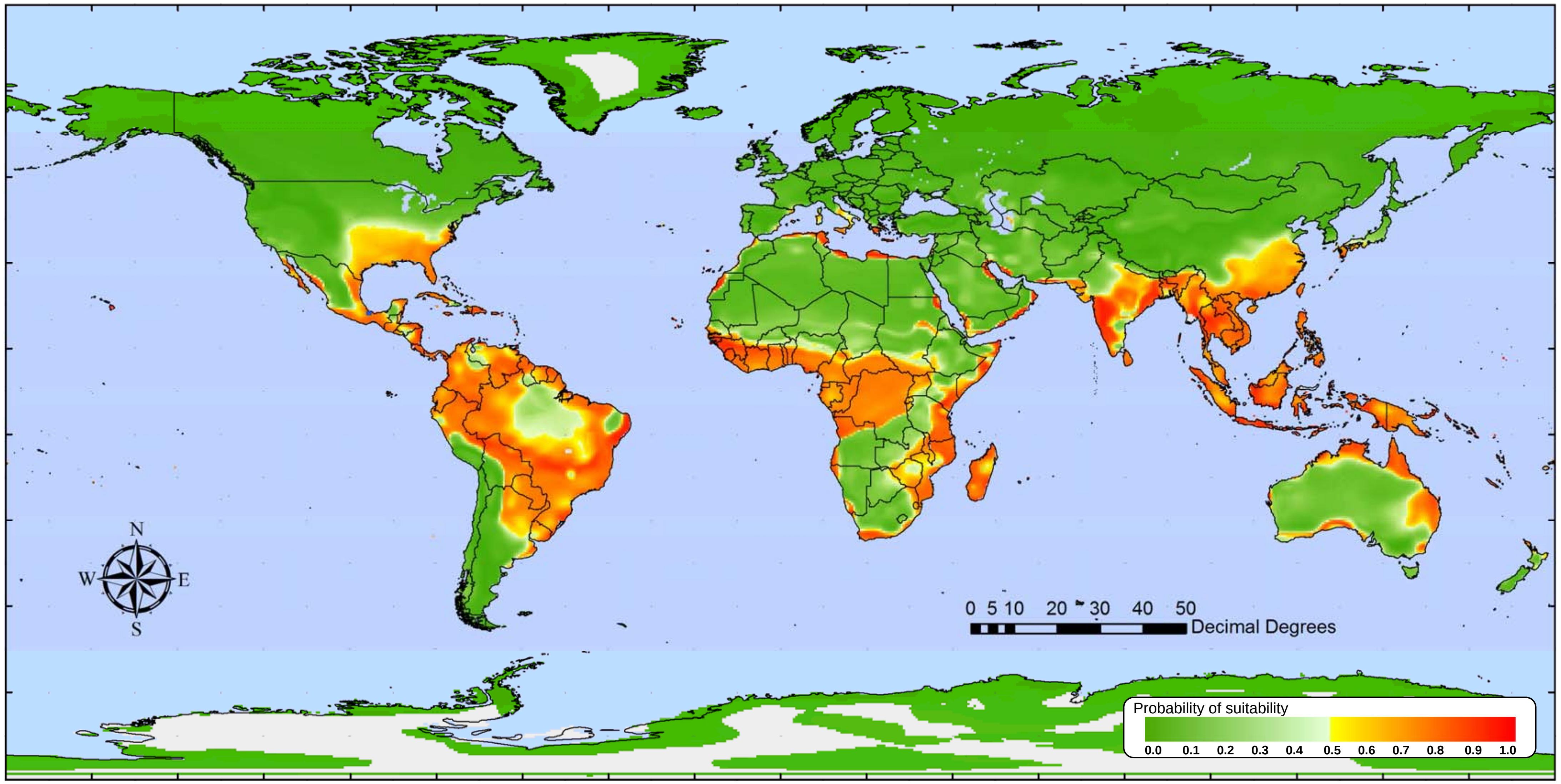

Figure 3, Row d. Dengue with vectors, scenario B1a, Year 2040.

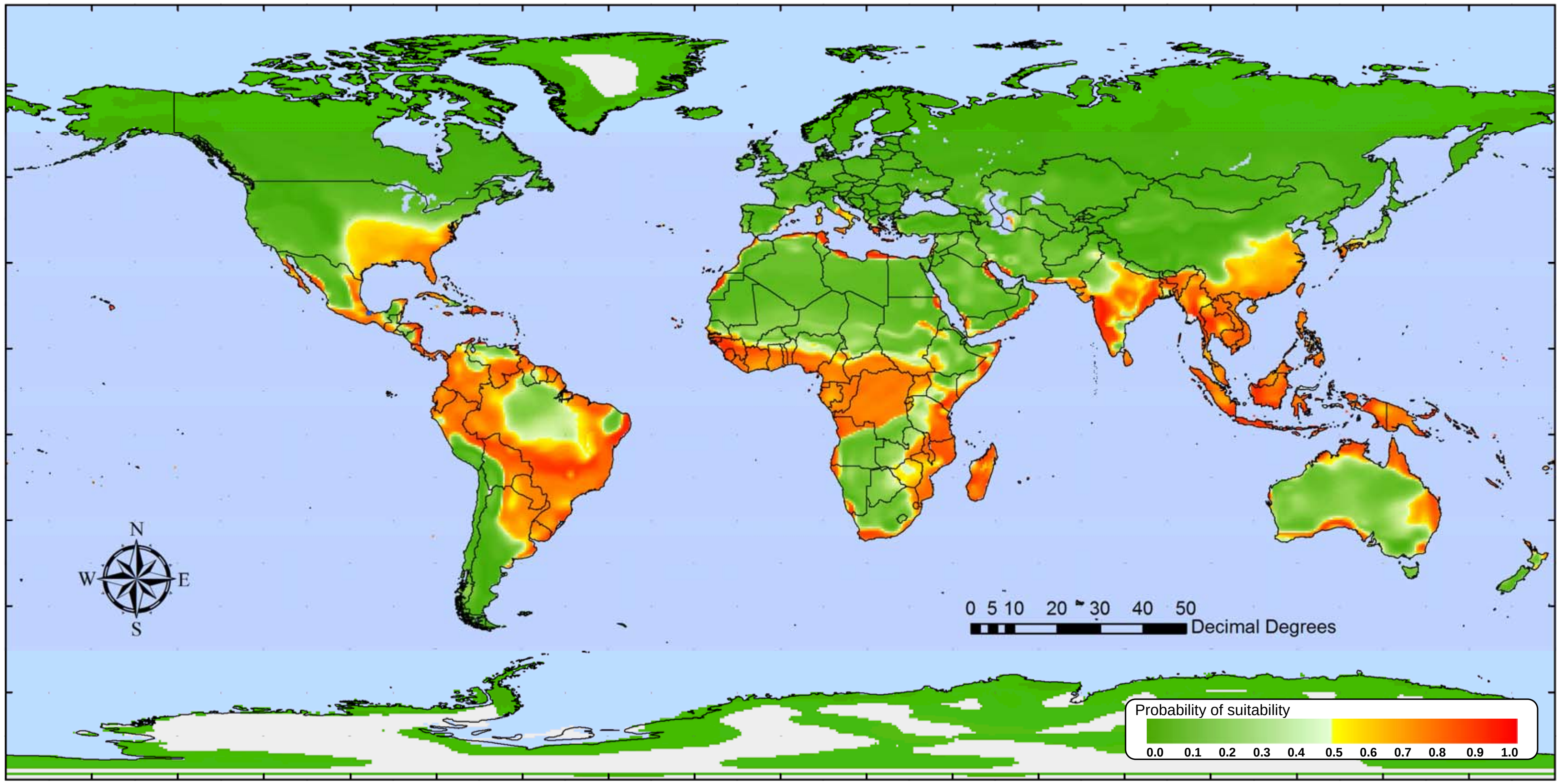

Figure 3, Row d. Dengue with vectors, scenario B1a, Year 2080.

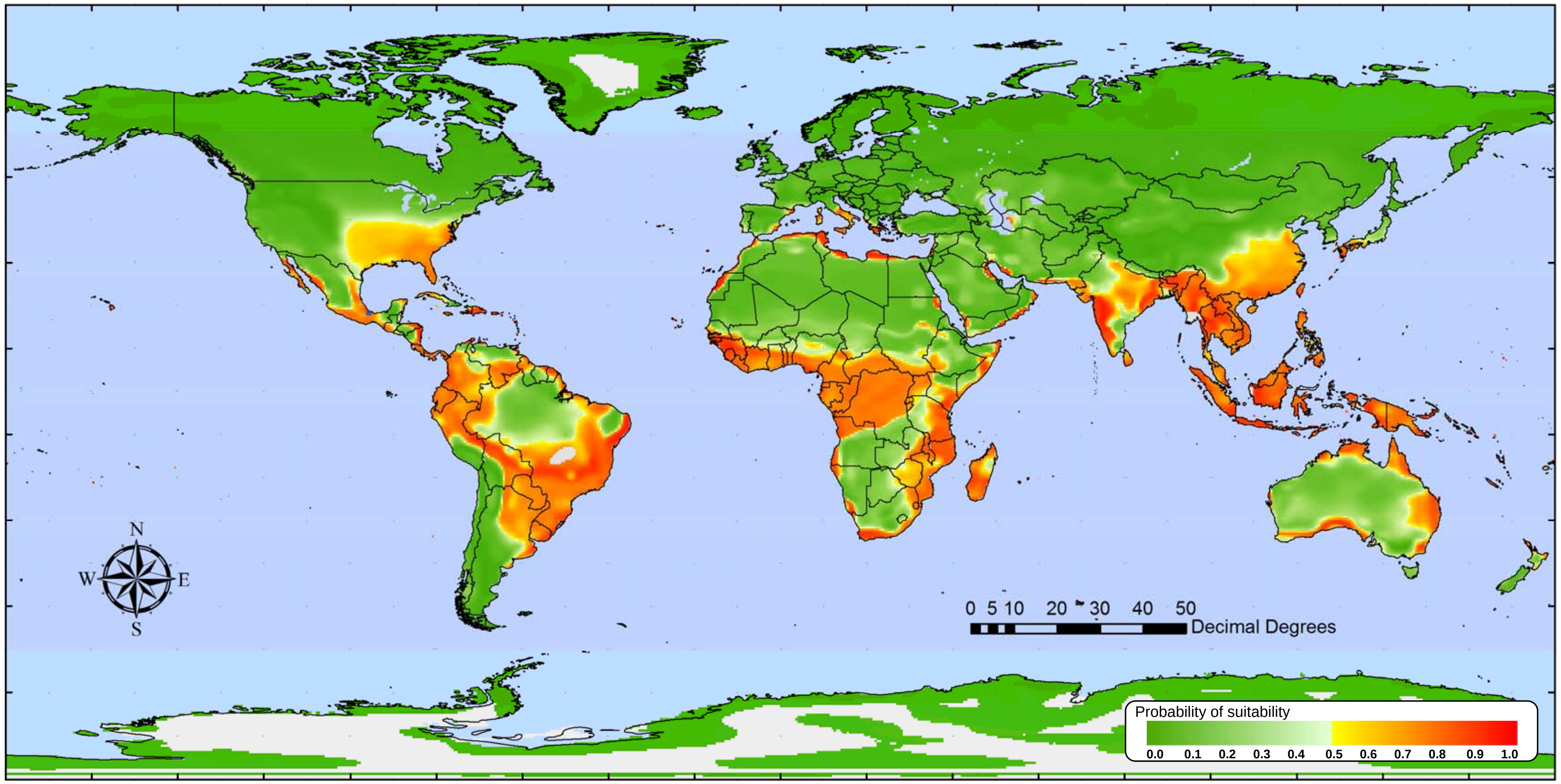

Figure 3, Row d. Dengue with vectors, scenario B1a, difference image (2080 - Present day). DECREASING suitability is in red in this image and INCREASING suitability in green.

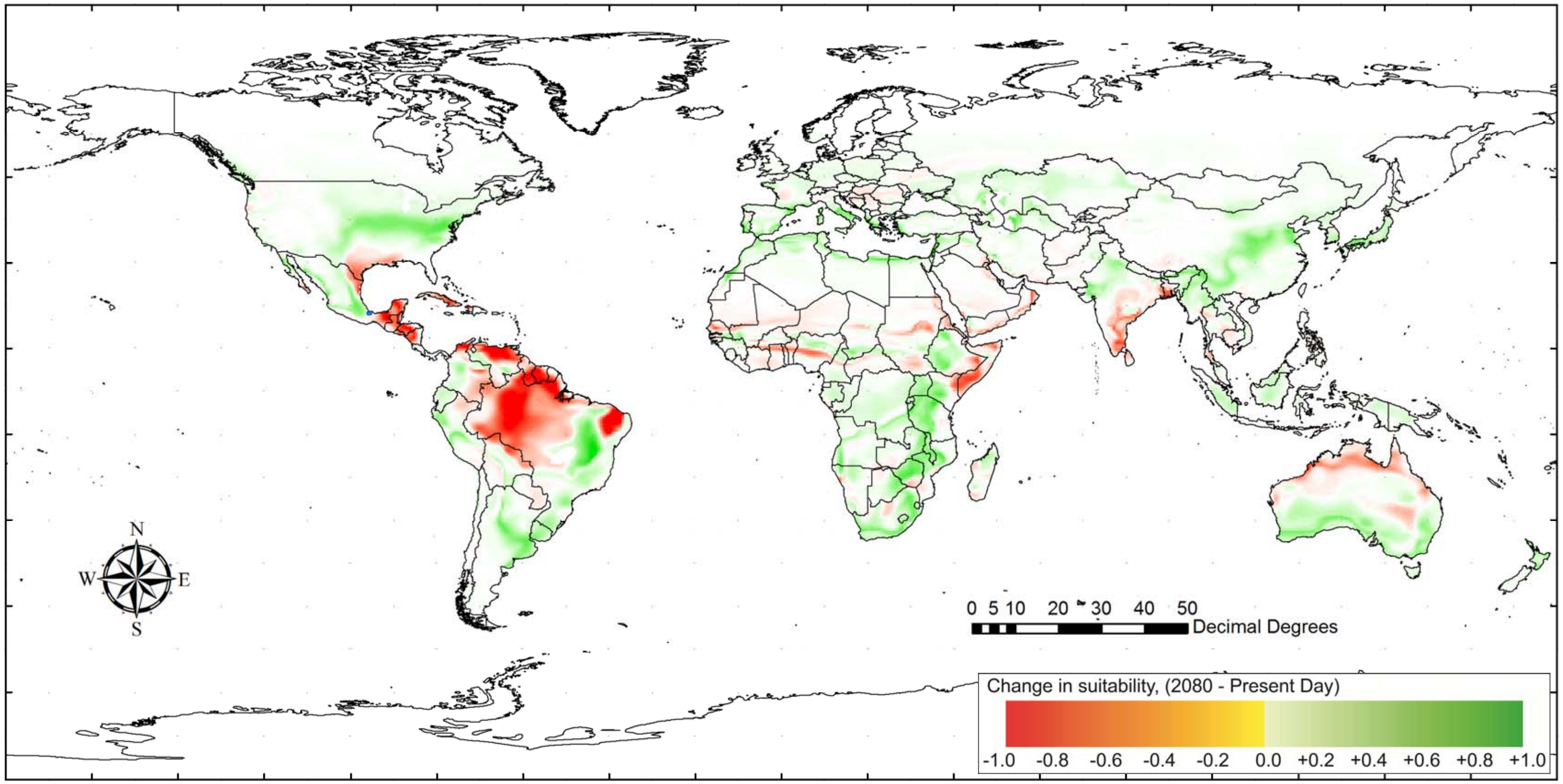

Figure 4. Risk maps for *Aedes aegypti* (row a)), *Aedes albopictus* (row b)), Dengue without vectors (row c)) and Dengue with vectors (row d)) at the present time, and for the years 2020, 2040 and 2080 under HadCM3 scenario A1F. The last panel in each row is a difference image (2080 – Present day): DECREASING suitability is in red in these images and INCREASING suitability in green.

Figure 4, Row a. *Aedes aegypti,* scenario A1F, Present day.

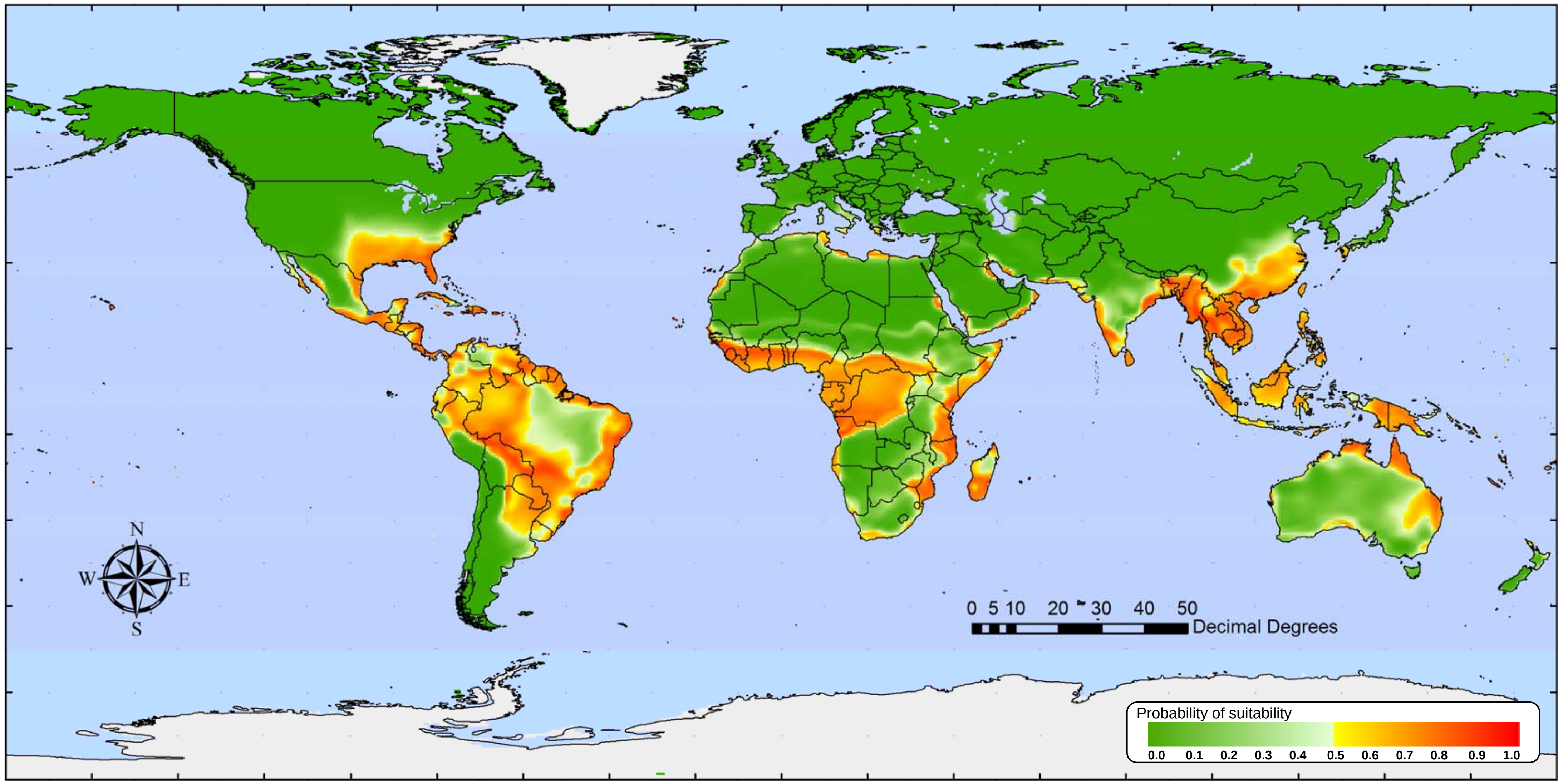

Figure 4, Row a. *Aedes aegypti,* scenario A1F, Year 2020.

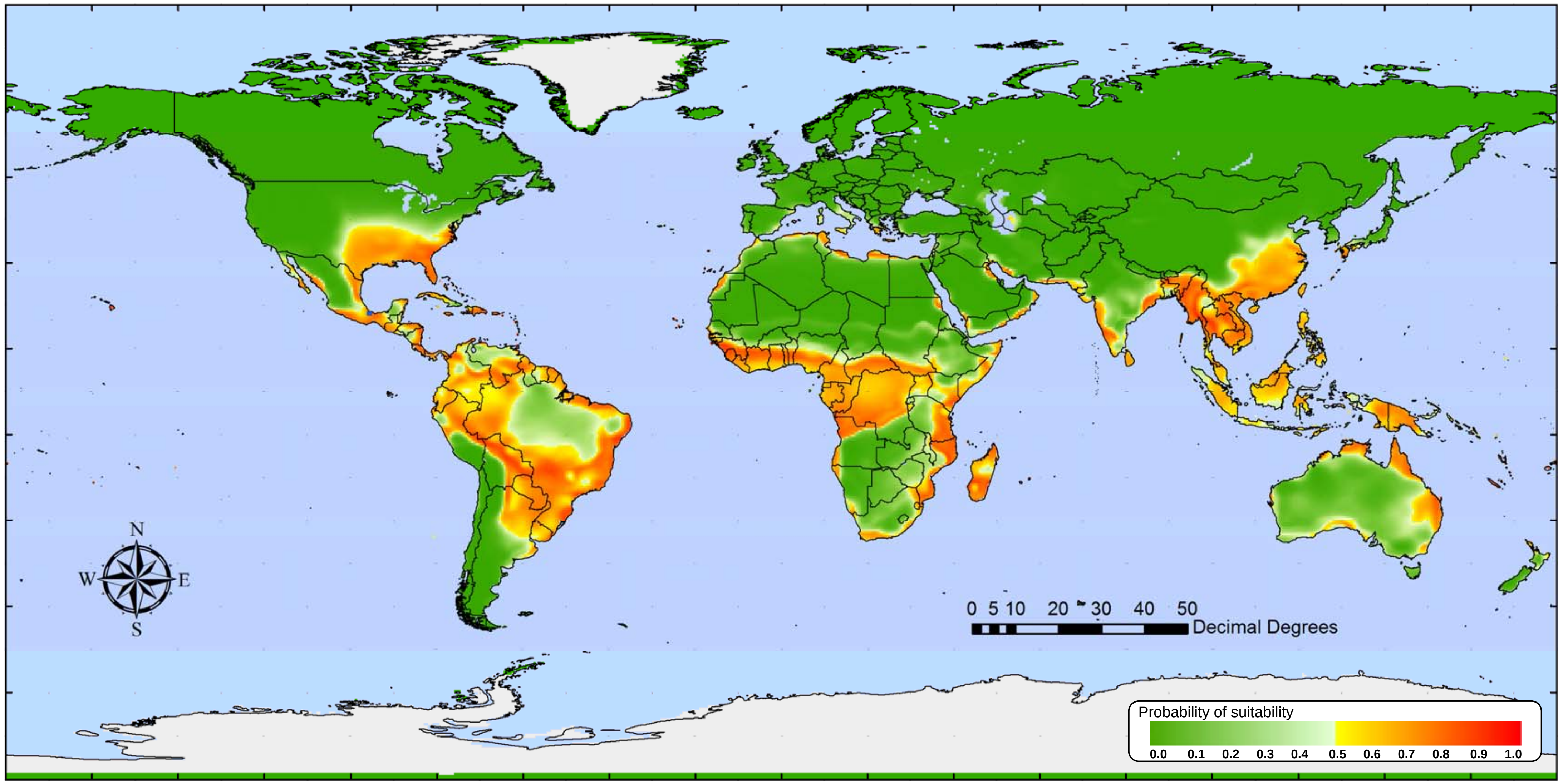

Figure 4, Row a. *Aedes aegypti,* scenario A1F, Year 2040.

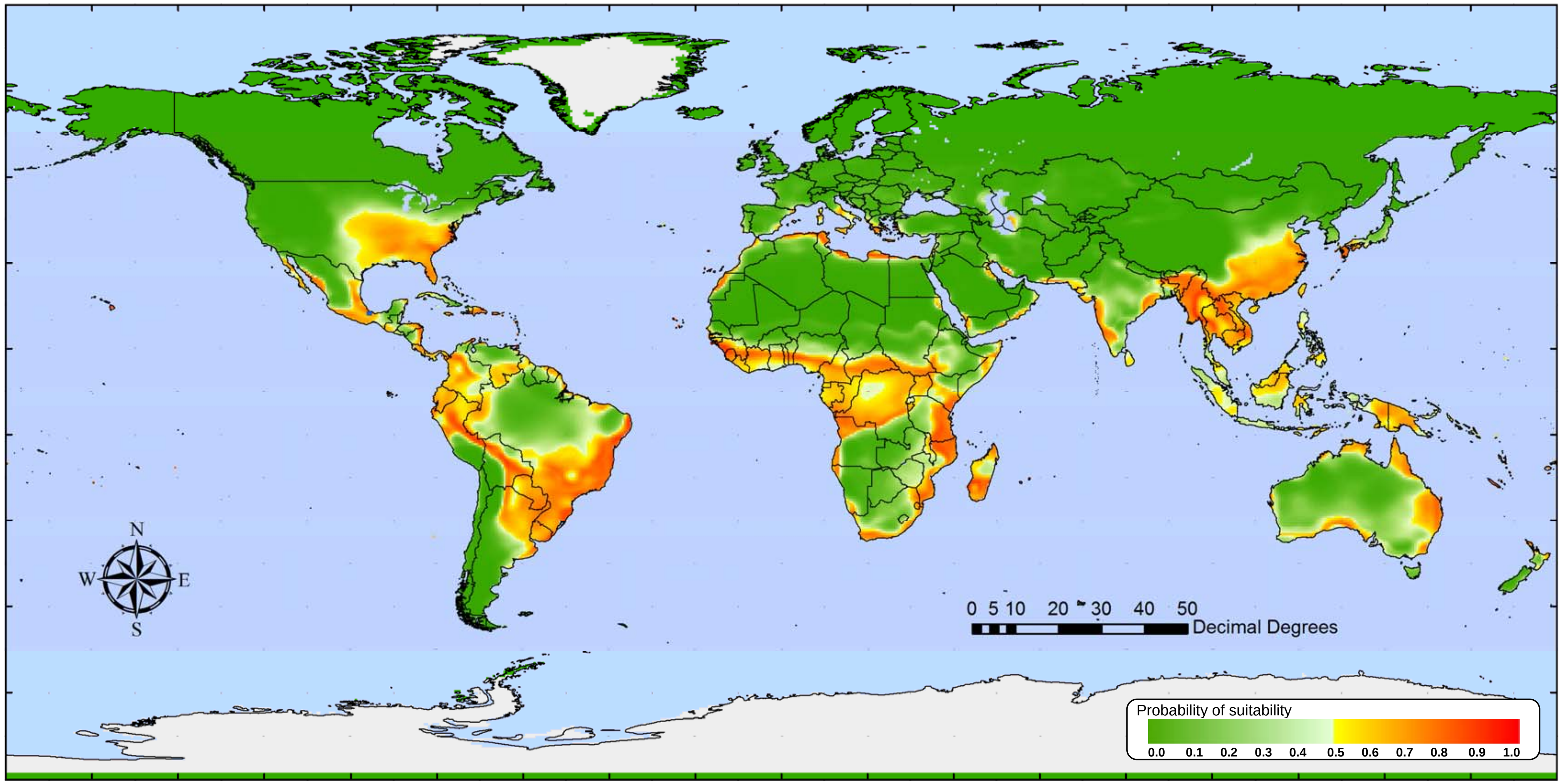

Figure 4, Row a. *Aedes aegypti,* scenario A1F, Year 2080.

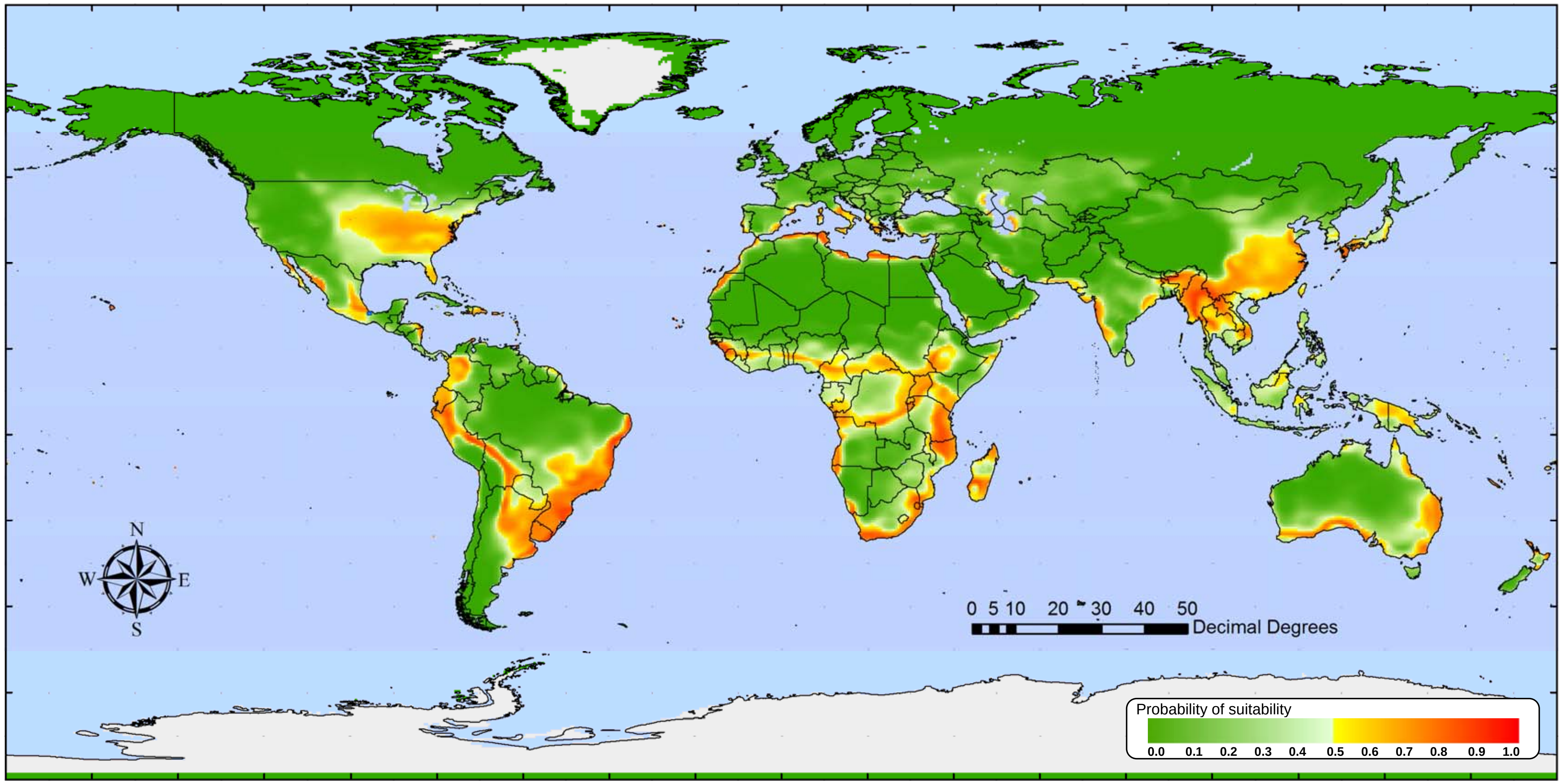

Figure 4, Row a. *Aedes aegypti,* scenario A1F, difference image (2080 - Present day). DECREASING suitability is in red in this image and INCREASING suitability in green.

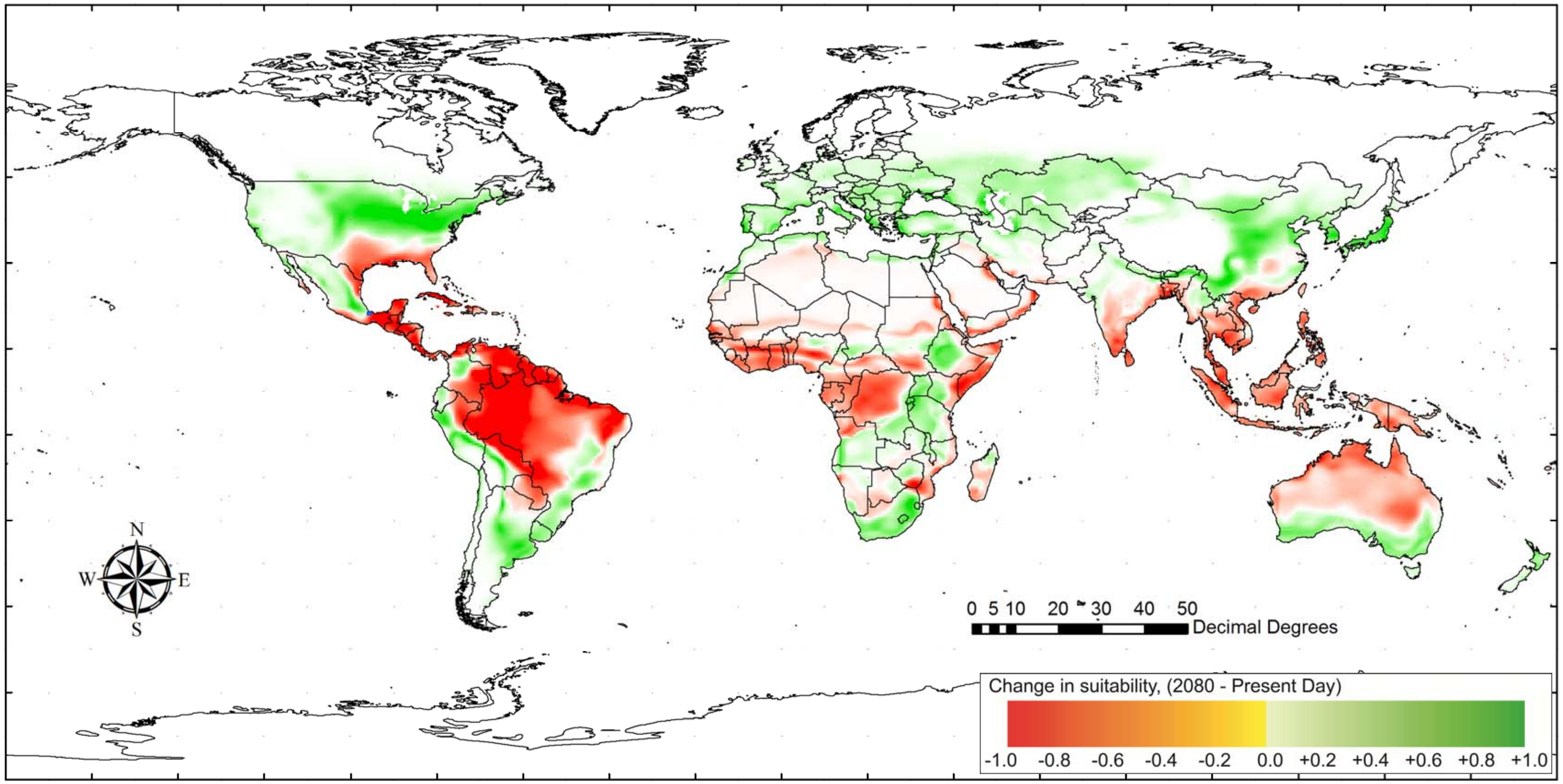

Figure 4, Row b. *Aedes albopictus,* scenario A1F, Present day.

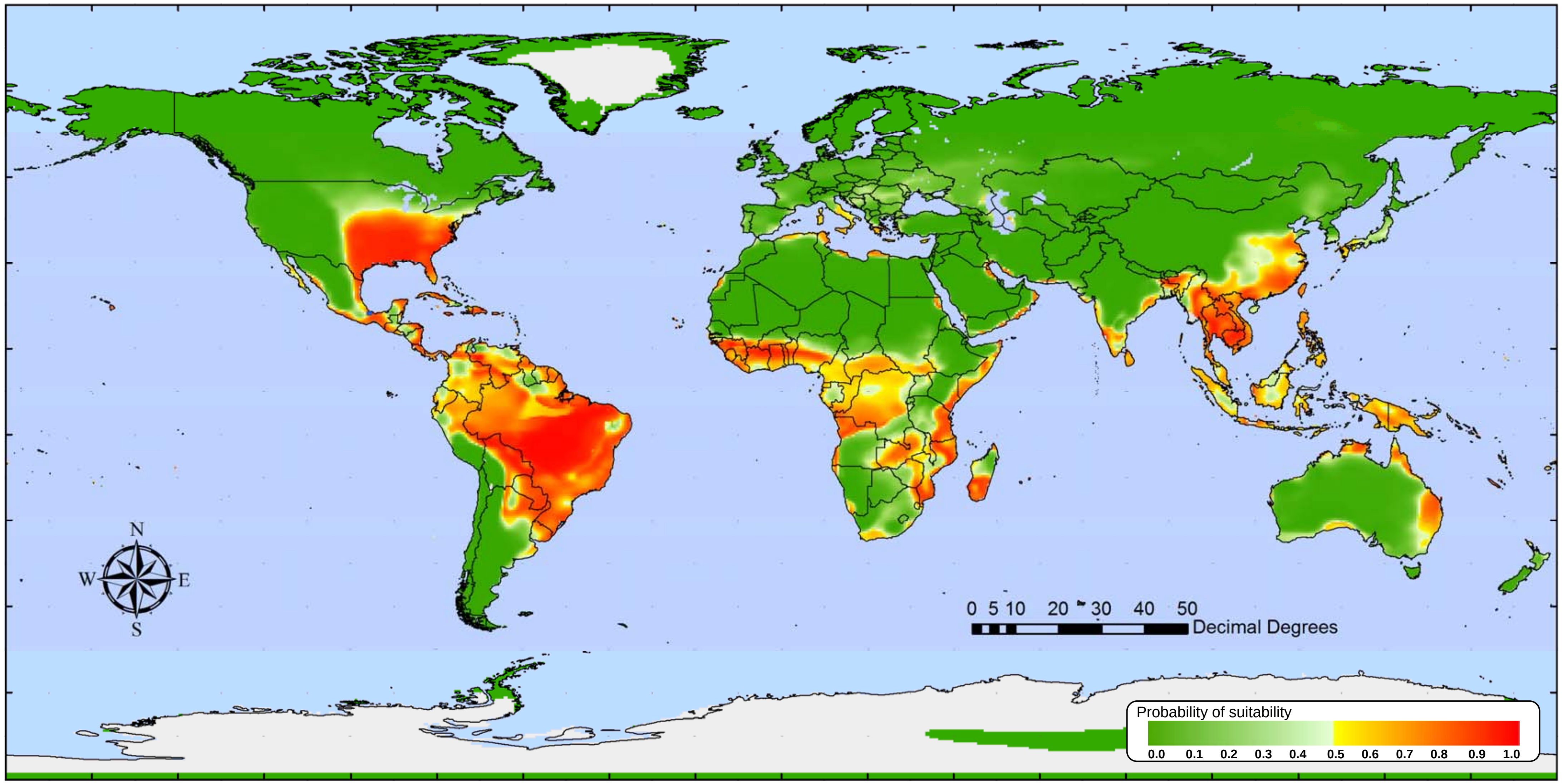

Figure 4, Row b. *Aedes albopictus,* scenario A1F, Year 2020.

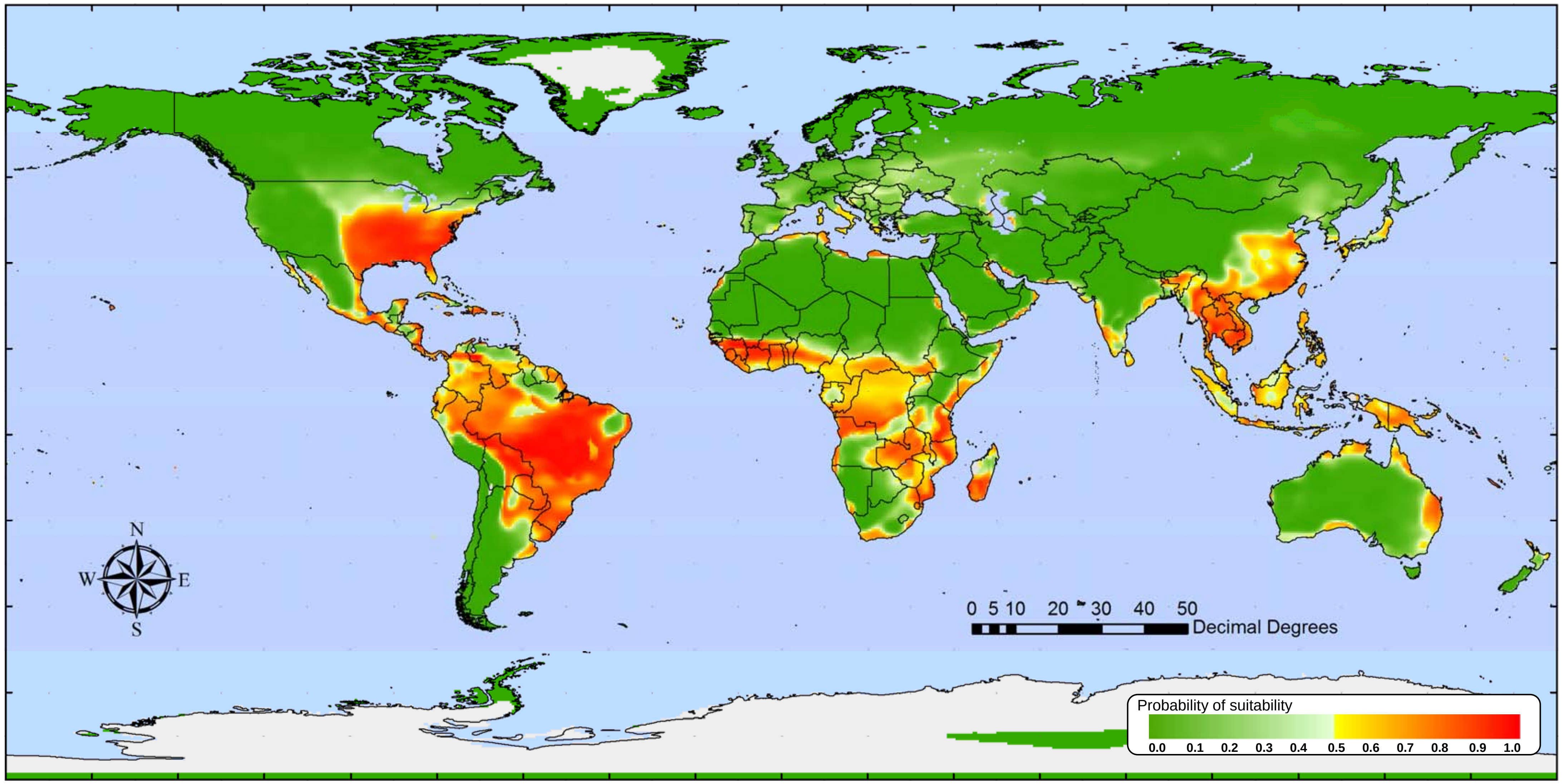

Figure 4, Row b. *Aedes albopictus,* scenario A1F, Year 2040.

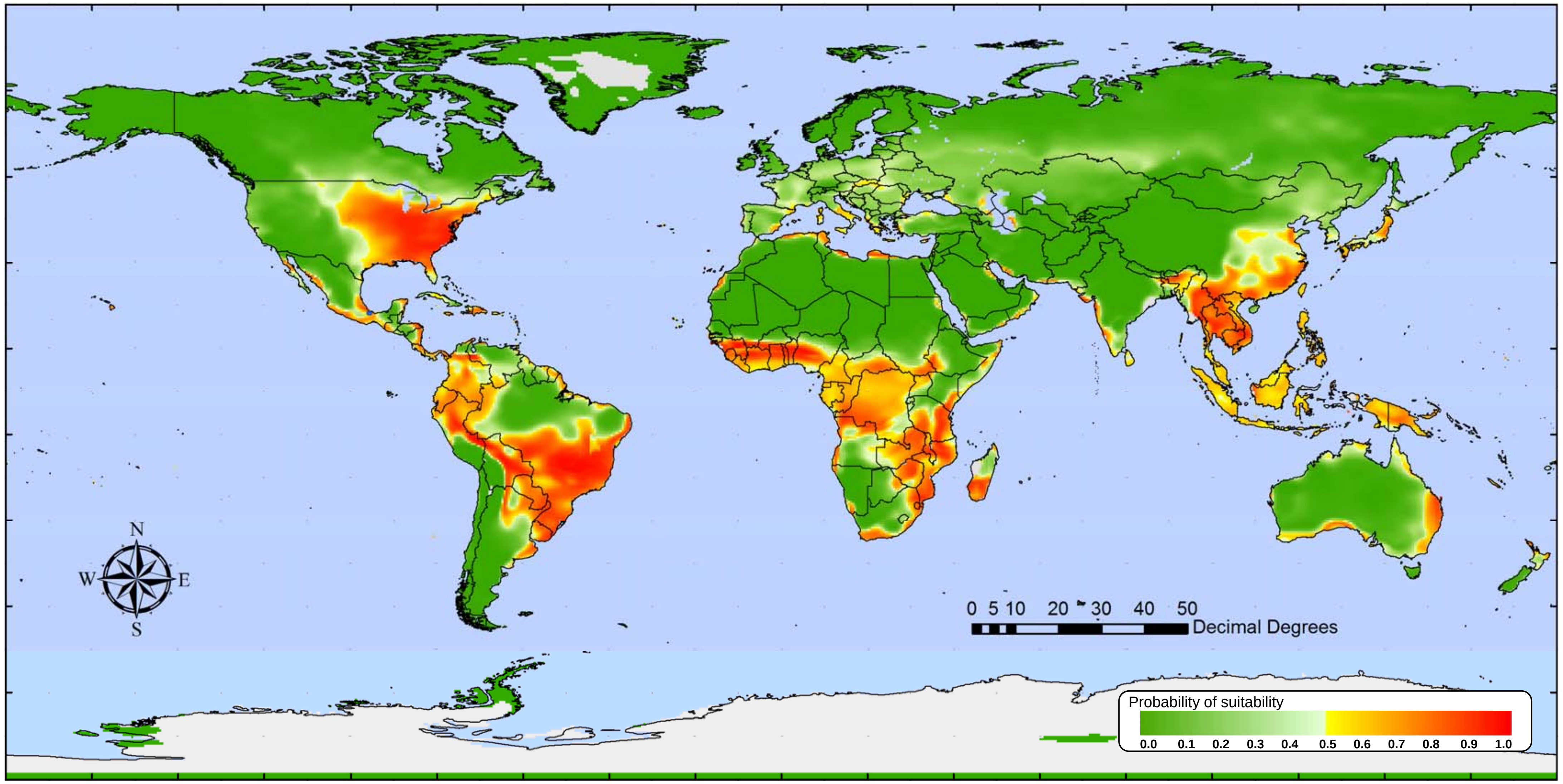

Figure 4, Row b. *Aedes albopictus,* scenario A1F, Year 2080.

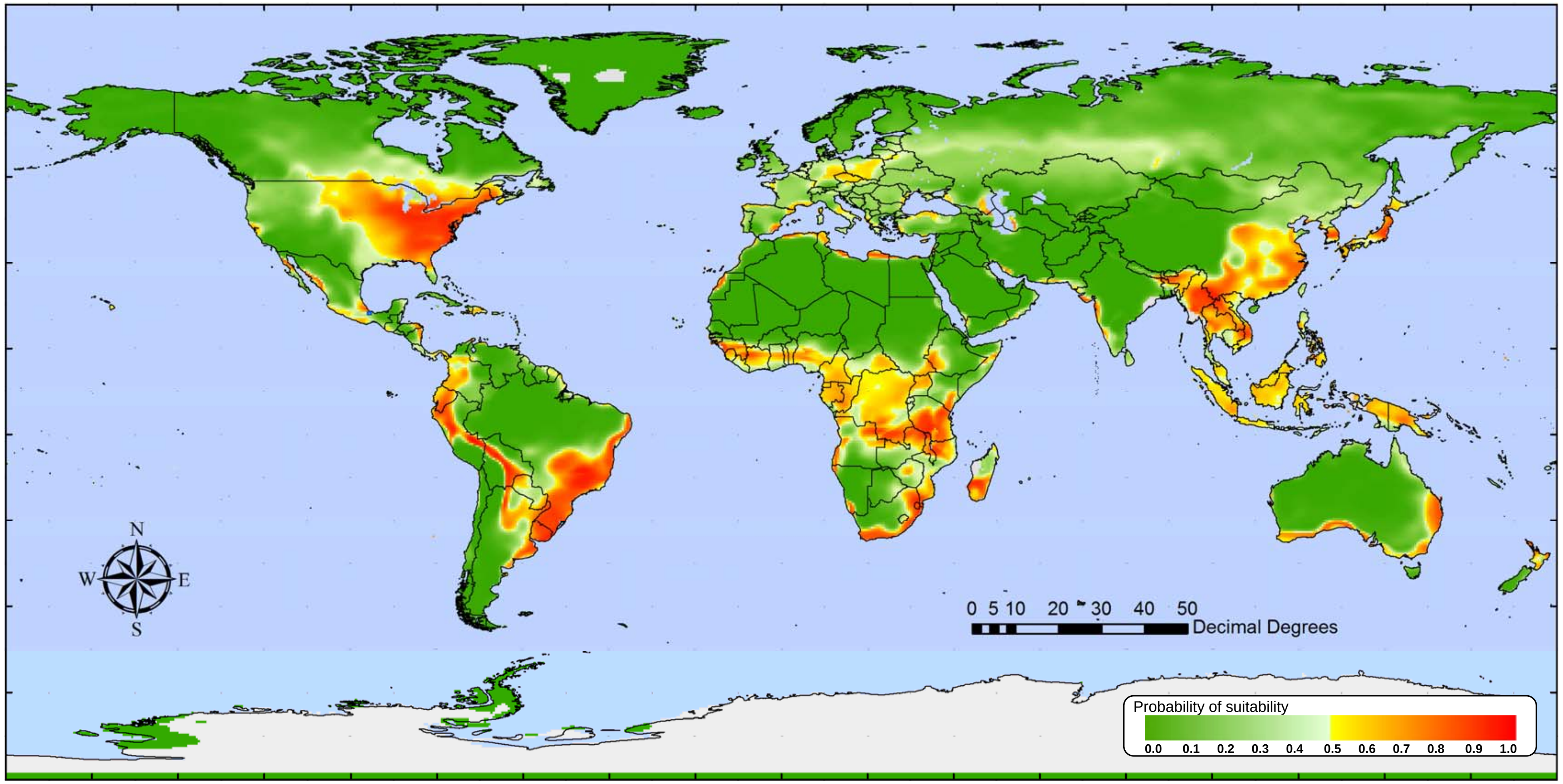

Figure 4, Row b. *Aedes albopictus,* scenario A1F, difference image (2080 - Present day). DECREASING suitability is in red in this image and INCREASING suitability in green.

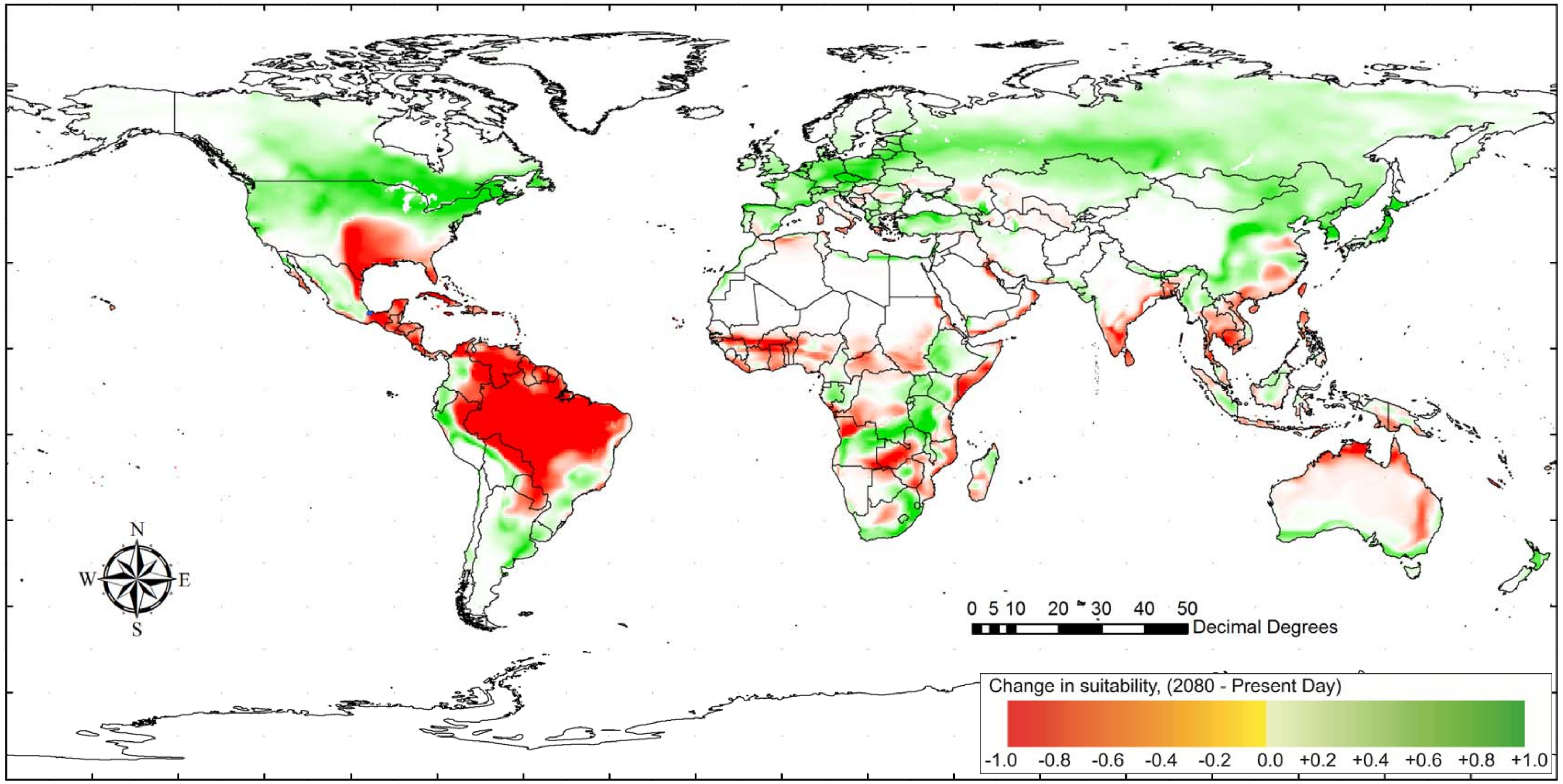

Figure 4, Row c. Dengue without vectors, scenario A1F, Present day.

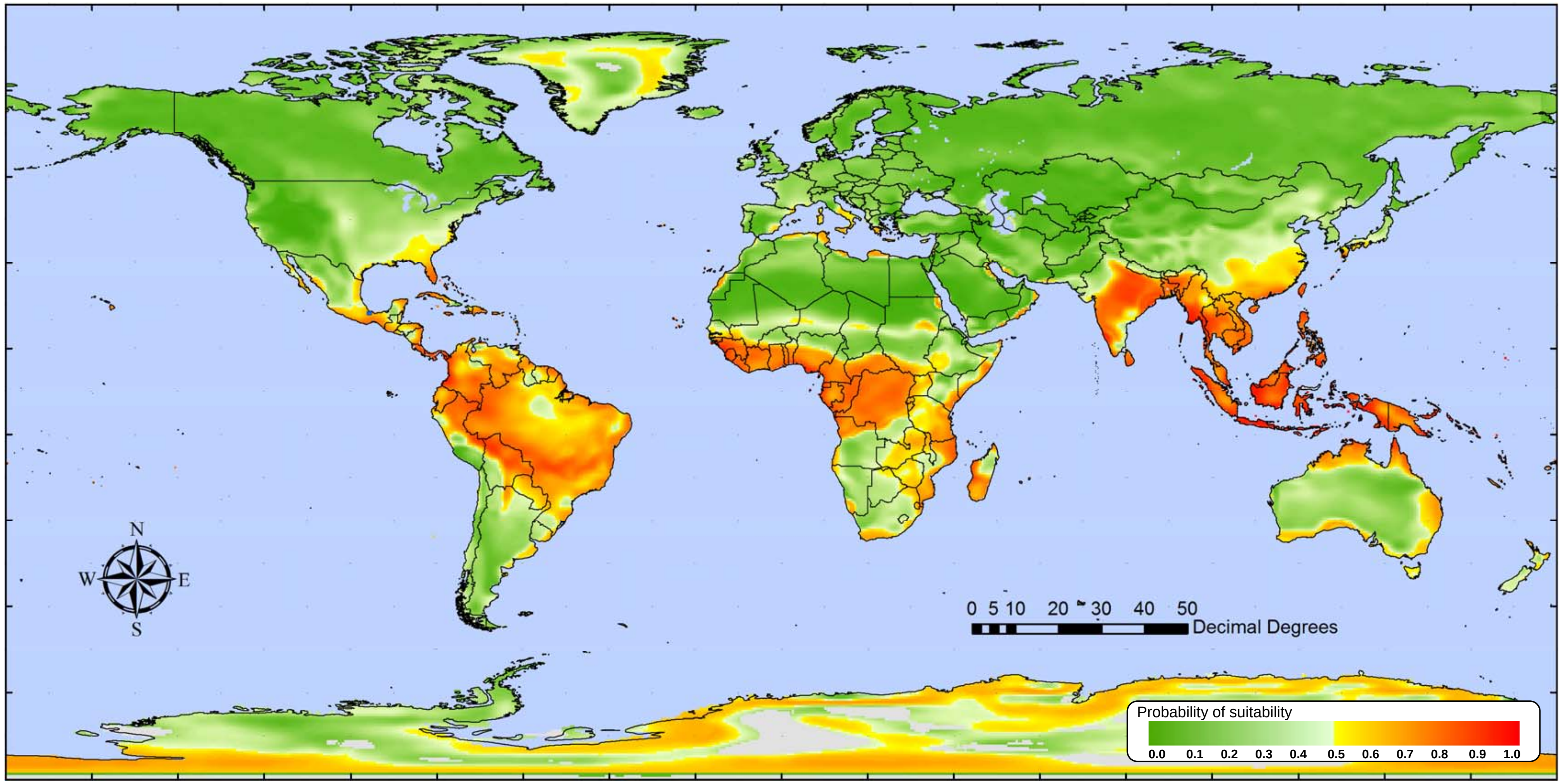

Figure 4, Row c. Dengue without vectors, scenario A1F, Year 2020.

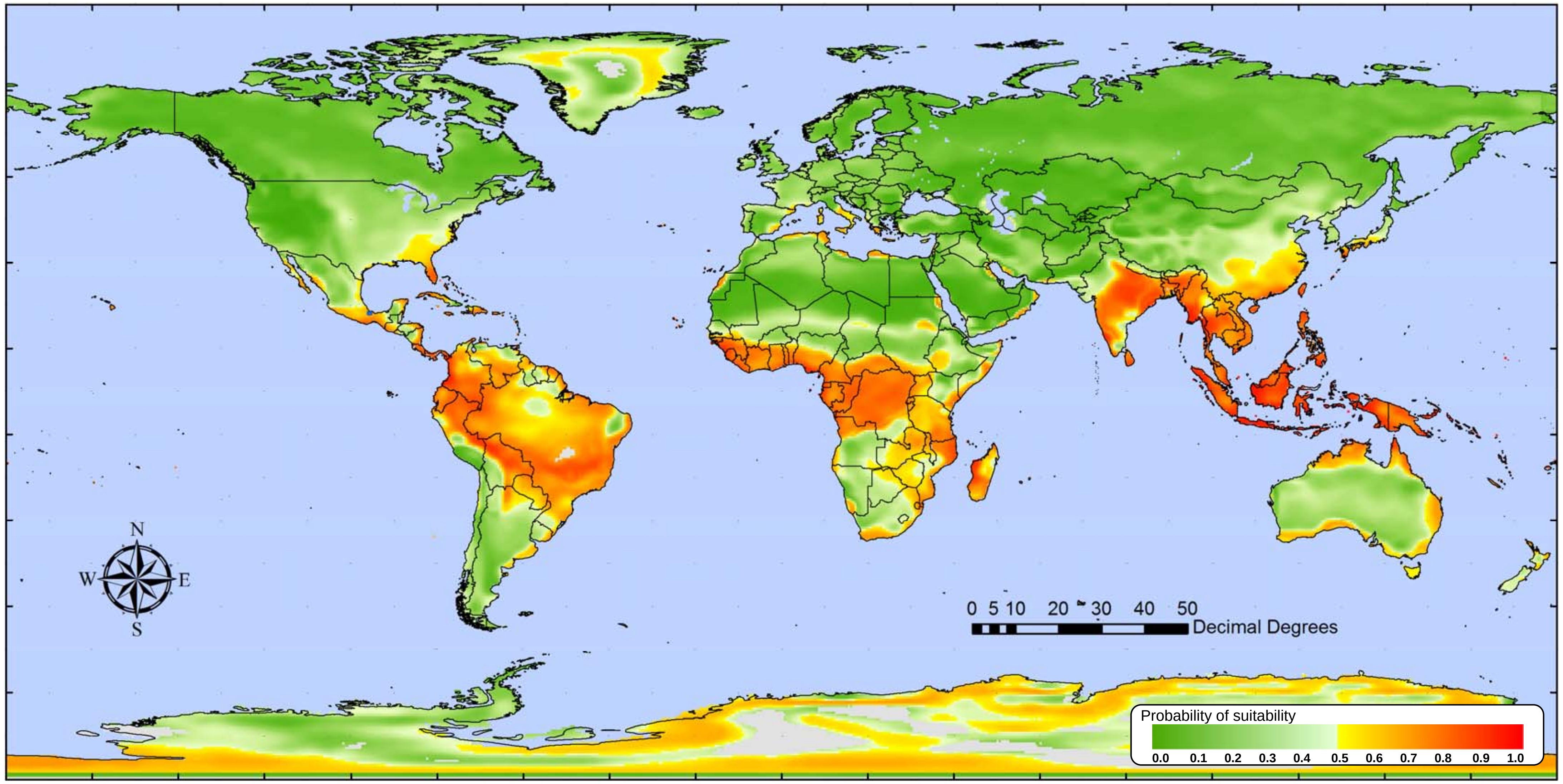

Figure 4, Row c. Dengue without vectors, scenario A1F, Year 2040.

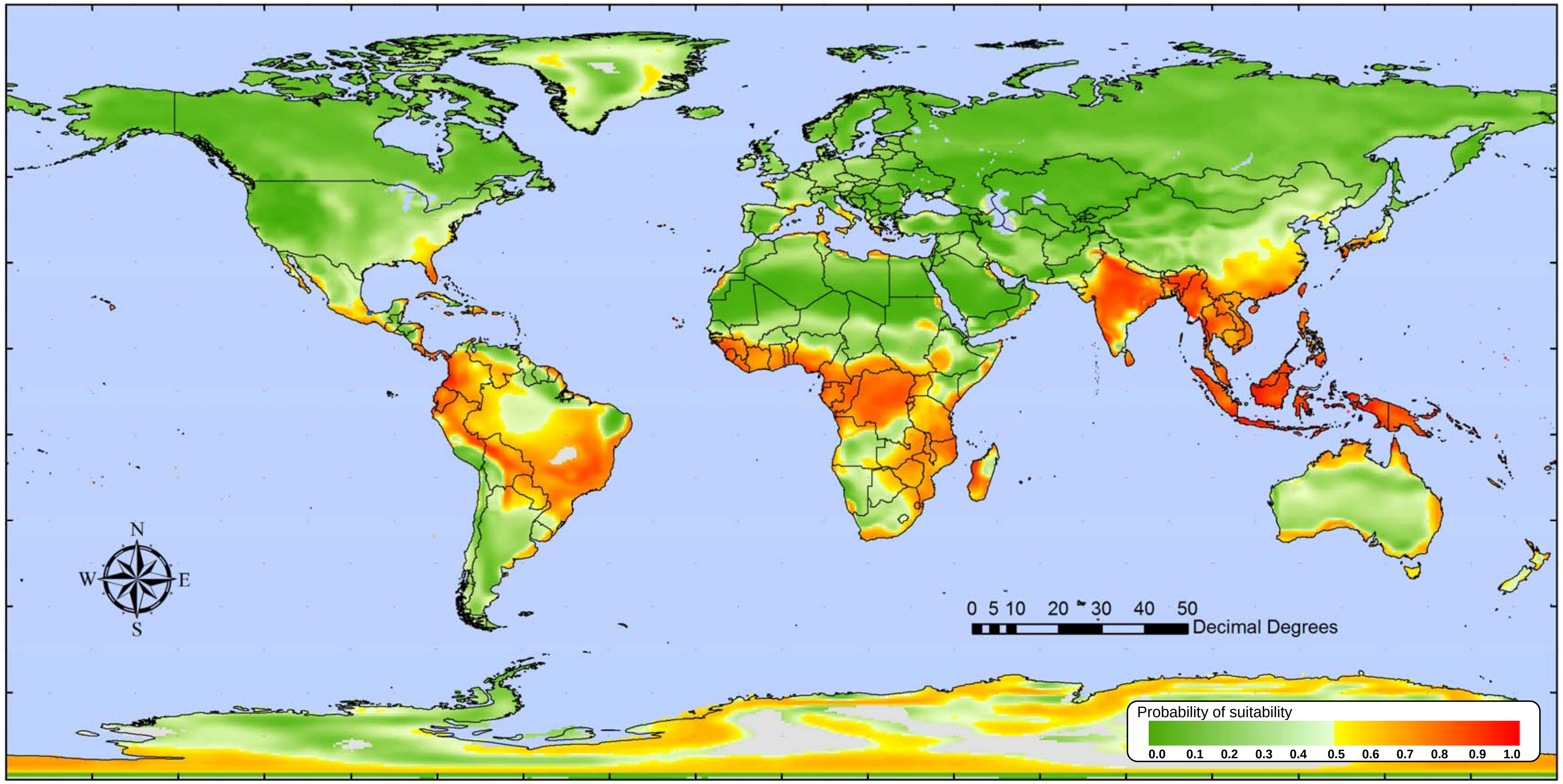

Figure 4, Row c. Dengue without vectors, scenario A1F, Year 2080.

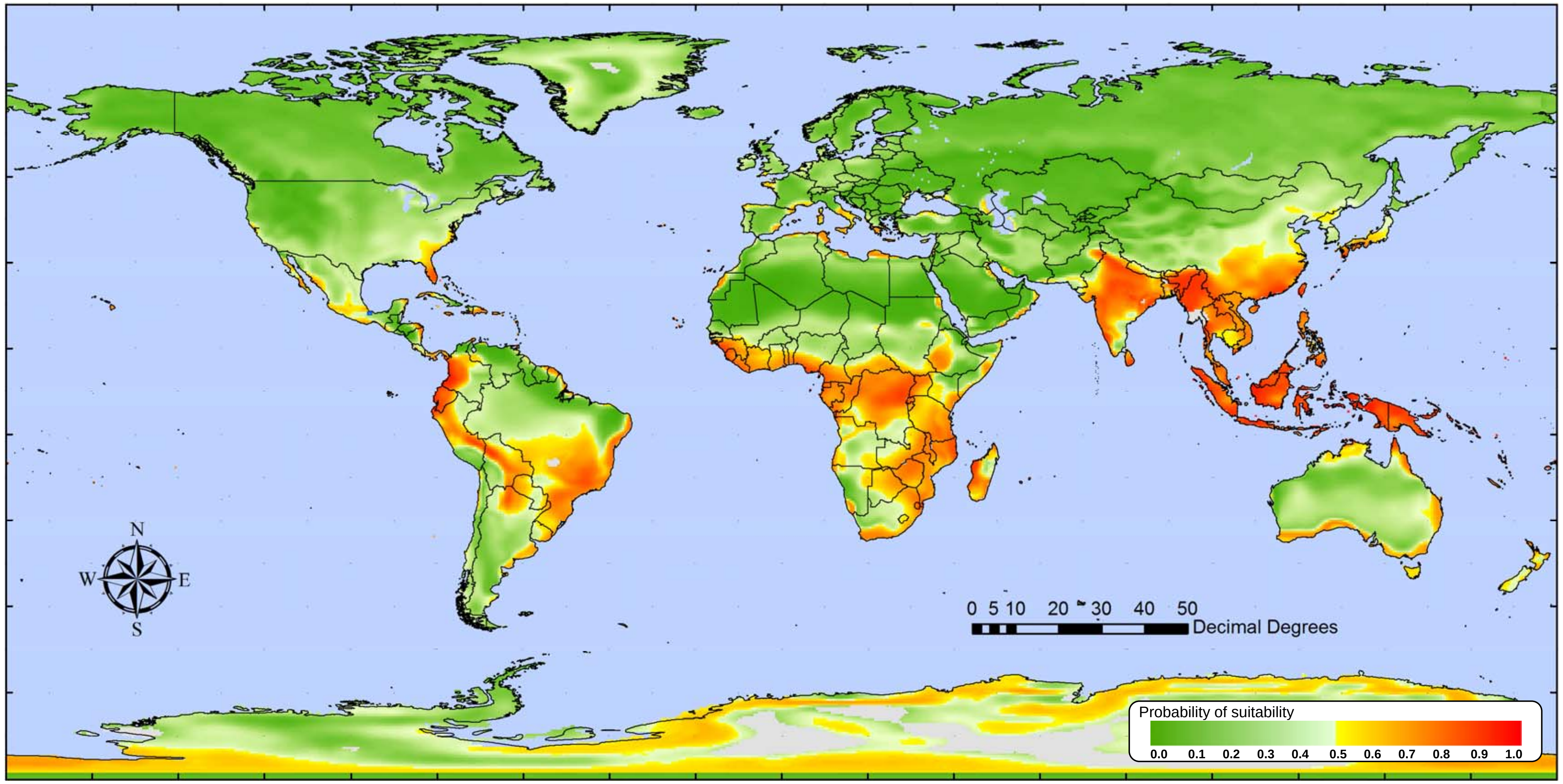

Figure 4, Row c. Dengue without vectors, scenario A1F, difference image (2080 - Present day). DECREASING suitability is in red in this image and INCREASING suitability in green.

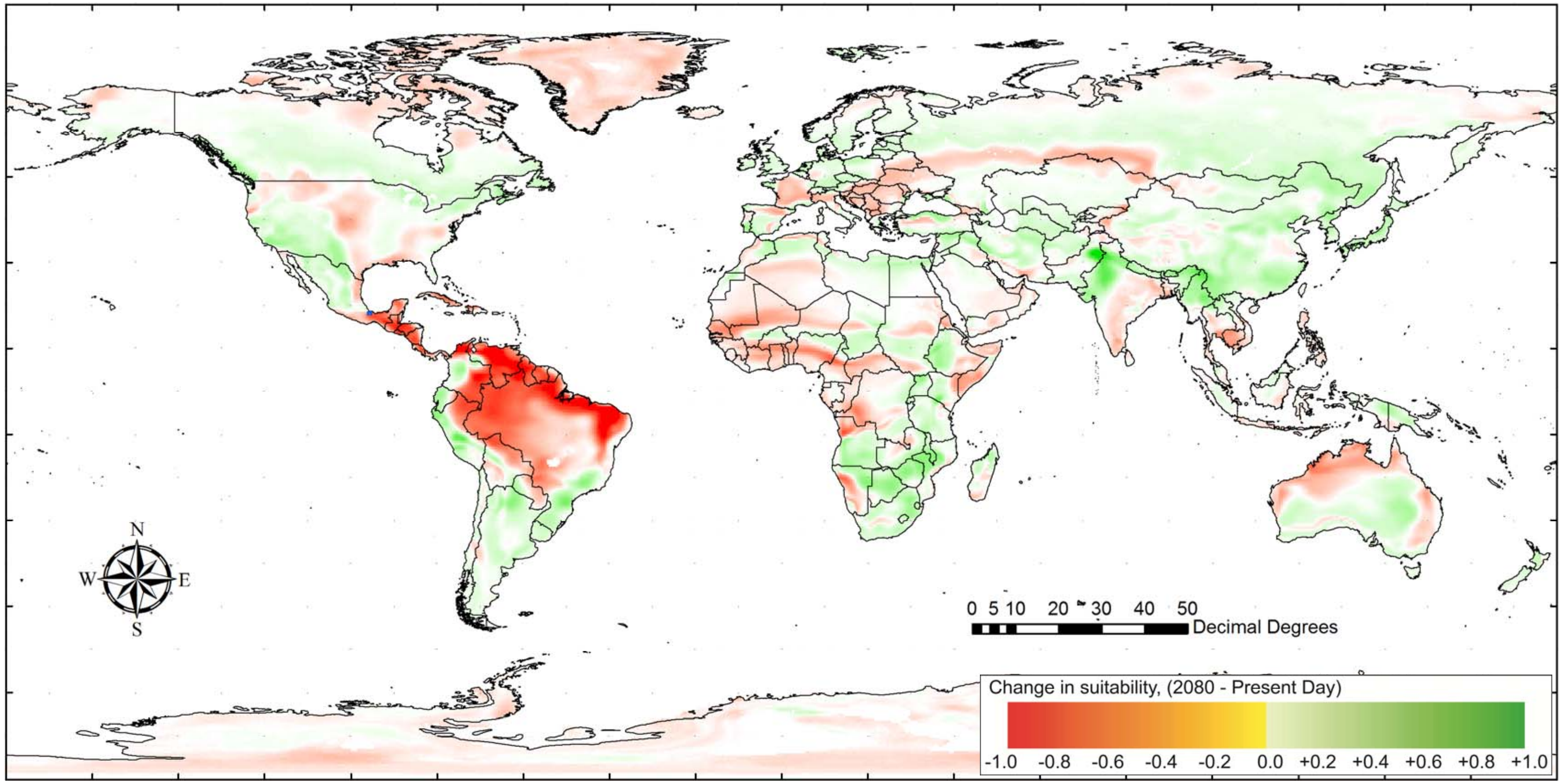

Figure 4, Row d. Dengue with vectors, scenario A1F, Present day.

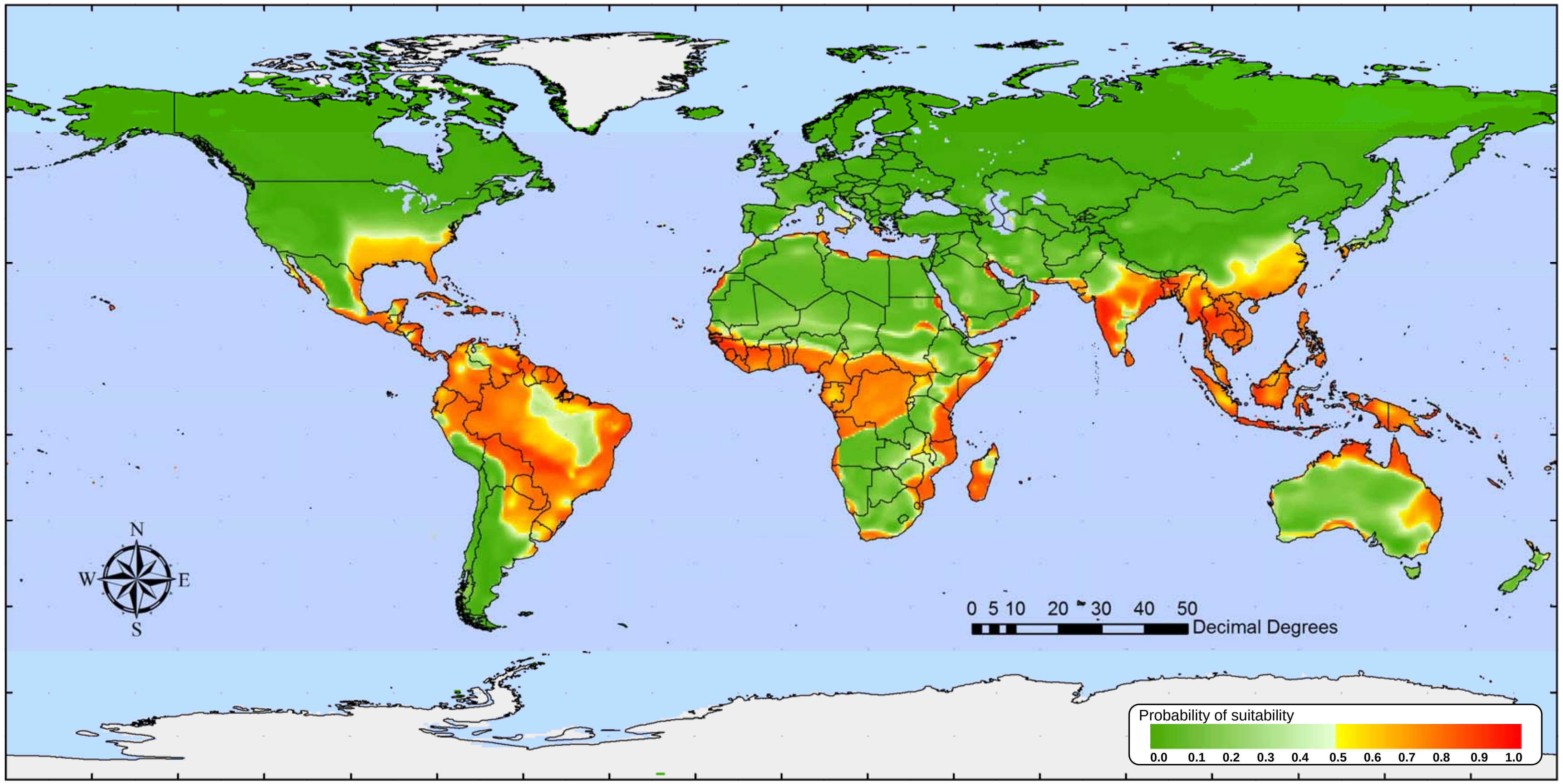

Figure 4, Row d. Dengue with vectors, scenario A1F, Year 2020.

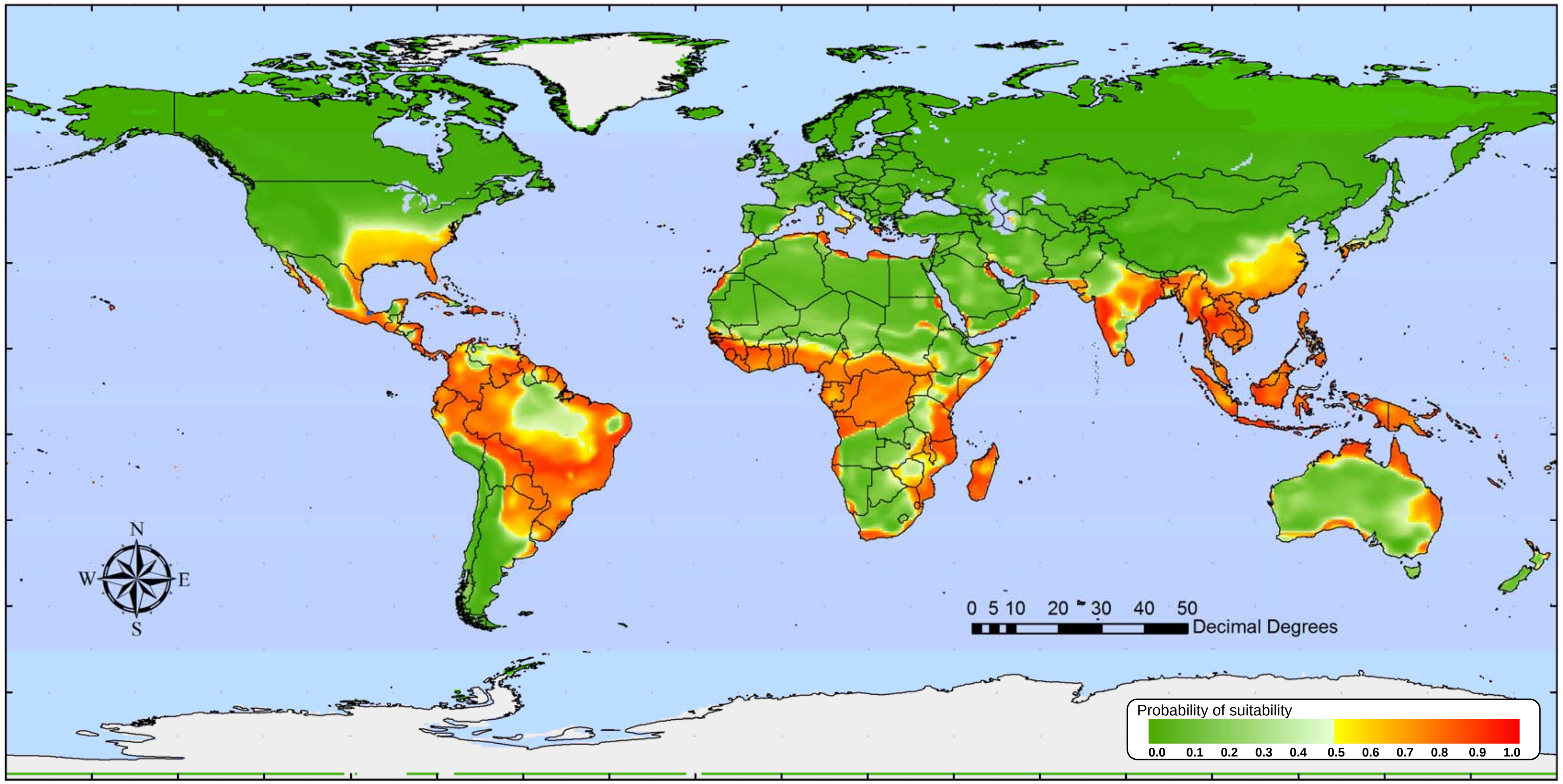

Figure 4, Row d. Dengue with vectors, scenario A1F, Year 2040.

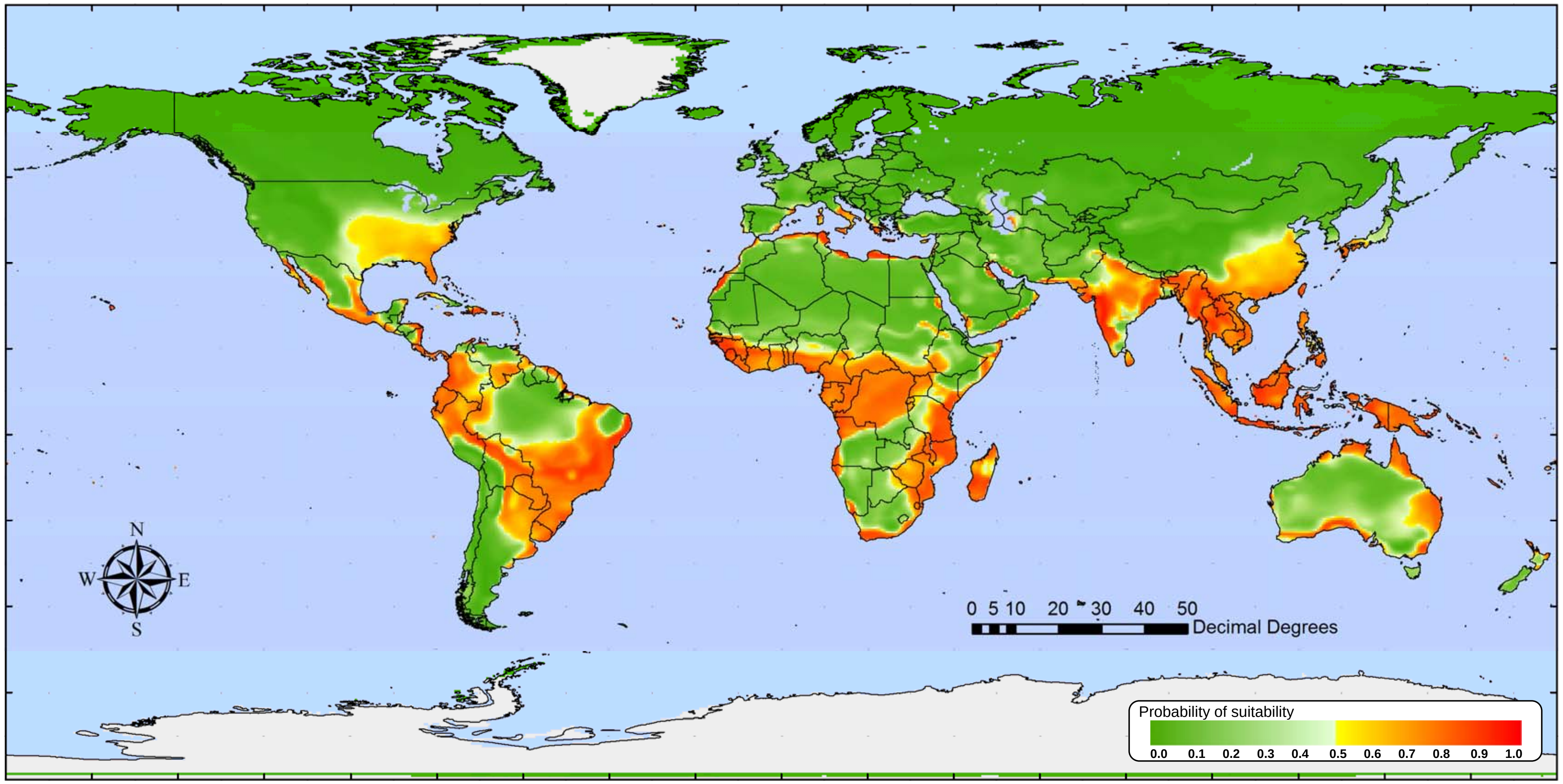

Figure 4, Row d. Dengue with vectors, scenario A1F, Year 2080.

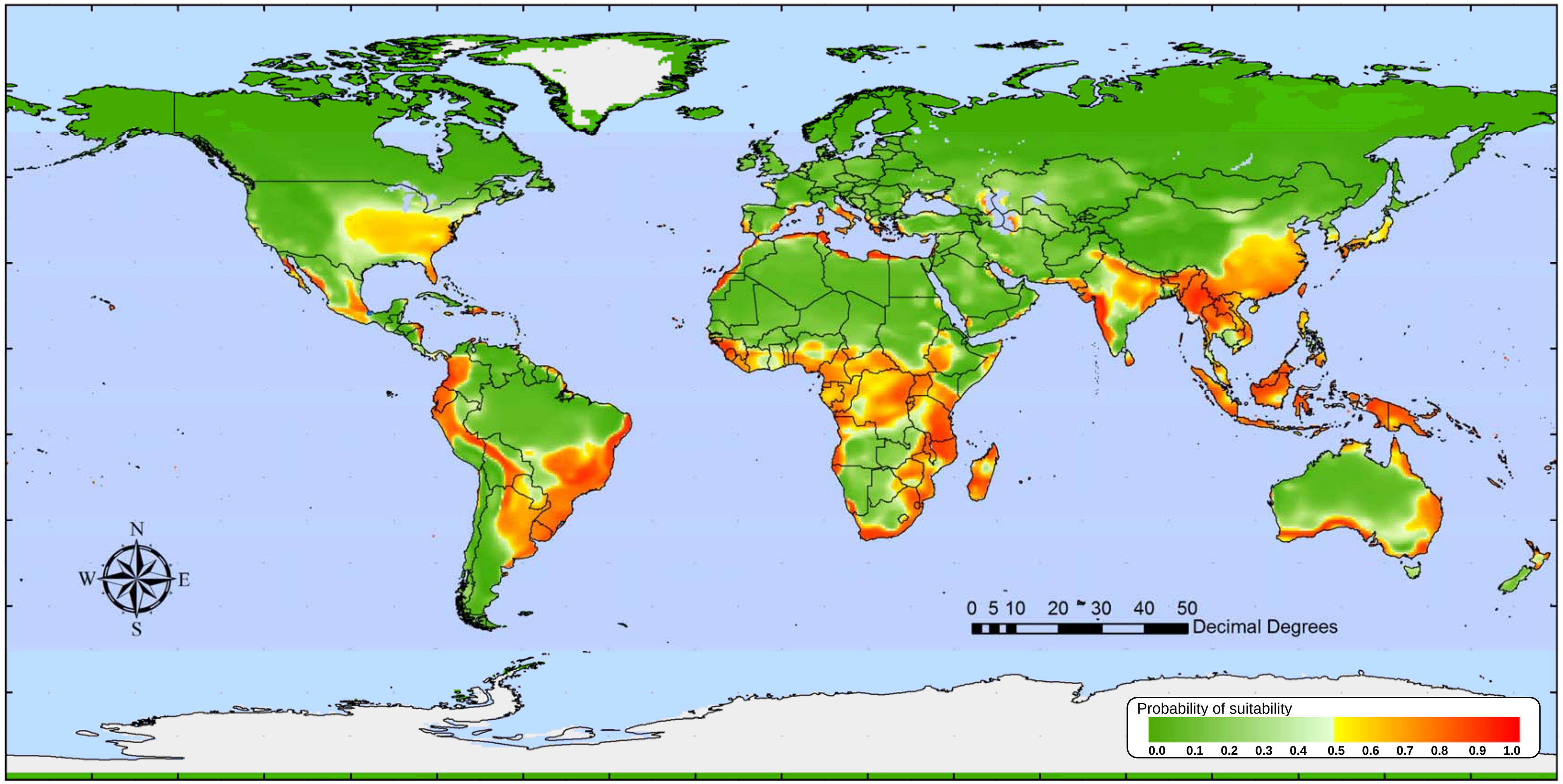

Figure 4, Row d. Dengue with vectors, scenario A1F, difference image (2080 - Present day). DECREASING suitability is in red in this image and INCREASING suitability in green.

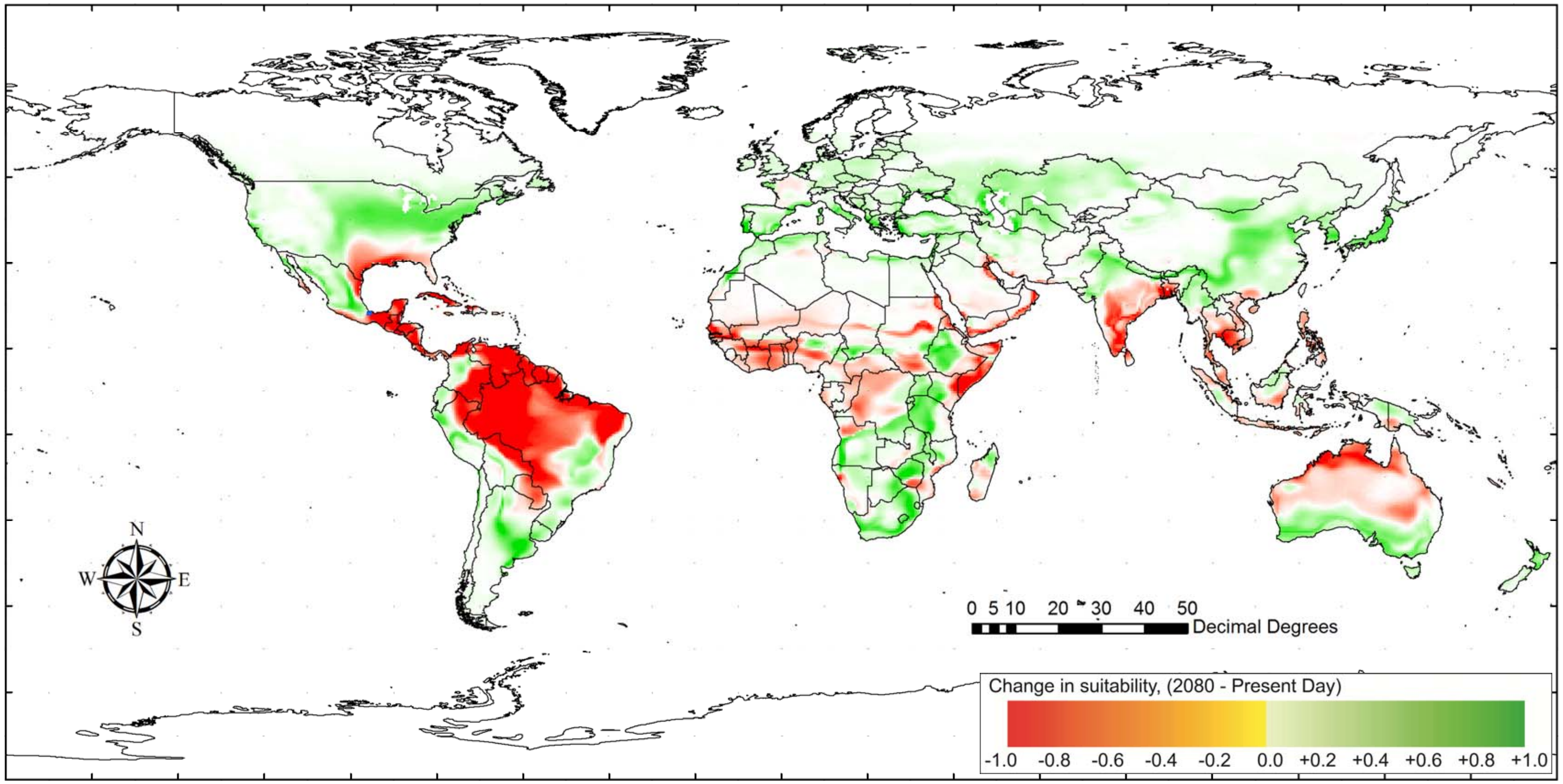

Figure 5a. Dengue with vectors, scenario A1F, Present day with a reduced set of (less correlated) predictor variables.

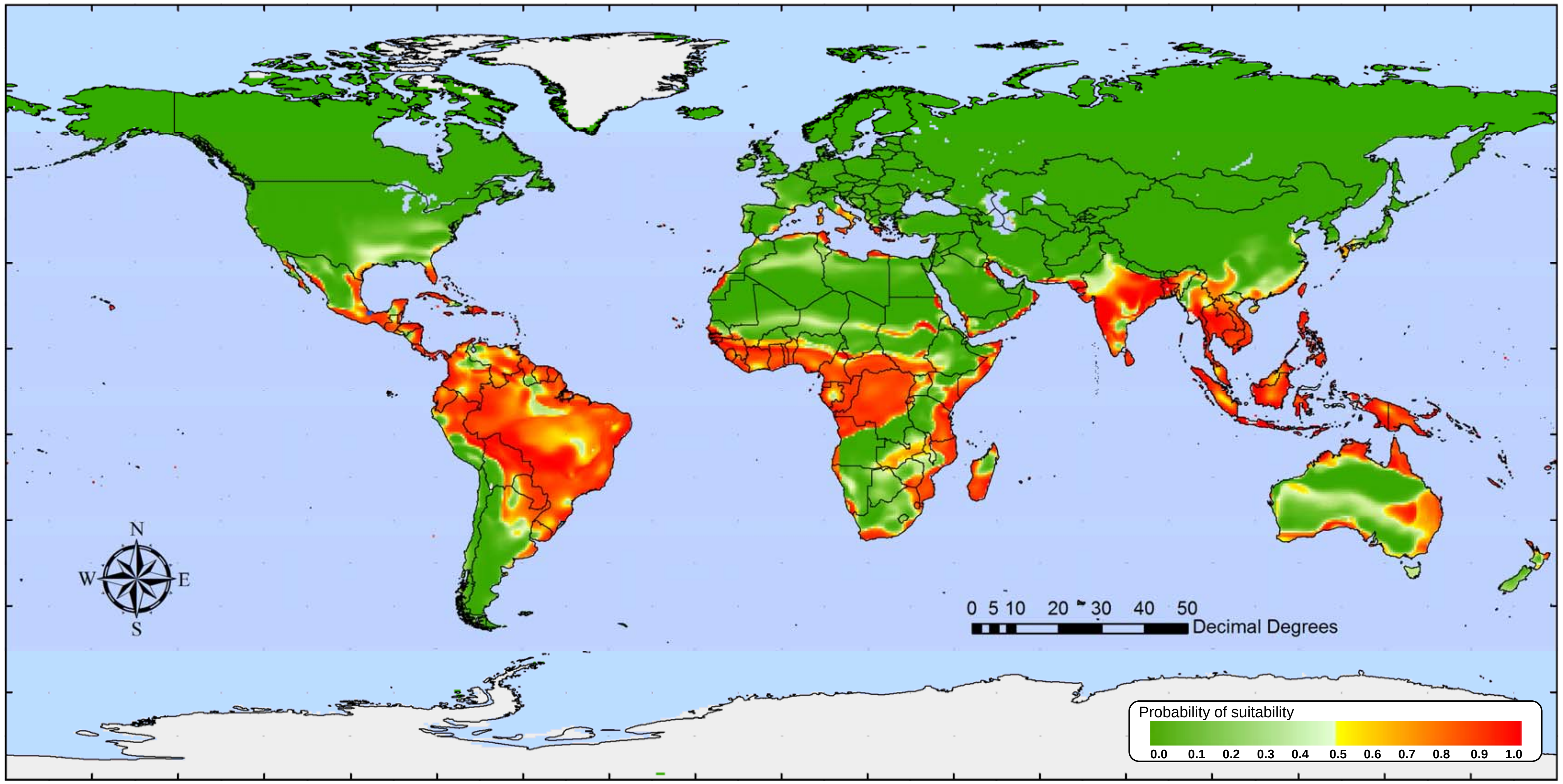

Figure 5b Dengue with vectors, scenario A1F, Year 2080 with a reduced set of (less correlated) predictor variables.

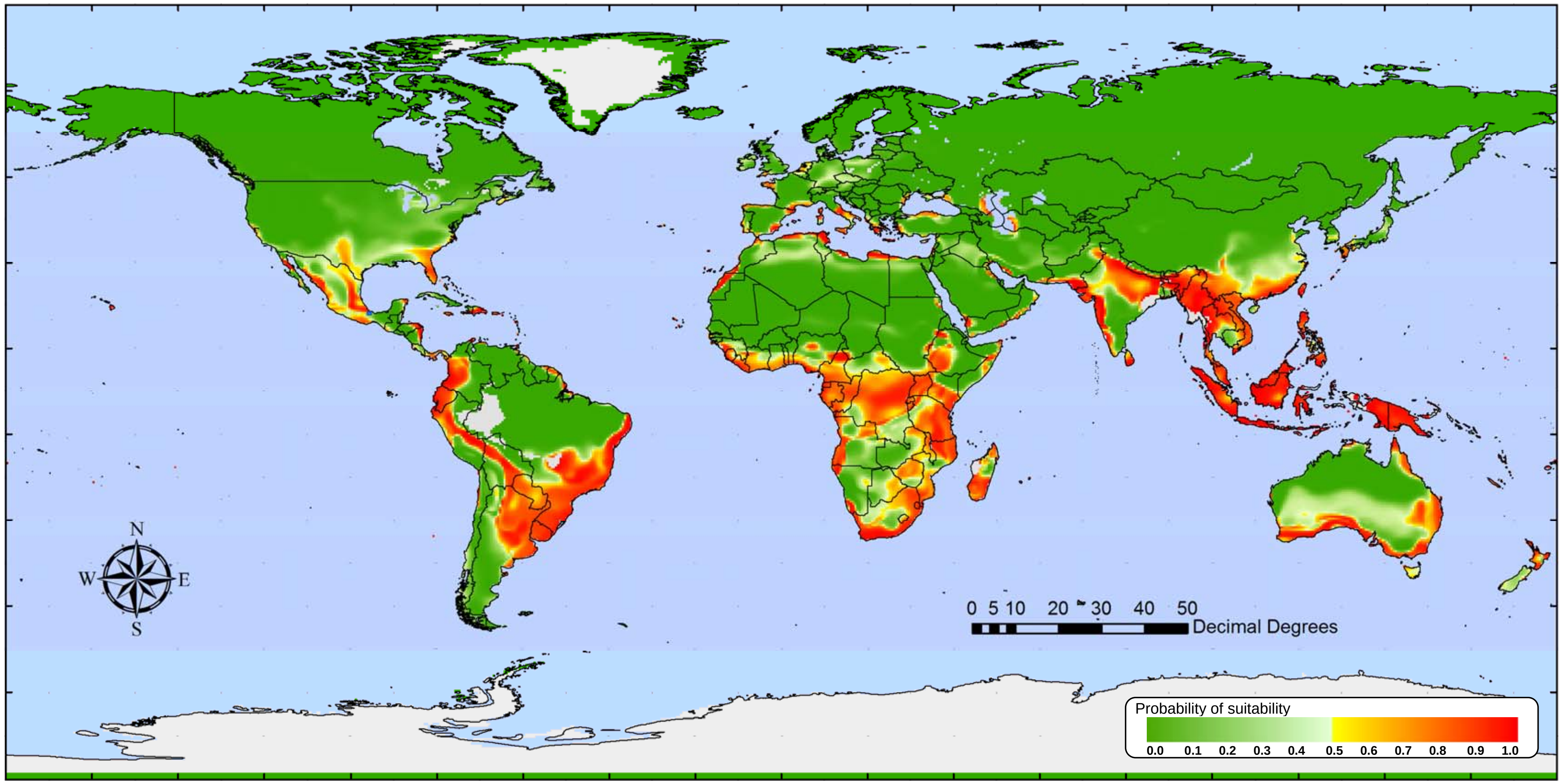

Figure 5c Difference between Fig 5a and 5b, (2080 - Present day). DECREASING suitability is in red in this image and INCREASING suitability in green.

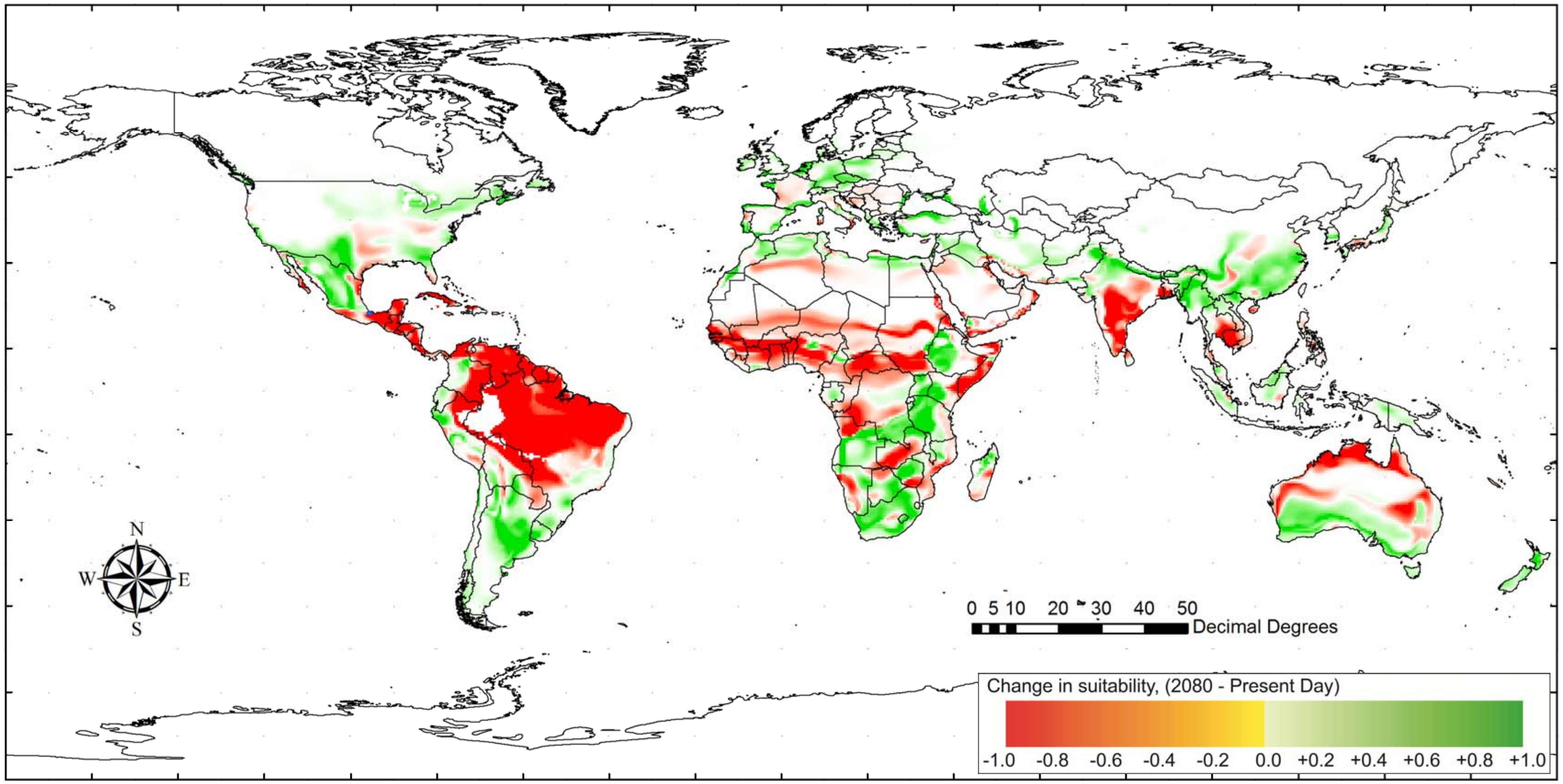

Figure 5d Corr-max image from the present day dengue model in figure 5a (i.e. Present day). See legend for the identification of key variables determining the Mahalanobis Distances to the nearest (in environmental space) presence cluster. Differences may be positive (indicating that present day values are higher than those of the relevant cluster centroid) or negative (indicating present day values are lower than the relevant cluster centroid).

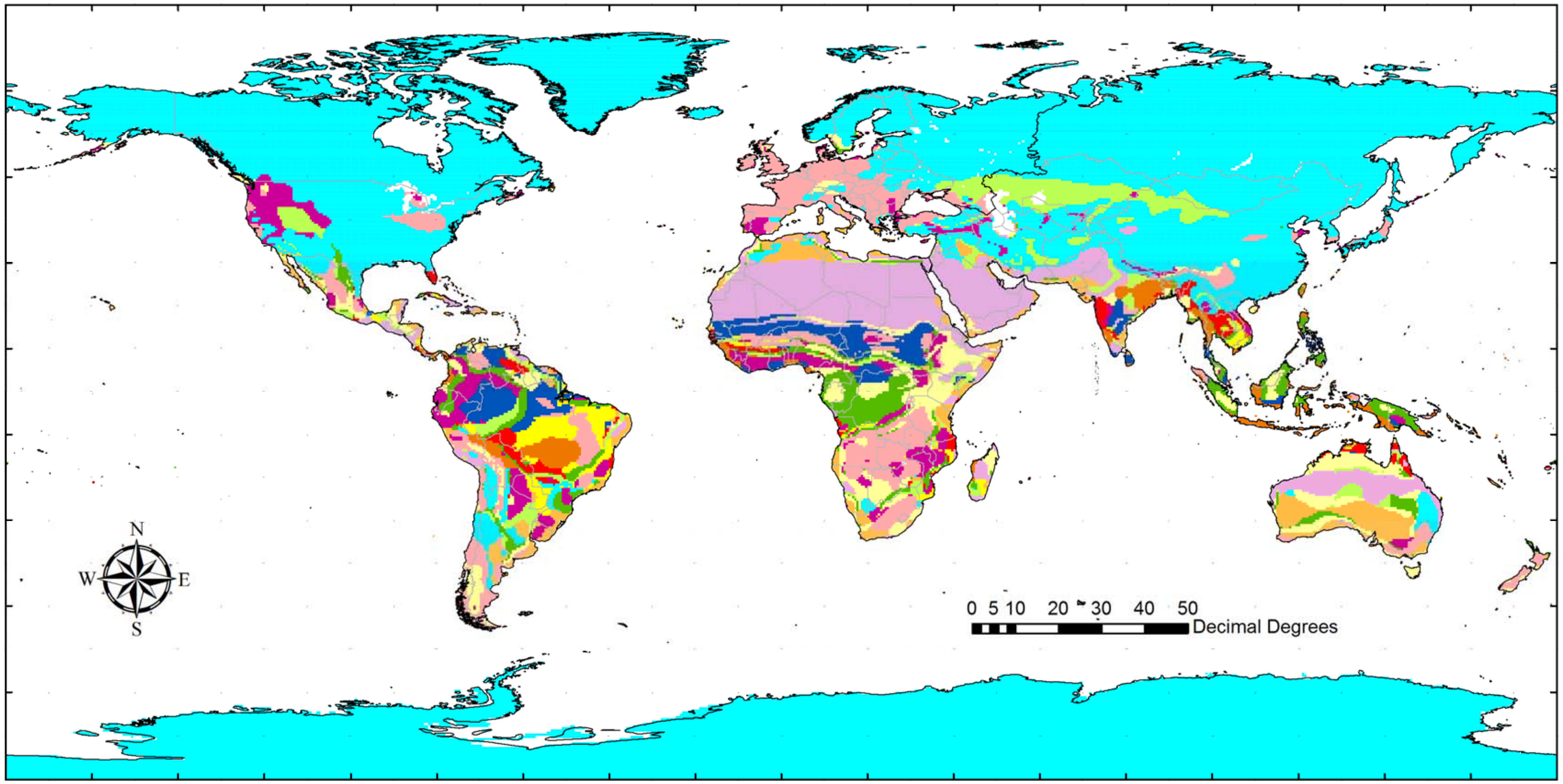

Corr-max image

- -6 Relative Humidity max.
- -5 Temperature min.
- -4 Relative Humidity min.
- -3 Aedes albopictus
- -2 Precipitation max.
- -1 Aedes aegypti
- +1 Aedes aegypti
- +2 Precipitation max.
- +3 Aedes albopictus
- +4 Relative Humidity min.
- +5 Temperature min.
- +6 Relative Humidity max.

Figure 5e. Corr-max image from the A1F Year 2080 dengue model in figure 5b (i.e. for the year 2080). See legend for the identification of key variables determining the Mahalanobis Distances to the nearest (in environmental space) presence cluster. Differences may be positive (indicating that values in 2080 are higher than those of the relevant cluster centroid defined by present day conditions) or negative (indicating that values in 2080 are lower than the relevant cluster centroid defined by present day conditions).

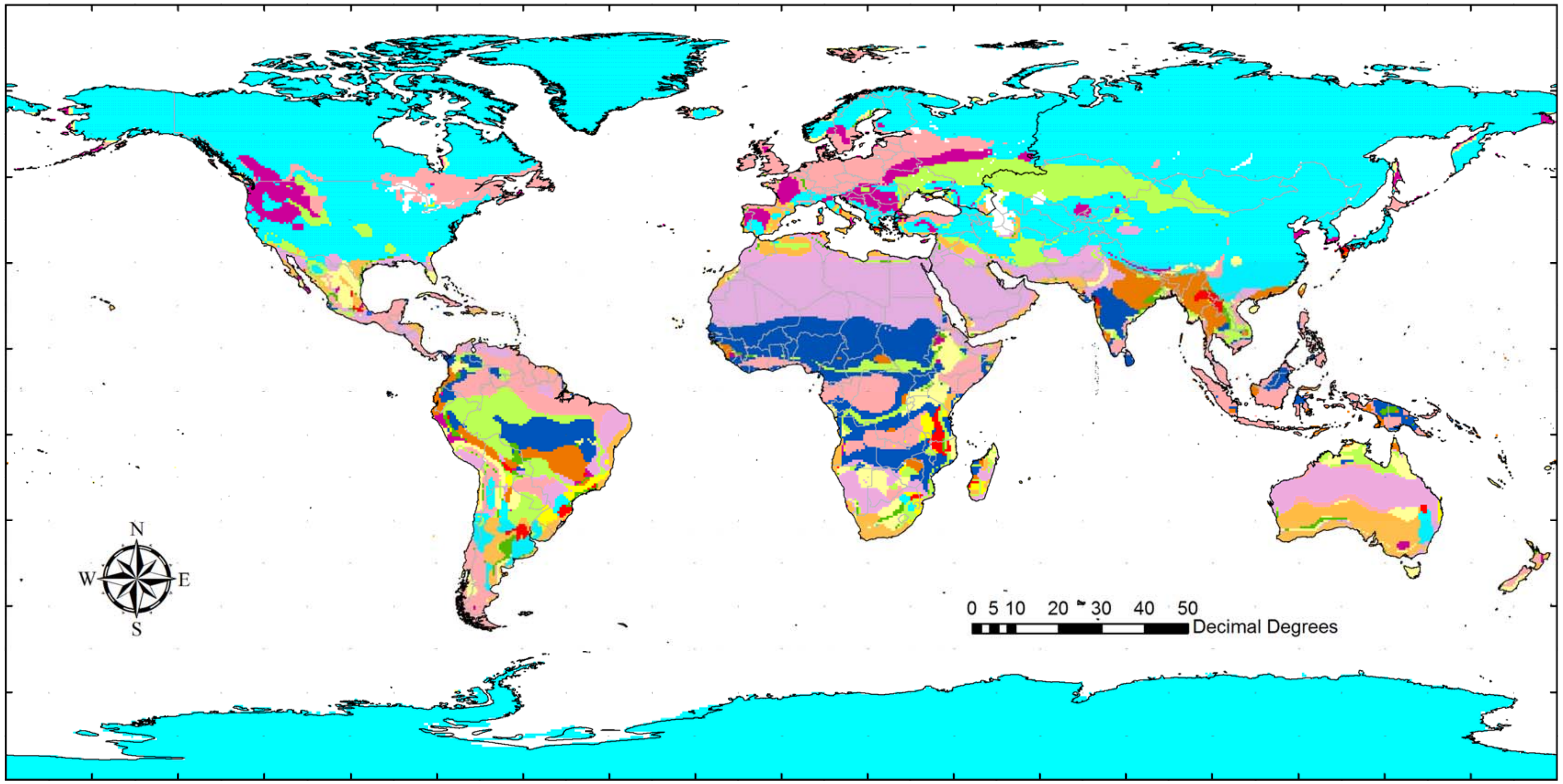

Corr-max image

- -6 Relative Humidity max.
- -5 Temperature min.
- -4 Relative Humidity min.
- -3 Aedes albopictus
- -2 Precipitation max.
- -1 Aedes aegypti
- +1 Aedes aegypti
- +2 Precipitation max.
- +3 Aedes albopictus
- +4 Relative Humidity min.
- +5 Temperature min.
- +6 Relative Humidity max.

Figure 5f. As figure 5d but only for areas predicted suitable for dengue at the present time (from figure 5a).

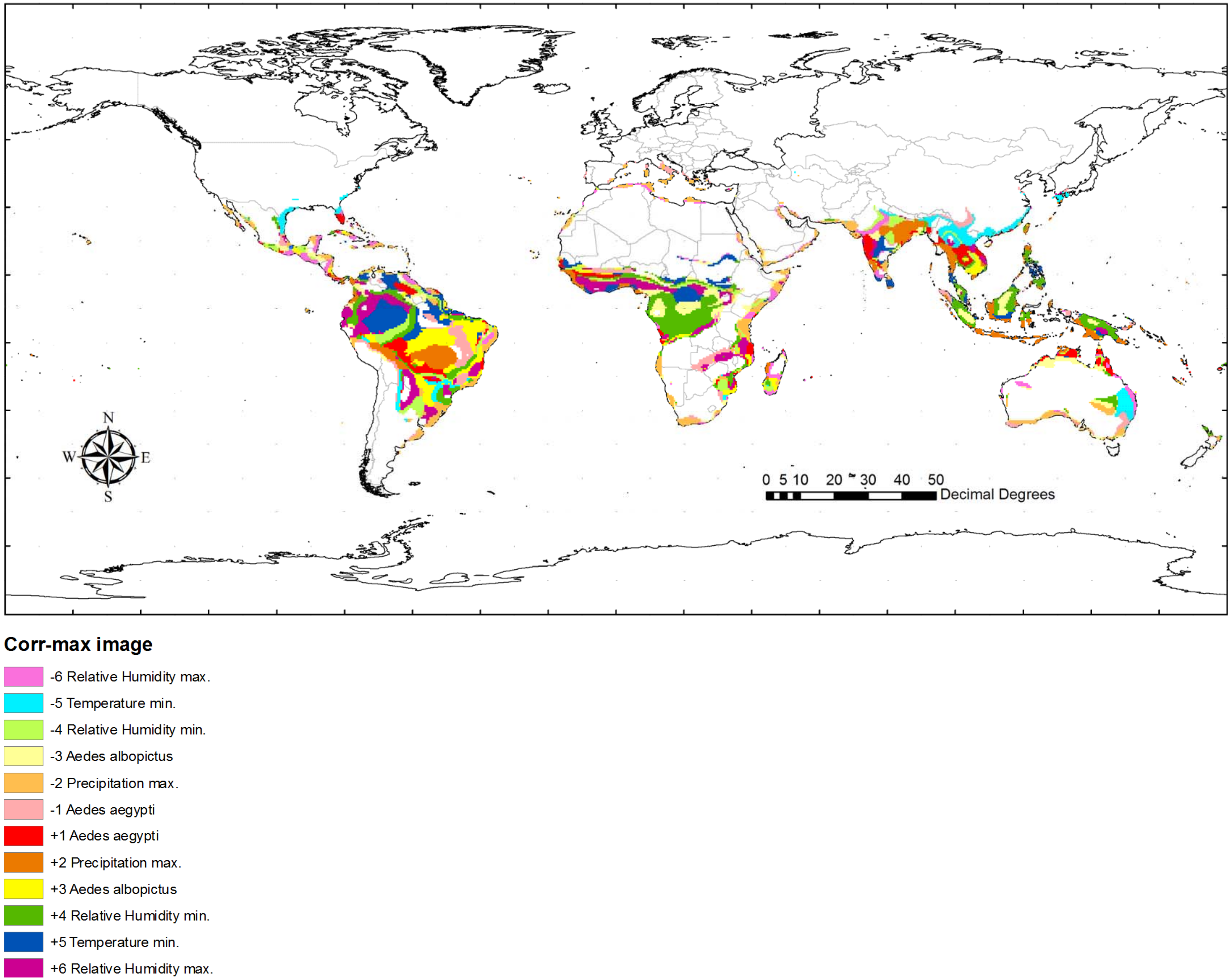

Figure 5g. As figure 5d but only for areas predicted unsuitable for dengue at the present time (from figure 5a).

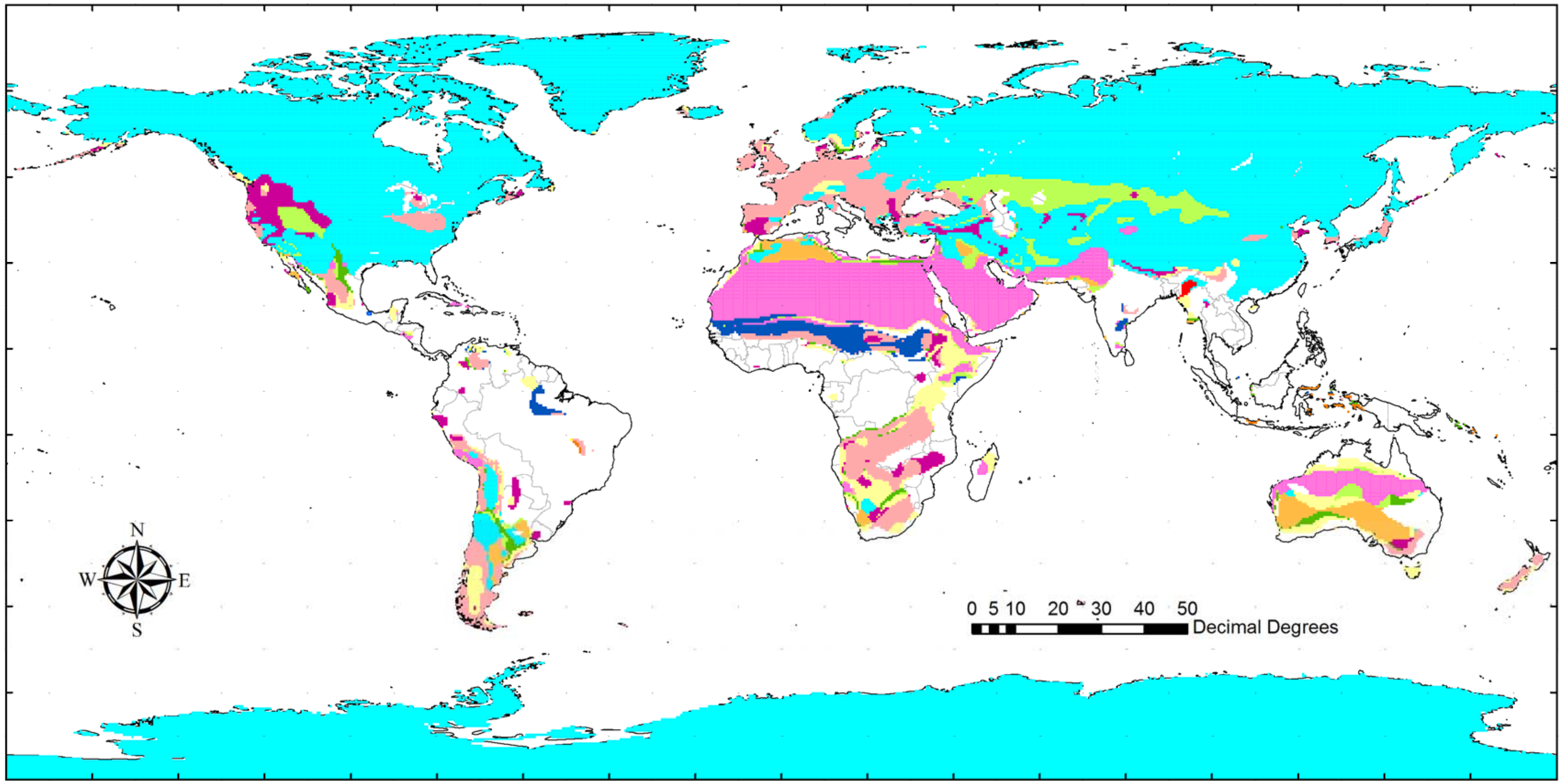

Corr-max image

- -6 Relative Humidity max.
- -5 Temperature min.
- -4 Relative Humidity min.
- -3 Aedes albopictus
- -2 Precipitation max.
- -1 Aedes aegypti
- +1 Aedes aegypti
- +2 Precipitation max.
- +3 Aedes albopictus
- +4 Relative Humidity min.
- +5 Temperature min.
- +6 Relative Humidity max.

Figure 5h. As figure 5e but only for areas predicted suitable for dengue in 2080 (from figure 5b).

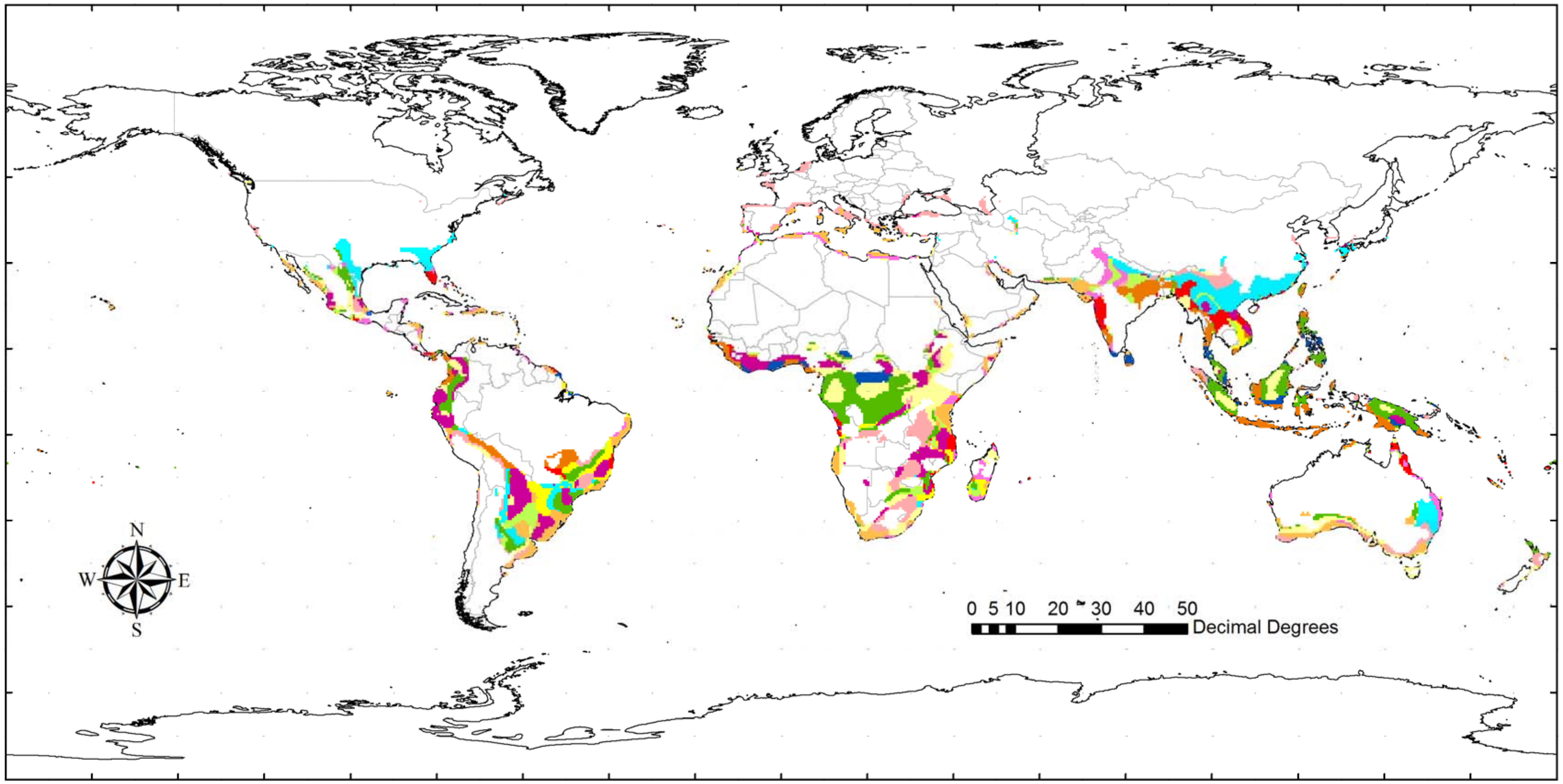

Corr-max image

- -6 Relative Humidity max.
- -5 Temperature min.
- -4 Relative Humidity min.
- -3 Aedes albopictus
- -2 Precipitation max.
- -1 Aedes aegypti
- +1 Aedes aegypti
- +2 Precipitation max.
- +3 Aedes albopictus
- +4 Relative Humidity min.
- +5 Temperature min.
- +6 Relative Humidity max.

Figure 5i. As figure 5e but only for areas predicted unsuitable for dengue in 2080 (from figure 5b).

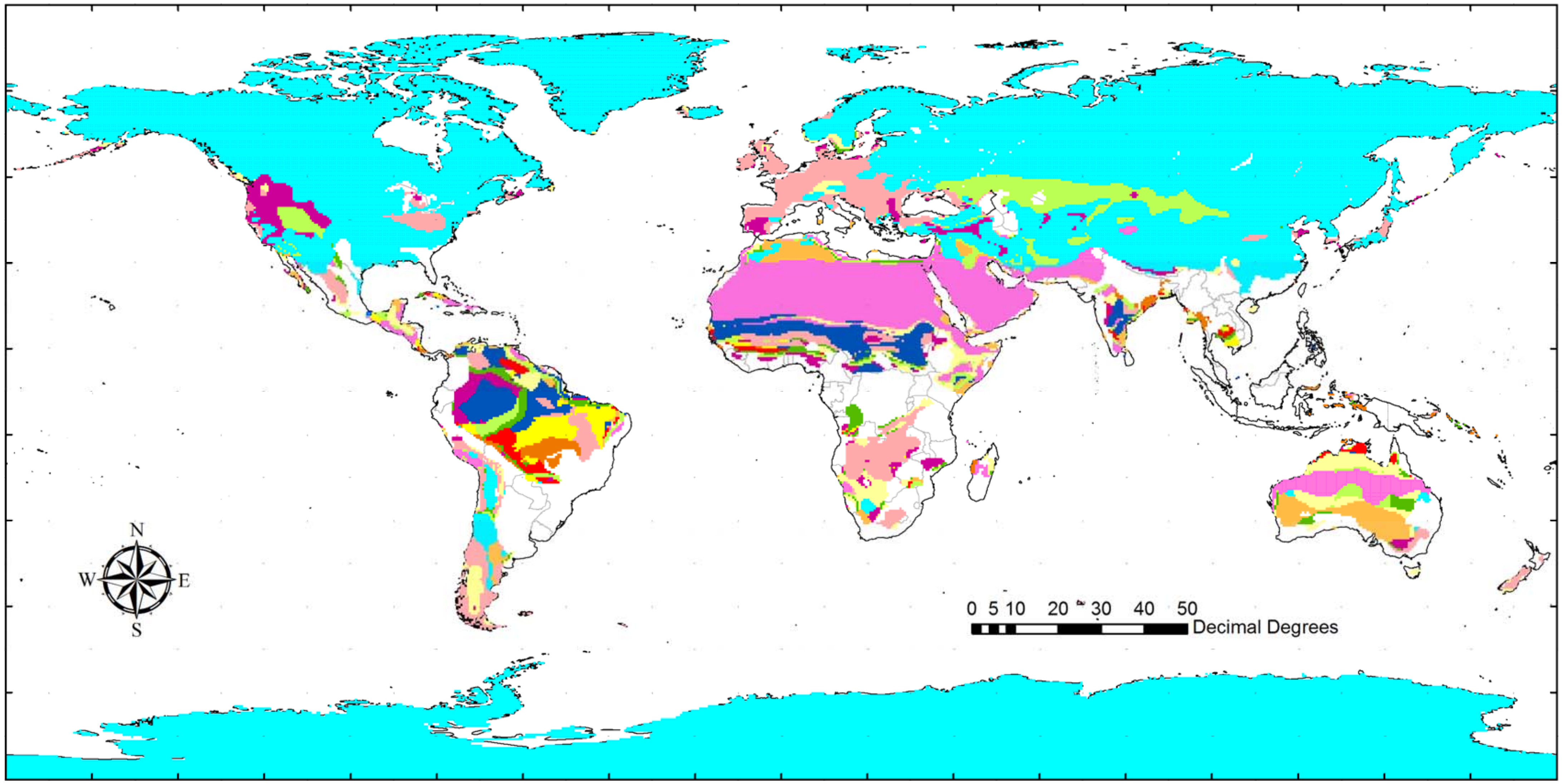

Corr-max image

- -6 Relative Humidity max.
- -5 Temperature min.
- -4 Relative Humidity min.
- -3 Aedes albopictus
- -2 Precipitation max.
- -1 Aedes aegypti
- +1 Aedes aegypti
- +2 Precipitation max.
- +3 Aedes albopictus
- +4 Relative Humidity min.
- +5 Temperature min.
- +6 Relative Humidity max.
